# Surgical site infection after gastrointestinal surgery in high-income, middle-income, and low-income countries: a prospective, international, multicentre cohort study

**DOI:** 10.1016/S1473-3099(18)30101-4

**Published:** 2018-05

**Authors:** GlobalSurg Collaborative, Aneel Bhangu, Adesoji O Ademuyiwa, Maria Lorena Aguilera, Philip Alexander, Sara W Al-Saqqa, Giuliano Borda-Luque, Ainhoa Costas-Chavarri, Thomas M Drake, Faustin Ntirenganya, J Edward Fitzgerald, Stuart J Fergusson, James Glasbey, JC Allen Ingabire, Lawani Ismaïl, Hosni Khairy Salem, Anyomih Theophilus Teddy Kojo, Marie Carmela Lapitan, Richard Lilford, Andre L Mihaljevic, Dion Morton, Alphonse Zeta Mutabazi, Dmitri Nepogodiev, Adewale O Adisa, Riinu Ots, Francesco Pata, Thomas Pinkney, Tomas Poškus, Ahmad Uzair Qureshi, Antonio Ramos-De la Medina, Sarah Rayne, Catherine A Shaw, Sebastian Shu, Richard Spence, Neil Smart, Stephen Tabiri, Ewen M Harrison, Chetan Khatri, Midhun Mohan, Zahra Jaffry, Afnan Altamini, Andrew Kirby, Kjetil Søreide, Gustavo Recinos, Jen Cornick, Maria Marta Modolo, Dushyant Iyer, Sebastian King, Tom Arthur, Sayeda Nazmum Nahar, Ade Waterman, Michael Walsh, Arnav Agarwal, Augusto Zani, Mohammed Firdouse, Tyler Rouse, Qinyang Liu, Juan Camilo Correa, Peep Talving, Mengistu Worku, Alexis Arnaud, Vassilis Kalles, Basant Kumar, Sunil Kumar, Radhian Amandito, Roy Quek, Luca Ansaloni, Ahmed Altibi, Donatas Venskutonis, Justas Zilinskas, Tomas Poskus, John Whitaker, Vanessa Msosa, Yong Yong Tew, Alexia Farrugia, Elaine Borg, Zineb Bentounsi, Tanzeela Gala, Ibrahim Al-Slaibi, Haya Tahboub, Osaid H Alser, Diego Romani, Sebestian Shu, Piotr Major, Aurel Mironescu, Matei Bratu, Amar Kourdouli, Aliyu Ndajiwo, Abdulaziz Altwijri, Mohammed Ubaid Alsaggaf, Ahmad Gudal, Al Faifi Jubran, Sam Seisay, Bettina Lieske, Irene Ortega, Jenifa Jeyakumar, Kithsiri J Senanayake, Omar Abdulbagi, Yucel Cengiz, Dmitri Raptis, Yuksel Altinel, Chia Kong, Ella Teasdale, Gareth Irwin, Michael Stoddart, Rakan Kabariti, Sukrit Suresh, Katherine Gash, Ragavan Narayanan, Mayaba Maimbo, Besmir Grizhja, Shpetim Ymeri, Gezim Galiqi, Roberto Klappenbach, Diego Antezana, Alvaro Enrique Mendoza Beleño, Cecilia Costa, Belen Sanchez, Susan Aviles, Claudio Gabriel Fermani, Rubén Balmaceda, Santiago Villalobos, Juan Manuel Carmona, Daniel Hamill, Peter Deutschmann, Simone Sandler, Daniel Cox, Ram Nataraja, Claire Sharpin, Damir Ljuhar, Demi Gray, Morgan Haines, Dush Iyer, Nithya Niranjan, Scott D'Amours, Morvarid Ashtari, Helena Franco, Ashrarur Rahman Mitul, Sabbir Karim, Nowrin F Aman, Mahnuma Mahfuz Estee, Umme Salma, Joyeta Razzaque, Tasnia Hamid Kanta, Sayeeda Aktar Tori, Shadid Alamin, Swapnil Roy, Shadid Al Amin, Rezaul Karim, Muhtarima Haque, Amreen Faruq, Farhana Iftekhar, Margaret O'Shea, Greg Padmore, Ramesh Jonnalagadda, Andrey Litvin, Aliaksandr Filatau, Dzmitry Paulouski, Maryna Shubianok, Tatsiana Shachykava, Dzianis Khokha, Vladimir Khokha, Fernande Djivoh, Francis Dossou, Djifid Morel Seto, Dansou Gaspard Gbessi, Bruno Noukpozounkou, Yacoubou Imorou Souaibou, Kpèmahouton René Keke, Fred Hodonou, Ernest Yemalin Stephane Ahounou, Thierry Alihonou, Max Dénakpo, Germain Ahlonsou, Alemayehu Ginbo Bedada, Carlos Nsengiyumva, Sandrine Kwizera, Venerand Barendegere, Philip Choi, Simon Stock, Luai Jamal, Georges Azzie, Sameer Kushwaha, Tzu-Ling Chen, Chingwan Yip, Irene Montes, Felipe Zapata, Sebastian Sierra, Maria Isabel Villegas Lanau, Maria Clara Mendoza Arango, Ivan Mendoza Restrepo, Ruben Santiago Restrepo Giraldo, Edgar Domini, Robert Karlo, Jakov Mihanovic, Mohamed Youssef, Hossam Elfeki, Waleed Thabet, Aly Sanad, Gehad Tawfik, Ahmed Zaki, Noran Abdel-Hameed, Mohamed Mostafa, Muhammad Fathi Waleed Omar, Ahmed Ghanem, Emad Abdallah, Adel Denewer, Eman Emara, Eman Rashad, Ahmad Sakr, Rehab Elashry, Sameh Emile, Toqa Khafagy, Sara Elhamouly, Arwa Elfarargy, Amna Mamdouh Mohamed, Ghada Saied Nagy, Abeer Esam, Eman Elwy, Aya Hammad, Salwa Khallaf, Eman Ibrahim, Ahmed Said Badr, Ahmed Moustafa, Amany Eldosouky Mohammed, Mohammed Elgheriany, Eman Abdelmageed, Eman Abd Al Raouf, Esraa Samir Elbanby, Maha Elmasry, Mahitab Morsy Farahat, Eman Yahya Mansor, Eman Magdy Hegazy, Esraa Gamal, Heba Gamal, Hend Kandil, Doaa Maher Abdelrouf, Mohamed Moaty, Dina Gamal, Nada El-Sagheer, Mohamed Salah, Salma Magdy, Asmaa Salah, Ahmed Essam, Ahmed Ali, Mahmoud Badawy, Sara Ahmed, Mazed Mohamed, Abdelrahman Assal, Mohamed Sleem, Mai Ebidy, Aly Abd-Elrazek, Diaaaldin Zahran, Nourhan Adam, Mohamed Nazir, Adel B Hassanein, Ahmed Ismail, Amira Elsawy, Rana Mamdouh, Mohamed Mabrouk, Lopna Ahmed Mohamed Ahmed, Mohamed Hassab Alnaby, Eman Magdy, Manar Abd-Elmawla, Marwan Fahim, Bassant Mowafy, Moustafa Ibrahim Mahmoud, Meran Allam, Muhammad Alkelani, Noran Halim El Gendy, Mariam Saad Aboul-Naga, Reham Alaa El-Din, Alyaa Halim Elgendy, Mohamed Ismail, Mahmoud Shalaby, Aya Adel Elsharkawy, Mahmoud Elsayed Moghazy, Khaled Hesham Elbisomy, Hend Abdel Gawad Shakshouk, Mohamed Fouad Hamed, Mai Mohamed Ebidy, Mostafa Abdelkader, Mohamed Karkeet, Hayam Ahmed, Israa Adel, Mohammad Elsayed Omar, Mohamed Ibrahim, Omar Ghoneim, Omar Hesham, Shimaa Gamal, Karim Hilal, Omar Arafa, Sawsan Adel Awad, Menatalla Salem, Fawzia Abdellatif Elsherif, Nourhan Elsabbagh, Moustafa R Aboelsoud, Ahmed Hossam Eldin Fouad Rida, Amr Hossameldin, Ethar Hany, Yomna Hosny Asar, Nourhan Anwar, Mohamed Gadelkarim, Samar Abdelhady, Eman Mohamed Morshedy, Reham Saad, Nourhan Soliman, Mahmoud Salama, Eslam Ezzat, Arwa Mohamed, Arwa Ibrahim, Alaa Fergany, Sara Mohammed, Aya Reda, Yomna Allam, Hanan Adel Saad, Afnan Abdelfatah, Aya Mohamed Fathy, Ahmed El-Sehily, Esraa Abdalmageed Kasem, Ahmed Tarek Abdelbaset Hassan, Ahmed Rabeih Mohammed, Abdalla Gamal Saad, Yasmin Elfouly, Nesma Elfouly, Arij Ibrahim, Amr Hassaan, Mohammed Mustafa Mohammed, Ghada Elhoseny, Mohamed Magdy, Esraa Abd Elkhalek, Yehia Zakaria, Tarek Ezzat, Ali Abo El Dahab, Mohamed Kelany, Sara Arafa, Osama Mokhtar Mohamed Hassan, Nermin Mohamed Badwi, Ahmad Saber Sleem, Hussien Ahmed, Kholoud Abdelbadeai, Mohamed Abozed Abdullah, Muhammad Amsyar Auni Lokman, Suraya Bahar, Anan Rady Abdelazeam, Abdelrahman Adelshone, Muhammad Bin Hasnan, Athirah Zulkifli, Siti Nur Alia Kamarulzamil, Abdelaziz Elhendawy, Aliang Latif, Ahmad Bin Adnan, Shahadatul Shaharuddin, Aminah Hanum Haji Abdul Majid, Mahmoud Amreia, Dina Al-Marakby, Mahmoud Salma, Mohamad Jeffrey Bin Ismail, Elissa Rifhan Mohd Basir, Citra Dewi Mohd Ali, Aya Yehia Ata, Maha Nasr, Asmaa Rezq, Ahmed Sheta, Sherif Tariq, Abd Elkhalek Sallam, Abdelrhman KZ Darwish, Sohaila Elmihy, Shady Elhadry, Ahmed Farag, Haidar Hajeh, Abdelaziz Abdelaal, Amro Aglan, Ahmed Zohair, Mahitab Essam, Omar Moussa, Esraa El-Gizawy, Mostafa Samy, Safia Ali, Esraa El Halawany, Ahmed Ata, Mohamed El Halawany, Mohamed Nashat, Samar Soliman, Alaa Elazab, Mostada Samy, Mohamed A Abdelaziz, Khaled Ibrahim, Ahmed Mohamed Ibrahim, Ammar Gado, Usama Hantour, Esraa Alm Eldeen, Mohamed Reda Loaloa, Arwa Abouzaid, Mostafa Ahmed Bahaa Eldin, Eman Hashad, Fathy Sroor, Doaa Gamil, Eman Mahmoud Abdulhakeem, Mahmoud Zakaria, Fawzy Mohamed, Marwan Abubakr, Elsayed Ali, Hesham Magdy, Menna Tallah Ramadan, Mohamed Abdelaty Mohamed, Salma Mansour, Hager Abdul Aziz Amin, Ahmed Rabie Mohamed, Mahmoud Saami, Nada Ahmed Reda Elsayed, Adham Tarek, Sabry Mohy Eldeen Mahmoud, Islam Magdy El Sayed, Amira Reda, Martina Yusuf Shawky, Mohammed Mousa Salem, Shahinaz Alaa El-Din, Noha Abdullah Soliman, Muhammed Talaat, Shahinaz Alaael-Dein, Ahmed Abd Elmoen Elhusseiny, Noha Abdullah, Mohammed Elshaar, Aya Abdelfatah Ibraheem, Hager Abdulaziz, Mohammed Kamal Ismail, Mona Hamdy Madkor, Mohamed Abdelaty, Sara Mahmoud Abdel-Kader, Osama Mohamed Salah, Mahmoud Eldafrawy, Ahmed Zaki Eldeeb, Mostafa Mahmoud Eid, Attia Attia, Khalid Salah El-Dien, Ayman Shwky, Mohamed Adel Badenjki, Abdelrahman Soliman, Samaa Mahmoud Al Attar, Farrag Sayed, Fahd Abdel Sabour, Mohammed G Azizeldine, Muhammad Shawqi, Abdullah Hashim, Ahmed Aamer, Ahmed Mahmoud Abdelraouf, Mahmoud Abdelshakour, Amal Ibrahim, Basma Mahmoud, Mohamed Ali Mahmoud, Mostafa Qenawy, Ahmed M Rashed, Ahmed Dahy, Marwa Sayed, Ahmed W Shamsedine, Bakeer Mohamed, Ahmad Hasan, Mahmoud M Saad, Khalil Abdul Bassit, Nadia Khalid Abd El-Latif, Nada Elzahed, Ahmed El Kashash, Nada Mohamed Bekhet, Sarah Hafez, Ahmed Gad, Mahmoud Elkhadragy Maher, Ahmed Abd El-Sameea, Mohamed Hafez, Ahmad Sabe, Ataa Ahmed, Ahmed Shahine, Khaled Dawood, Shireen Gaafar, Reem Husseiny, Omnia Aboelmagd, Ahmed Soliman, Nourhan Mesbah, Hossam Emadeldin, Amgad Al Meligy, Amira Hassan Bekhet, Doaa Hasan, Khaled Alhady, Ahmad Khaled Sabe, Mahmoud A Elnajjar, Majed Aboelella, Ward Hamsho, Ihab Hassan, Hala Saad, Galaleldin Abdelazim, Hend Mahmoud, Noha Wael, Ahmedali M Kandil, Ahmed Magdy, Shimaa Said Elkholy, Badr Eldin Adel, Kareem Dabbour, Saged Elsherbiney, Omar Mattar, Abdulshafi Khaled AbdRabou, Mohammed Yahia Mohamed Aly, Abdelrahman Geuoshy, Ahmedglal Elnagar, Saraibrahim Ahmed, Ibrahem Abdelmotaleb, Amr Ahmed Saleh, Hesham Mohammed Bakry, Manar Saeed, Shady Mahmoud, Badreldin Adel Tawfik, Samar Adel Ismail, Esraay Zakaria, Mariam O Gad, Mohamed Salah Elhelbawy, Monica Bassem, Noha Maraie, Nourhan Medhat Elhadary, Nourhan Semeda, Shaza Rabie Mohamed, Hesham Mohammed Bakry, AA Essam, Dina Tarek, Khlood Ashour, Alaa Elhadad, Abdulrahman Abdel-Aty, Ibrahim Rakha, Sara Mamdouh Matter, Rasha Abdelhamed, Omar Abdelkader, Ayat Hassaan, Yasmin Soliman, Amna Mohamed, Sara Ghanem, Sara Amr Mohamed Farouk, Eman Mohamed Ibrahim, Esraa El-Taher, Merna Mostafa, Mohamed Fawzy Mahrous Badr, Rofida Elsemelawy, Aya El-Sawy, Ahmad Bakr, Ahmad Abdel Razaq Al Rafati, Sten Saar, Arvo Reinsoo, Nebyou Seyoum, Tewodros Worku, Agazi Fitsum, Matti Tolonen, Ari Leppäniemi, Ville Sallinen, Benogreît Parmentier, Matthieu Peycelon, Sabine Irtan, Sabrina Dardenne, Elsa Robert, Betty Maillot, Etienne Courboin, Alexis Pierre Arnaud, Juliette Hascoet, Olivier Abbo, Amir Ait Kaci, Thomas Prudhomme, Quentin Ballouhey, Céline Grosos, Laurent Fourcade, Tolg Cecilia, Colombani Jean-Francois, Francois-Coridon Helene, Xavier Delforge, Elodie Haraux, Bertrand Dousset, Roberto Schiavone, Sebastien Gaujoux, Jean-Baptiste Marret, Aurore Haffreingue, Julien Rod, Mariette Renaux-Petel, Jean-François Lecompte, Jean Bréaud, Pauline Gastaldi, Chouikh Taieb, Raquillet Claire, Echaieb Anis, Nasir Bustangi, Manuel Lopez, Aurelien Scalabre, Maria Giovanna Grella, Aurora Mariani, Guillaume Podevin, Françoise Schmitt, Erik Hervieux, Aline Broch, Cecile Muller, Dickson Bandoh, Francis Abantanga, Martin Kyereh, Hamza Asumah, Eric Kofi Appiah, Paul Wondoh, Adam Gyedu, Charles Dally, Kwabena Agbedinu, Michael Amoah, Abiboye Yifieyeh, Kwabena Agbedinu, Frank Owusu, Mabel Amoako-Boateng, Makafui Dayie, Richmond Hagan, Sam Debrah, Micheal Ohene-Yeboah, Joe-Nat Clegg-Lampety, Victor Etwire, Jonathan Dakubo, Samuel Essoun, William Bonney, Hope Glover-Addy, Samuel Osei-Nketiah, Joachim Amoako, Niiarmah Adu-Aryee, William Appeadu-Mensah, Antoinette Bediako-Bowan, Florence Dedey, Mattew Ekow, Emmanuel Akatibo, Musah Yakubu, Hope Edem Kofi Kordorwu, Kwasi Asare-Bediako, Enoch Tackie, Kenneth Aaniana, Emmanuel Acquah, Richard Opoku-Agyeman, Anthony Avoka, Kwasi Kusi, Kwame Maison, Frank Enoch Gyamfi, Gandau Naa Barnabas, Saiba Abdul-Latif, Philip Taah Amoako, Anthony Davor, Victor Dassah, Enoch Dagoe, Prince Kwakyeafriyie, Elliot Akoto, Eric Ackom, Ekow Mensah, Ebenezer Takyi Atkins, Christian Lari Coompson, Nikolaos Ivros, Christoforos Ferousis, Vasileios Kalles, Christos Agalianos, Ioannis Kyriazanos, Christos Barkolias, Angelos Tselos, Georgios Tzikos, Evangelos Voulgaris, Dimitrios Lytras, Athanasia Bamicha, Kyriakos Psarianos, Anastasios Stefanopoulos, Ioannis Patoulias, Dimitrios Sfougaris, Ioannis Valioulis, Dimitrios Balalis, Dimitrios Korkolis, Dimitrios K Manatakis, Georgios Kyrou, Georgios Karabelias, Iason-Antonios Papaskarlatos, Kolonia Konstantina, Nikolaos Zampitis, Stylianos Germanos, Aspasia Papailia, Theodosios Theodosopoulos, Georgios Gkiokas, Magdalini Mitroudi, Christina Panteli, Thomas Feidantsis, Konstantinos Farmakis, Dimitrios Kyziridis, Orestis Ioannidis, Styliani Parpoudi, Georgios Gemenetzis, Stavros Parasyris, Christos Anthoulakis, Nikolaos Nikoloudis, Michail Margaritis, Maria-Lorena Aguilera-Arevalo, Otto Coyoy-Gaitan, Javier Rosales, Luis Tale, Rafael Soley, Emmanuel Barrios, Servio Tulio Torres Rodriguez, Carlos Paz Galvez, Danilo Herrera Cruz, Guillermo Sanchez Rosenberg, Alejandro Matheu, David Monterroso Cohen, Marie Paul, Angeline Charles, Justin Chak Yiu Lam, Man Hon Andrew Yeung, Chi Ying Jacquelyn Fok, Ka Hin Gabriel Li, Anthony Chuk-Him Lai, Yuk Hong Eric Cheung, Hong Yee Wong, Ka Wai Leung, Tien Seng Bryan Lee, Wai Him Lam, Weihei Dao, Stephanie Hiu-wai Kwok, Tsz-Yan Katie Chan, Yung Kok Ng, TWC Mak, Chi Chung Foo, James Yang, Ankur Bhatnagar, Vijaid Upadhyaya, Uday Muddebihal, Wasim Dar, KC Janardhan, Neerav Aruldas, Fidelis Jacklyn Adella, Anthonius Santoso Rulie, Ferdy Iskandar, Jonny Setiawan, Cicilia Viany Evajelista, Hani Natalie, Arlindawati Suyadi, Rudy Gunawan, Herlin Karismaningtyas, Lusi Padma Sulistianingsih Mata, Ferry Fitriya Ayu Andika, Afifatun Hasanah, T Ariani Widiastini, Nurlaila Ayu Purwaningsih, Annisa Dewi Fitriana Mukin, Dina Faizatur Rahmah, Hazmi Dwinanda Nurqistan, Hasbi Maulana Arsyad, Novia Adhitama, Wifanto Saditya Jeo, Nathania Sutandi, Audrey Clarissa, Phebe Anggita Gultom, Matthew Billy, Andreass Haloho, Nadya Johanna, Felix Lee, Radin Mohd Nurrahman Radin Dorani, Martha Glynn, Mohammad Alherz, Wennweoi Goh, Haaris A Shiwani, Lorraine Sproule, Kevin C Conlon, Miklosh Bala, Asaf Kedar, Luca Turati, Federica Bianco, Francesca Steccanella, Gaetano Gallo, Mario Trompetto, Giuseppe Clerico, Matteo Papandrea, Giuseppe Sammarco, Rosario Sacco, Angelo Benevento, Luisa Giavarini, Mariano Cesare Giglio, Luigi Bucci, Gianluca Pagano, Viviana Sollazzo, Roberto Peltrini, Gaetano Luglio, Arianna Birindelli, Salomone Di Saverio, Gregorio Tugnoli, Miguel Angel Paludi, Pietro Mingrone, Domenica Pata, Francesco Selvaggi, Lucio Selvaggi, Gianluca Pellino, Natale Di Martino, Gianluca Curletti, Paolo Aonzo, Raffaele Galleano, Stefano Berti, Elisa Francone, Silvia Boni, Laura Lorenzon, Annalisa lo Conte, Genoveffa Balducci, Gianmaria Confalonieri, Giovanni Pesenti, Laura Gavagna, Giorgio Vasquez, Simone Targa, Savino Occhionorelli, Dario Andreotti, Giacomo Pata, Andrea Armellini, Deborah Chiesa, Fabrizio Aquilino, Nicola Chetta, Arcangelo Picciariello, Mohamed Abdelkhalek, Andrea Belli, Silvia De Franciscis, Annamaria Bigaran, Alessandro Favero, Stefano MM Basso, Paola Salusso, Martina Perino, Sylvie Mochet, Diego Sasia, Francesco Riente, Marco Migliore, David Merlini, Silvia Basilicò, Carlo Corbellini, Veronica Lazzari, Yuri Macchitella, Luigi Bonavina, Daniele Angelieri, Diego Coletta, Federica Falaschi, Marco Catani, Claudia Reali, Mariastella Malavenda, Celeste Del Basso, Sergio Ribaldi, Massimo Coletti, Andrea Natili, Norma Depalma, Immacolata Iannone, Angelo Antoniozzi, Davide Rossi, Daniele Gui, Gerardo Perrotta, Matteo Ripa, Francesco Ruben Giardino, Maurizio Foco, Erika Vicario, Federico Coccolini, Gabriela Elisa Nita, Nicoletta Leone, Andrea Bondurri, Anna Maffioli, Andrea Simioni, Davide De Boni, Sandro Pasquali, Elena Goldin, Elena Vendramin, Eleonora Ciccioli, Umberto Tedeschi, Luca Bortolasi, Paola Violi, Tommaso Campagnaro, Simone Conci, Giovanni Lazzari, Calogero Iacono, Alfredo Gulielmi, Serena Manfreda, Anna Rinaldi, Maria Novella Ringressi, Beatrice Brunoni, Giuseppe Salamone, Mirko Mangiapane, Paolino De Marco, Antonella La Brocca, Roberta Tutino, Vania Silvestri, Leo Licari, Tommaso Fontana, Nicolò Falco, Gianfranco Cocorullo, Mostafa Shalaby, Pierpaolo Sileri, Claudio Arcudi, Isam Bsisu, Khaled Aljboor, Lana Abusalem, Aseel Alnusairat, Ahmad Qaissieh, Emad Al-Dakka, Ali Ababneh, Oday Halhouli, Taha Yusufali, Hussein Mohammed, Justus Lando, Robert Parker, Wairimu Ndegwa, Mantas Jokubauskas, Jolanta Gribauskaite, Justas Kuliavas, Audrius Dulskas, Narimantas E Samalavicius, Kristijonas Jasaitis, Audrius Parseliunas, Viktorija Nevieraite, Margarita Montrimaite, Evelina Slapelyte, Edvinas Dainius, Romualdas Riauka, Zilvinas Dambrauskas, Andrejus Subocius, Linas Venclauskas, Antanas Gulbinas, Saulius Bradulskis, Simona Kasputyte, Deimante Mikuckyte, Mindaugas Kiudelis, Tomas Jankus, Steponas Petrikenas, Matas Pažuskis, Zigmantas Urniežius, Mantas Vilčinskas, Vincas Jonas Banaitis, Vytautas Gaižauskas, Edvard Grisin, Povilas Mazrimas, Rokas Rackauskas, Mantas Drungilas, Karolis Lagunavicius, Vytautas Lipnickas, Dovilè Majauskyté, Valdemaras Jotautas, Tomas Abaliksta, Laimonas Uščinas, Gintaras Simutis, Adomas Ladukas, Donatas Danys, Erikas Laugzemys, Saulius Mikalauskas, Elena Zdanyte Sruogiene, Petras Višinskas, Reda Žilinskienė, Deividas Dragatas, Andrius Burmistrovas, Zygimantas Tverskis, Arturas Vaicius, Ruta Mazelyte, Antanas Zadoroznas, Nerijus Kaselis, Greta Žiubrytė, Finaritra Casimir Fleur Prudence Rahantasoa, Luc Hervé Samison, Fanjandrainy Rasoaherinomenjanahary, Todisoa Emmanuella Christina Tolotra, Cornelius Mukuzunga, Chimwemwe Kwatiwani, Nelson Msiska, Feng Yih Chai, Siti Mohd Desa Asilah, Khuzaimah Zahid Syibrah, Pui Xin Chin, Afizah Salleh, Nur Zulaika Riswan, April Camilla Roslani, Hoong-Yin Chong, Nora Abdul Aziz, Keat-Seong Poh, Chu-Ann Chai, Sandip Kumar, Mustafa Mohammed Taher, Nik Ritza Kosai, Dayang Nita Abdul Aziz, Reynu Rajan, Rokayah Julaihi, Durvesh Lacthman Jethwani, Muhammad Taqiyuddin Yahaya, Nik Azim Nik Abdullah, Susan Wndy Mathew, Kuet Jun Chung, Milaksh Kumar Nirumal, R Goh Ern Tze, Syed Abdul Wahhab Eusoffee Wan Ali, Yiing Yee Gan, Jesse Ron Swire Ting, Samuel S Y Sii, Kean Leong Koay, Yi Koon Tan, Alvin Ee Zhiun Cheah, Chui Yee Wong, Tuan Nur'Azmah Tuan Mat, Crystal Yern Nee Chow, Prisca AL Har, Yishan Der, Fitjerald Henry, Xinwei Low, Ya Theng Neo, Hian Ee Heng, Shu Ning Kong, Cheewei Gan, Yi Ting Mok, Yee Wen Tan, Kandasami Palayan, Mahadevan Deva Tata, Yih Jeng Cheong, Kuhaendran Gunaseelan, Wan Nurul 'Ain Wan Mohd Nasir, Pigeneswaren Yoganathan, Eu Xian Lee, Jian Er Saw, Li Jing Yeang, Pei Ying Koh, Shyang Yee Lim, Shuang Yi Teo, Nicole Grech, Daniela Magri, Kristina Cassar, Christine Mizzi, Malcolm Falzon, Nihaal Shaikh, Ruth Scicluna, Stefan Zammit, Sean Mizzi, Svetlana Doris Brincat, Thelma Tembo, Vu Thanh Hien Le, Tara Grima, Keith Sammut, Kurt Carabott, Ciskje Zarb, Andre Navarro, Thea Dimech, Georgette Marie Camilleri, Isaac Bertuello, Jeffrey Dalli, Karl Bonavia, Samantha Corro-Diaz, Marisol Manriquez-Reyes, Amina Abdelhamid, Abdelmalek Hrora, Sarah Benammi, Houda Bachri, Meryem Abbouch, Khaoula Boukhal, Redouane Mammar Bennai, Abdelkader Belkouchi, Mohamed Sobhi Jabal, Chaymae Benyaiche, Maarten Vermaas, Lucia Duinhouwer, Javier Pastora, Greta Wood, Maria Soledad Merlo, Akinlabi Ajao, Omobolaji Ayandipo, Taiwo Lawal, Abdussemiu Abdurrazzaaq, Muslimat Alada, Abdulrasheed Nasir, James Adeniran, Olufemi Habeeb, Ademola Popoola, Ademola Adeyeye, Ademola Adebanjo, Opeoluwa Adesanya, Adewale Adeniyi, Henry Mendel, Bashir Bello, Umar Muktar, Adedapo Osinowo, Thomas Olagboyega Olajide, Oyindamola Oshati, George Ihediwa, Babajide Adenekan, Victor Nwinee, Felix Alakaloko, Olumide Elebute, Abdulrazzaq Lawal, Chris Bode, Mojolaoluwa Olugbemi, Alaba Adesina, Olubukola Faturoti, Oluwatomi Odutola, Oluwaseyi Adebola, Clement Onuoha, Ogechukwu Taiwo, Omolara Williams, Fatai Balogun, Olalekan Ajai, Mobolaji Oludara, Iloba Njokanma, Roland Osuoji, Stephen Kache, Jonathan Ajah, Jerry Makama, Ahmed Adamu, Suleiman Baba, Mohammad Aliyu, Shamsudeen Aliyu, Yahaya Ukwenya, Halima Aliyu, Tunde Sholadoye, Muhammad Daniyan, Oluseyi Ogunsua, Lofty-John Anyanwu, Abdurrahaman Sheshe, Aminu Mohammad, Samson Olori, Philip Mshelbwala, Babatunde Odeyemi, Garba Samson, Oyediran Kehinde Timothy, Sani Ali Samuel, Anthony Ajiboye, Isaac Amole, Olajide Abiola, Akin Olaolorun, Torhild Veen, Arezo Kanani, Kristian Styles, Ragnar Herikstad, Johannes Wiik Larsen, Jon Arne Søreide, Elisabeth Jensen, Mads Gran, Eirik Kjus Aahlin, Tina Gaarder, Peter Wiel Monrad-Hansen, Pål Aksel Næss, Giedrius Lauzikas, Joachim Wiborg, Silje Holte, Knut Magne Augestad, Gurpreet Singh Banipal, Michela Monteleone, Thomas Tetens Moe, Johannes Kurt Schultz, Najwa Nadeem, Muhammad Saqlain, Jibran Abbasy, Abdul Rehman Alvi, Noman Shahzad, Kamran Faisal Bhopal, Zainab Iftikhar, Muhammad Talha Butt, Syed Asaat ul Razi, Asdaq Ahmed, Ali Khan Niazi, Ibrahim Raza, Fatima Baluch, Ahmed Raza, Ahmad Bani-Sadar, Muhammad Adil, Awais Raza, Mahnoor Javaid, Muhammad Waqar, Maryam Ali Khan, Mohammad Mohsin Arshad, Mohammad Asim Amjad, Taher Al-taher, Ayah Hamdan, Ayman Salman, Rana Saadeh, Aseel Musleh, Dana Jaradat, Soha Abushamleh, Sakhaa Hanoun, Amjad Abu Qumbos, Aseel Hamarshi, Ayman And Taher, Israa Qawasmi, Khalid Qurie, Marwa Altarayra, Mohammad Ghannam, Alaa Shaheen, Azher Herebat, Aram Abdelhaq, Ahmad Shalabi, Maram Abu-toyour, Fatema Asi, Ala Shamasneh, Anwar Atiyeh, Mousa Mustafa, Rula Zaa'treh, Majd Dabboor, Enas Alaloul, Heba Baraka, Jehad Meqbil, Alaa Al-Buhaisi, Mohamedraed Elshami, Samah Afana, Sahar Jaber, Said Alyacoubi, Yousef Abuowda, Tasneem Idress, Eman Abuqwaider, Sara Al-saqqa, Alaa Bowabsak, Alaa El Jamassi, Doaa Hasanain, Hadeel Al-Farram, Maram Salah, Aya Firwana, Marwa Hamdan, Israa Awad, Ahmad Ashour, Fayez Elian Al Barrawi, Ahmed Alkhatib, Maha Al-Faqawi, Mohamed Fares, Amjad Elmashala, Mohammad Adawi, Ihdaa Adawi, Reem Khreishi, Rose Khreishi, Ahmad Ashour, Ahed Ghaben, Gustavo Miguel Machain Vega, Jorge Torres Cardozo, Marcelo O'Higgins Roche, Gustavo Rodolfo Pertersen Servin, Helmut Alfredo Segovia Lohse, Larissa Ines Páez Lopez, Ramón Augusto Melo Cardozo, Fernando Espinoza, Angel David Pérez Rojas, Diana Sanchez, Camila Sanchez Samaniego, Shalon Guevara Torres, Alexander Canta Calua, Cesar Razuri, Nadia Ortiz, Xianelle Rodriguez, Nahilia Carrasco, Fridiz Saravia, Hector Shibao Miyasato, María Valcarcel-Saldaña, Ysabel Esthefany Alejos Bermúdez, Juan Carpio, Walter Ruiz Panez, Pedro Angel Toribio Orbegozo, Carolina Guzmán Dueñas, Kevin Turpo Espinoza, Ana Maria Sandoval Barrantes, Jorge Armando Chungui Bravo, Lorena Fuentes-Rivera, Carmen Fernández, Bárbara Málaga, Joselyn Ye, Ricardo Velasquez, Jannin Salcedo, Ana Lucia Contreras-Vergara, Angelica Genoveva Vergara Mejia, Maria Soledad Gonzales Montejo, Marilia del Carmen Escalante Salas, Willy Alcca Ticona, Marvin Vargas, George Christian Manrique Sila, Robinson Mas, Arazzelly del Pilar Paucar, Armando José Román Velásquez, Alina Robledo-Rabanal, Ludwing Alexander Zeta Solis, Kenny Turpo Espinoza, José Luis Hamasaki Hamaguchi, Erick Samuel Florez Farfan, Linda Alvi Madrid Barrientos, Juan Jaime Herrera Matta, John Jemuel V Mora, Menold Archee P Redota, Manuel Francisco Roxas, Maria Jesusa B Maño, Marie Dione Parreno-Sacdalan, Christel Leanne Almanon, Maciej Walędziak, Rafał Roszkowski, Michał Janik, Anna Lasek, Dorota Radkowiak, Mateusz Rubinkiewicz, Cristina Fernandes, Jose Costa-Maia, Renato Melo, Liviu Muntean, Aurel Sandu Mironescu, Lucian Corneliu Vida, Mariuca Popa, Hogea Mircea, Mihaela Vartic, Bogdan Diaconescu, Matei Razvan Bratu, Ionut Negoi, Mircea Beuran, Cezar Ciubotaru, Norbert Uzabumwana, Dieudonne Duhoranenayo, Elio Jovine, Nicola Zanini, Giovanni Landolfo, Murad Aljiffry, Faisal Idris, Mohammed Saleh A Alghamdi, Ashraf Maghrabi, Abdulmalik Altaf, Aroub Alkaaki, Ahmad Khoja, Abrar Nawawi, Sondos Turkustani, Eyad Khalifah, Adel Albiety, Sarah Sahel, Reham Alshareef, Mohammed Najjar, Ahmed Alzahrani, Ahmed Alghamdi, Wedyan Alhazmi, Ghiath Al Saied, Mohammed Alamoudi, Muhammed Masood Riaz, Mazen Hassanain, Basmah Alhassan, Abdullah Altamimi, Reem Alyahya, Norah Al Subaie, Fatema Al Bastawis, Afnan Altamimi, Thamer Nouh, Roaa Khan, Milan Radojkovic, Ljiljana Jeremic, Milica Nestorovic, Jia Hao Law, Keith Say Kwang Tan, Ryan Choon Kiat Tan, Joel Kin Tan, Lau Wen Liang Joel, Xue Wei Chan, Faith Qi Hui Leong, Choon Seng Chong, Sharon Koh, Kai Yin Lee, Kuok Chung Lee, Kent Pluke, Britta Dedekind, Puyearashid Nashidengo, Mark Ian Hampton, Johanna Joosten, Sanju Sobnach, Liana Roodt, Anthony Sander, James Pape, Niveshni Maistry, Phumudzo Ndwambi, Kamau Kinandu, Myint Tun, Frederick Du Toit, Quinn Ellison, Sule Burger, DC Grobler, Lawrence Bongani Khulu, Rachel Moore, Vicky Jennings, Astrid Leusink, Nazmie Kariem, Juan Gouws, Kathryn Chu, Heather Bougard, Fazlin Noor, Angela Dell, Stephanie Van Straten, Arvin Khamajeet, Serge Kapenda Tshisola, Kalangu Kabongo, Victor Kong, Yoshan Moodley, Frank Anderson, Thandinkosi Madiba, Flip du Plooy, Leila Hartford, Gareth Chilton, Parveen Karjiker, Matlou Ernest Mabitsela, Sibongile Ruth Ndlovu, Maria Badicel, Robert Jaich, Jaime Ruiz-Tovar, Luis Garcia-Florez, Jorge L Otero-Díez, Virginia Ramos Pérez, Nuria Aguado Suárez, Javier Minguez García, Sara Corral Moreno, Maria Vicenta Collado, Virginia Jiménez Carneros, Javier García Septiem, Mariana Gonzalez, Antonio Picardo, Enrique Esteban, Esther Ferrero, Eloy Espin-Basany, Ruth Blanco-Colino, Valeria Andriola, Lorena Solar García, Elisa Contreras, Carmen García Bernardo, Janet Pagnozzi, Sandra Sanz, Alberto Miyar de León, Asnel Dorismé, Joseluis Rodicio, Aida Suarez, Jessica Stuva, Tamara Diaz Vico, Laura Fernandez-Vega, Carla Soldevila-Verdeguer, Fatima Sena-Ruiz, Natalia Pujol-Cano, Paula Diaz-Jover, José Maria Garcia-Perez, Juan Jose Segura-Sampedro, Cristina Pineño-Flores, David Ambrona-Zafra, Andrea Craus-Miguel, Patricia Jimenez-Morillas, Angela Mazzella, AB Jayathilake, SPB Thalgaspitiya, LS Wijayarathna, PMSN Wimalge, Hakeem Ayomi Sanni, Ogheneochuko Okenabirhie, Anmar Homeida, Abobaker Younis, Omer Abdelbagi Omer, Mustafa Abdulaziz, Ali Mussad, Ali Adam, Ida Björklund, Sandra Ahlqvist, Anders Thorell, Fredrik Wogensen, Arestis Sokratous, Michaela Breistrand, Hildur Thorarinsdottir, Johanna Sigurdadottir, Maziar Nikberg, Abbas Chabok, Maria Hjertberg, Peter Elbe, Deborah Saraste, Wiktor Rutkowski, Louise Forlin, Karoliina Niska, Malin Sund, Dennis Oswald, Georgios Peros, Rafael Bluelle, Katharina Reinisch, Daniel Frey, Adrian Palma, Dimitri Aristotle Raptis, Lucius Zumbühl, Markus Zuber, Roger Schmid, Gabriela Werder, Antonio Nocito, Alexandra Gerosa, Silke Mahanty, Lukas Werner Widmer, Julia Müller, Alissa Gübeli, Grzegorz Zuk, Osman Bilgin Gulcicek, Talar Vartanoglu, Emin Kose, Servet Rustu Karahan, Mehmet Can Aydin, Nuri Alper Sahbaz, Ilkay Halicioglu, Halil Alis, Ipek Sapci, Can Adiyaman, Ahmet Murat Pektaş, Turgut Bora Cengiz, Ilkan Tansoker, Vedatcan Işler, Muazzez Cevik, Deniz Mutlu, Volkan Ozben, Berk Baris Ozmen, Sefa Bayram, Sinem Yolcu, Berna Buse Kobal, Ömer Faruk Toto, Haluk Cem Çakaloğlu, Kagan Karabulut, Vahit Mutlu, Bahar Busra Ozkan, Saban Celik, Anil Semiz, Selim Bodur, Enisburak Gül, Busra Murutoglu, Reyyan Yildirim, Bahadir Emre Baki, Ekin Arslan, Mehmet Ulusahin, Ali Guner, Kudir Tomas, Nathan Walker, Nikhita Shrimanker, Simon Cole, Ryan Breslin, Ravi Srinivasan, Mohamed Elshaer, Kristina Hunter, Ahmed Al-Bahrani, Ignatius Liew, Nora Grace Mairs, Alistair Rocke, Lachlan Dick, Mobeen Qureshi, Debkumar Chowdhury, Naomi Wright, Clare Skerritt, Dorothy Kufeji, Adrienne Ho, Tharindra Dissanayake, Athula Tennakoon, Wadah Ali, Shujing Jane Lim, Charlene Tan, Stephen O'Neill, Catrin Jones, Stephen Knight, Dima Nassif, Abhishek Sharma, Oliver Warren, Rebecca White, Aia Mehdi, Nathan Post, Eliana Kalakouti, Enkhbat Dashnyam, Frederick Stourton, Ioannis Mykoniatis, Chelise Currow, Francisca Wong, Ashish Gupta, Veeranna Shatkar, Joshua Luck, Suraj Kadiwar, Alexander Smedley, Rebecca Wakefield, Philip Herrod, James Blackwell, Jonathan Lund, Fraser Cohen, Ashwath Bandi, Stefano Giuliani, Giles Bond-Smith, Theodore Pezas, Neda Farhangmehr, Tomas Urbonas, Miklos Perenyei, Philip Ireland, Natalie Blencowe, Kirk Bowling, David Bunting, Lydia Longstaff, Kenneth Keogh, Hyunjin Jeon, Muhammad Rafaih Iqbal, Shivun Khosla, Anna Jeffery, James Perera, Ahmad Aboelkassem Ibrahem, Tariq Alhammali, Yahya Salama, Shaun Oram, Thomas Kidd, Fraser Cullen, Christopher Owen, Michael Wilson, Seehui Chiu, Hannah Sarafilovic, Jennifer Ploski, Elizabeth Evans, Athar Abbas, Sylvia Kamya, Norzawani Ishak, Carly Bisset, Cedar Andress, Ye Ru Chin, Priya Patel, David Evans, Aidan Haslegrave, Adam Boggon, Kirsten Laurie, Katie Connor, Thomas Mann, Anahita Mansuri, Rachel Davies, Ewen Griffiths, Aized Raza Shahbaz, Calvin Eng, Farhat Din, Ariadne L'Heveder, Esther HG Park, Ramanish Ravishankar, Kirsten McIntosh, Jih Dar Yau, Luke Chan, Susan McGarvie, Lingshan Tang, Hui Lim, Suhhuey Yap, Jay Park, Zhan Herr Ng, Shahrukh Mirza, Yun Lin Ang, Luke Walls, Chloe Roy, Simon Paterson-Brown, Julian Camilleri-Brennan, Kenneth Mclean, Michelle S D'Souza, Savva Pronin, David Ewart Henshall, Eunice Zuling Ter, Dina Fouad, Ashish Minocha, William English, Catrin Morgan, Dominic Townsend, Laura Maciejec, Shareef Mahdi, Onyinye Akpenyi, Elisabeth Hall, Hanaan Caydiid, Zakaria Rob, Tom Abbott, Hew D Torrance, Robin Johnston, Mohammed Aki Gani, Gianpiero Gravante, Shivanchan Rajmohan, Kiran Majid, Shiva Dindyal, Christopher Smith, Madanmohan Palliyil, Sanjay Patel, Luke Nicholson, Neil Harvey, Katie Baillie, Sam Shillito, Suzanne Kershaw, Rebecca Bamford, Peter Orton, Elke Reunis, Robert Tyler, Wai Cheong Soon, Guled M Jama, Dharminder Dhillon, Khyati Patel, Shayanthan Nanthakumaran, Rachel Heard, Kar Yan Chen, Behrad Barmayehvar, Uttaran Datta, Sivesh K Kamarajah, Sharad Karandikar, Sobhana Iftekhar Tani, Eimear Monaghan, Philippa Donnelly, Michael Walker, Jehangirshaw Parakh, Sarah Blacker, Anil Kaul, Arjun Paramasivan, Sameh Farag, Ashrafun Nessa, Salwa Awadallah, Jieqi Lim, James Chean Khun Ng, Ravi P Kiran, Alice Murray, Eric Etchill, Mohini Dasari, Juan Puyana, Nadeem Haddad, Martin Zielinski, Asad Choudhry, Celeste Caliman, Mieshia Beamon, Therese Duane, Mamta Swaroop, Jonathan Myers, Rebecca Deal, Erik Schadde, Mark Hemmila, Lena Napolitano, Kathleen To, Alex Makupe, Joseph Musowoya, Niels van der Naald, Dayson Kumwenda, Alex Reece-Smith, Kars Otten, Anna Verbeek, Marloes Prins, Alibeth Andres Baquero Suarez, Ruben Balmaceda, Chelsea Deane, Emilio Dijan, Mahmoud Elfiky, Laura Koskenvuo, Aurore Thollot, Bernard Limoges, Carmen Capito, Challine Alexandre, Henri Kotobi, Julien Leroux, Kalitha Pinnagoda, Nicolas Henric, Olivier Azzis, Olivier Rosello, Poddevin Francois, Sara Etienne, Philippe Buisson, Sophian Hmila, Joe-Nat Clegg-Lamptey, Osman Imoro, Owusu Emmanuel Abem, Dimitrios Papageorgiou, Vasiliki Soulou, Sabrina Asturias, Lenin Peña, Donal B O'Connor, Alberto Realis Luc, Alfio Alessandro Russo, Andrea Ruzzenente, Antonio Taddei, Camilla Cona, Corrado Bottini, Giovanni Pascale, Giuseppe Rotunno, Leonardo Solaini, Marco Maria Pascale, Margherita Notarnicola, Mario Corbellino, Michele Sacco, Paolo Ubiali, Roberto Cautiero, Tommaso Bocchetti, Elena Muzio, Vania Guglielmo, Eugenio Morandi, Patrizio Mao, Emilia de Luca, Farah Mahmoud Ali, Justas Žilinskas, Kestutis Strupas, Paulius Kondrotas, Robertas Baltrunas, Juozas Kutkevicius, Povilas Ignatavicius, Choy Ling Tan, Jia Yng Siaw, Sir Young Yam, Ling Wilson, Mohamed Rezal Abdul Aziz, John Bondin, Carmina Diaz Zorrilla, Anass Majbar, Danjuma Sale, Lawal Abdullahi, Olabisi Osagie, Omolara Faboya, Adedeji Fatuga, Agboola Taiwo, Emeka Nwabuoku, Marte Bliksøen, Zain Ali Khan, Jazmin Coronel, Cesar Miranda, Idelso Vasquez, Luis M Helguero-Santin, Jennifer Rickard, Adesina Adedeji, Saleh Alqahtani, Max Rath, Michael Van Niekerk, Modise Zacharia Koto, Roel Matos-Puig, Leif Israelsson, Tobias Schuetz, Mahmut Arif Yuksek, Meric Mericliler, Mehmet Ulusahin, Bernhard Wolf, Cameron Fairfield, Guo Liang Yong, Katharine Whitehurst, Natalie Redgrave, Caroluce K Musyoka, James Olivier, Kathryn Lee, Michael Cox, Muhamed M H Farhan-Alanie, Rory Callan, Chali Chibuye, Tebian Hassanein Ahmed Ali, Syrine Rekhis, Muna Rommaneh, Zi Hao Sam, Thays Brunelli Pugliesi, Gabriel Pardo, Ruth Blanco

## Abstract

**Background:**

Surgical site infection (SSI) is one of the most common infections associated with health care, but its importance as a global health priority is not fully understood. We quantified the burden of SSI after gastrointestinal surgery in countries in all parts of the world.

**Methods:**

This international, prospective, multicentre cohort study included consecutive patients undergoing elective or emergency gastrointestinal resection within 2-week time periods at any health-care facility in any country. Countries with participating centres were stratified into high-income, middle-income, and low-income groups according to the UN's Human Development Index (HDI). Data variables from the GlobalSurg 1 study and other studies that have been found to affect the likelihood of SSI were entered into risk adjustment models. The primary outcome measure was the 30-day SSI incidence (defined by US Centers for Disease Control and Prevention criteria for superficial and deep incisional SSI). Relationships with explanatory variables were examined using Bayesian multilevel logistic regression models. This trial is registered with ClinicalTrials.gov, number NCT02662231.

**Findings:**

Between Jan 4, 2016, and July 31, 2016, 13 265 records were submitted for analysis. 12 539 patients from 343 hospitals in 66 countries were included. 7339 (58·5%) patient were from high-HDI countries (193 hospitals in 30 countries), 3918 (31·2%) patients were from middle-HDI countries (82 hospitals in 18 countries), and 1282 (10·2%) patients were from low-HDI countries (68 hospitals in 18 countries). In total, 1538 (12·3%) patients had SSI within 30 days of surgery. The incidence of SSI varied between countries with high (691 [9·4%] of 7339 patients), middle (549 [14·0%] of 3918 patients), and low (298 [23·2%] of 1282) HDI (p<0·001). The highest SSI incidence in each HDI group was after dirty surgery (102 [17·8%] of 574 patients in high-HDI countries; 74 [31·4%] of 236 patients in middle-HDI countries; 72 [39·8%] of 181 patients in low-HDI countries). Following risk factor adjustment, patients in low-HDI countries were at greatest risk of SSI (adjusted odds ratio 1·60, 95% credible interval 1·05–2·37; p=0·030). 132 (21·6%) of 610 patients with an SSI and a microbiology culture result had an infection that was resistant to the prophylactic antibiotic used. Resistant infections were detected in 49 (16·6%) of 295 patients in high-HDI countries, in 37 (19·8%) of 187 patients in middle-HDI countries, and in 46 (35·9%) of 128 patients in low-HDI countries (p<0·001).

**Interpretation:**

Countries with a low HDI carry a disproportionately greater burden of SSI than countries with a middle or high HDI and might have higher rates of antibiotic resistance. In view of WHO recommendations on SSI prevention that highlight the absence of high-quality interventional research, urgent, pragmatic, randomised trials based in LMICs are needed to assess measures aiming to reduce this preventable complication.

**Funding:**

DFID-MRC-Wellcome Trust Joint Global Health Trial Development Grant, National Institute of Health Research Global Health Research Unit Grant.

## Introduction

Surgical site infection (SSI) is a large health burden for patients and health-care providers. It is the most common postoperative complication and causes pain and suffering to patients.^1^, ^2^ SSI is universally expensive^3^ and could result in catastrophic health expenditure and impoverishment to patients who are required to pay for their own treatment.^4^ In low-income and middle-income countries (LMICs), single-centre retrospective series have suggested that SSI could be the most common infection associated with health care.^1^ However, prospective, standardised, and internationally comparable data on the incidence of SSI and adverse events associated with SSI are lacking.^5^, ^6^, ^7^, ^8^

These knowledge gaps make allocation of resources to tackle SSI in LMICs challenging. The WHO Guideline Development Group recently published 29 preoperative, intraoperative, and postoperative recommendations about SSI prevention.^6^, ^9^, ^10^ These recommendations are welcomed but are necessarily based in large part on data extrapolated from high-income countries and, consequently, might lack validity in resource-limited settings. Strategic planning to tackle SSI has been hindered by a lack of high-quality global data. Microbiological data describing antimicrobial resistance in SSI and information on the likely origin of causative organisms are also needed to help refine prevention strategies and quality-improvement interventions.^6^, ^10^

**Research in context**

**Evidence before this study**

We searched for evidence of multinational research assessing surgical site infection (SSI) after abdominal surgery, focusing on low-income and middle-income countries (LMICs). We searched PubMed, MEDLINE, Google Scholar, and ClinicalTrials.gov for articles published between Jan 1, 1997, and June 1, 2017, with the terms “wound infection” OR “surgical site infection” AND “developing countries” OR “low income” OR “middle income” OR “low and middle income”, without language restrictions. We reviewed related articles, references, and citations of eligible texts. Several low-volume, single-centre studies to characterise SSI in LMICs have been done in the past 20 years, but the research quality is low to medium. These studies were systematically reviewed in 2011 and included 57 studies of abdominal surgery, with reported SSI incidence ranging from 0·4% to 30·9% (between 1·5% and 81·0% for clean–contaminated surgery, 0·5% to 65·5% for contaminated surgery, and 0·2% to 100% for dirty surgery). The methodological quality of individual studies was low and heterogeneity was high, preventing meta-analysis. One multinational study has been done since 2010, and included patients from seven high-income, 17 upper-middle-income, and six lower-middle-income countries. The low observed SSI incidence (4·1%) after abdominal surgery could relate to the passive 30-day follow-up strategy; additional limitations include a lack of data from lowest-income countries and exclusion of children.

**Added value of this study**

We identified the burden and clinical impact of SSI in patients undergoing gastrointestinal surgery in multiple income settings. We used standardised, validated, prospective methodology to provide global, contemporaneous data. SSI is most common after dirty surgery in LMICs. Even after casemix adjustment, patients in LMICs have a disproportionate burden of infection. A large proportion of SSIs are caused by organisms resistant to prophylactic antibiotics with the greatest apparent burden in LMICs.

**Implications of all the available evidence**

The burden of SSI is disproportionately greater on patients and health services in LMICs. Recent WHO recommendations onpreoperative and intraoperative measures for SSI prevention highlight an absence of high-quality evidence. Urgent, pragmatic, randomised trials based in LMICs are needed to assess measures aiming to reduce this preventable complication and associated antibiotic use.

The GlobalSurg Collaborative designed and conducted an international, multicentre, prospective cohort study aimed at closing knowledge gaps in the incidence of SSI in global health settings. The primary aim was to determine variability in SSI rates in high-income, middle-income, and low-income settings.

## Methods

### Study design and participants

This international, multicentre, prospective cohort study used a published protocol^11^ and was done by teams of local investigators who were coordinated by a national lead investigator. Investigators were recruited via the GlobalSurg network, social media, and personal contacts. Any health-care facility in any country treating patients who fulfilled the inclusion criteria could participate. The collaborative network methodology has been described in detail elsewhere.^12^ Ethical and institutional approval was sought and obtained by each contributing institution as per local regulations. A UK National Health Service Research Ethics review considered this study exempt from formal research registration (South East Scotland Research Ethics Service, reference NR/1510AB5). Individual centres obtained their own audit or institutional approval, and ethical approval was obtained in countries where local research ethics committees deemed it a requirement. This study is reported according to the STROBE and SAMPL guidelines.

Investigators included patients from at least one 2-week period that was chosen a priori by the local team. Consecutive sampling of patients undergoing elective or emergency gastrointestinal resection was done during the chosen 2-week period or periods. Consecutive sampling is a common non-probability sampling strategy in which all patients fulfilling the inclusion criteria within a defined time period are enrolled. A 2-week period was chosen to balance sample size requirements and pragmatism for the working clinicians who were enrolling patients and contributing data. The inclusion criteria were based on two considerations. First, the procedures were required to be relevant to the general surgeons who form the collaborative. Second, a reasonable baseline incidence of SSI was required so meaningful comparisons could be made with the predicted cohort size. So-called clean general surgery cases, such as simple hernia repair, were excluded on this basis. There was an absolute requirement for all cases in the chosen period to be included, but no minimum number was set to avoid bias against smaller centres. Gastrointestinal resection was defined as complete transection and removal of a segment of the oesophagus, stomach, small bowel, colon, rectum, appendix, or gallbladder and included formation or reversal of a gastrointestinal stoma. Emergency procedures were defined as unplanned, non-elective operations and included procedures for trauma and reoperation after previous surgery. Open or minimally invasive procedures (eg, laparoscopic or robotic) were eligible. No age restrictions were included. Patients were excluded if the primary indication for surgery was vascular, gynaecological, obstetric, urological, or transplantation because the gastrointestinal tract is not typically opened.

Data variables from the GlobalSurg 1 study^13^ and other studies that have been found to affect the likelihood of SSI were entered into risk adjustment models. Patient variables included age, sex, physical status according to the American Society of Anesthesiologists classification system, existence of immune suppression (eg, HIV status, active malarial infection, diabetes, use of steroid therapies, chemotherapy or other immunosuppressive drugs), and smoking status. Disease-related variables included diagnostic category, timing of surgery (elective *vs* emergency), use of the WHO surgical safety checklist,^14^ use of laparoscopy, use of epidural anaesthesia, use of prophylactic antibiotics, and intraoperative contamination. Contamination level^2^, ^15^ was defined by the operating surgeon as clean (an incision in which no inflammation is encountered in a surgical procedure, without a break in sterile technique, and during which the respiratory, alimentary, and genitourinary tracts are not entered; inclusion criteria for this study excluded this group), clean–contaminated (an incision through which the respiratory, alimentary, or genitourinary tract is entered under controlled conditions but with no contamination encountered), contaminated (an incision undertaken during an operation in which there is a major break in sterile technique or gross spillage from the gastrointestinal tract, or an incision in which acute, non-purulent inflammation is encountered; open traumatic wounds more than 12–24 h old also fall into this category), or dirty (an incision undertaken during an operation in which the viscera are perforated or when acute inflammation with pus is encountered during the operation [eg, emergency surgery for faecal peritonitis], and for traumatic wounds where treatment is delayed, and faecal contamination or devitalised tissue is present).

Data were collected on the incidence and length of antimicrobial treatment before and after surgery. A pragmatic view was taken in the use of local protocols and techniques for collecting and processing microbiological specimens. Antimicrobial resistance was defined as resistance in the species presumed to be pathological to the antimicrobial used for prophylaxis. To aid in the communication of findings, organisms were broadly categorised as bowel-derived if cultures contained only Gram-negative bacilli, *Enterococcus* species, or anaerobic organisms, as skin-derived if cultures only contained skin-derived organisms such as *Staphylococcus* species, and as of mixed origin if cultures contained both bowel-derived and skin-derived cultures.

Data variables were selected to be objective, standardised, easily transcribed, and internationally relevant to maximise record completion and accuracy. Local investigators uploaded records to a secure online website, provided using the Research Electronic Data Capture (REDCap) system.^16^ The lead investigator at each site checked the accuracy of all cases before data submission. The submitted data were then checked centrally and when missing data were identified, the local lead investigator was contacted and asked to complete the record. Once vetted, the record was accepted into the dataset for analysis. Records that were vetted but remained incomplete were included in the patient flowchart but excluded from analysis.

Data validation was done in three parts across a representative sample of centres according to a pre-specified protocol (appendix). First, centres self-reported the key processes used to identify and follow up patients. Second, independent validators (ie, doctors, nurses, or medical students who were not part of the recruiting teams) quantitatively reported case ascertainment and sampled data accuracy. Third, teams were interviewed to qualitatively assess collaborator engagement and data collection processes.

### Outcomes

The primary outcome measure was the 30-day SSI incidence, defined using the US Centers for Disease Control and Prevention criteria for superficial and deep incisional SSI.^2^ These criteria require the patient to have at least one of the following: (1) purulent drainage from the superficial or deep (fascia or muscle) incision but not from within the organ or space component of the surgical site; (2) pain or tenderness, localised swelling, redness, heat, or fever, or several of these symptoms, and the incision is opened deliberately or spontaneously dehisces; or (3) abscess within the wound (clinically or radiologically detected).

Organ space infections were recorded separately and defined as intra-abdominal or pelvic infections detected clinically or symptomatically, radiologically, or intra-operatively. A mandatory online SSI training module was completed by all collaborators before data collection.

The secondary outcome measures were designed to describe the clinical effect of SSI and included: (1) 30-day postoperative mortality, defined as death any time after skin closure until 30 days after surgery;^17^ (2) prevalence in perioperative antibiotic administration; (3) 30-day postoperative reintervention incidence (operative, radio-logical, or endoscopic reintervention any time after skin closure until 30 days after surgery); (4) the prevalence of antimicrobial resistance for SSI (microbiological culture of wound swabs from site of SSI done according to local protocols, with a pragmatic definition of antimicrobial resistance defined a priori as resistance to the anti-microbial drug used for prophylaxis for that procedure in that particular patient); and (5) in-hospital SSI incidence (patients were reviewed for SSI during their stay and at the time of hospital discharge); and (6) overall 30-day SSI incidence (patients were assessed at 30 days to determine whether an SSI had occurred; follow-up was done in person, by telephone, or by review of medical/readmission records, dependent on local practices).

### Statistical analysis

As described in the protocol,^11^ consideration was given to the sample size needed to compare HDI groups. This was approximated because data describing SSI incidence internationally are lacking. Taken with data from the GlobalSurg 1 study,^13^ for a baseline SSI incidence of 15%, 550 patients per group (1350 patients in total after accounting for potential missing data and loss to follow-up) would allow for a 6·5 percentage point difference to be detected with a power of 80% at an α significance level of 0·05.

Variation between different international health settings was assessed by stratifying countries with participating centres into tertiles according to the Human Development Index (HDI). The HDI is the UN's composite statistic of life expectancy, education, and income indices. Differences between HDI tertiles were tested with the Pearson χ^2^ test for categorical variables and with the Kruskal-Wallis test for continuous variables. Bayesian multilevel logistic regression models were constructed to account for casemix (differing patient, disease, and operative characteristics), as previously described.^18^ Briefly, non-informative priors were used with sensitivity analyses done on alternative priors and different chain initiation points or chain lengths. Models were constructed using the following principles: (1) variables associated with outcome measures in previous studies were accounted for; (2) demographic variables were included in model exploration; (3) population stratification by hospital and country of residence was incorporated as random effects with constrained gradients; (4) all first-order interactions were checked and included in final models if found to be influential; (5) final model selection was done using a criterion-based approach by minimising the widely applicable information criterion (WAIC) and discrimination determined using the c-statistic (area under the receiver operator curve). Model coefficients are presented as odds ratio (OR) and 95% credible intervals (CI; analogous to confidence intervals in frequentist statistics, but philosophically distinct). In a further analysis, a restricted cubic spline transformation was applied to the continuous representation of the HDI to account for potential non-linearity (three knots distributed equally across the range of HDI rank). This was substituted into the final multilevel model (generalised additive model) and posterior predictions were made for specified covariate levels with 95% CI determined. All analyses were done using the R Foundation Statistical Program version 3.1.1 and Stan A C++ Library for Probability and Sampling version 2.10.0. This trial is registered with ClinicalTrials.gov, number NCT02662231.

### Data sharing

The dataset can be explored using an online visualisation application at http://ssi.globalsurg.org.

### Role of the funding source

The funder of the study had no role in study design, data collection, data analysis, data interpretation, or writing of the report. The corresponding author had full access to all the data in the study and had final responsibility for the decision to submit for publication.

## Results

**Figure 1:**
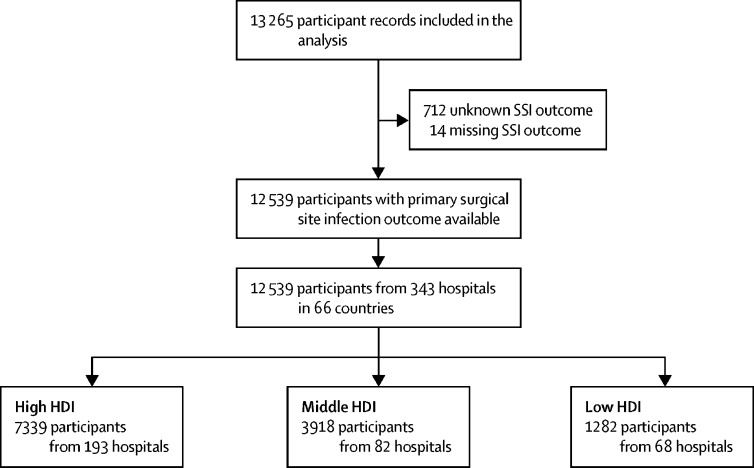
Patient flowchart SSI=surgical site infection. HDI=Human Development Index.

Between Jan 4, 2016, and July 31, 2016, 13 265 patient records were submitted for analysis. 726 (5·5%) records remained incomplete after quality control, leaving 12 539 records for the final analysis (figure 1). These patients were from 343 centres across 66 countries (15 countries in Africa, 16 countries in Asia, 22 countries in Europe, eight countries in North America, one country in Oceania, and four countries in South America; table 1). 7339 (58·5%) patients were from countries with high HDI, 3918 (31·2%) patients were from countries with middle HDI, and 1282 (10·2%) patients were from countries with low HDI. 1291 (10·3%) patients were children (aged 16 years or younger). Missing data were uncommon (appendix p 12), and no patterns were seen when comparing included and missing data (appendix pp 13, 14).

**Table 1:** Patient and operative characteristics by human development index (HDI) rank

		High HDI (n=7339)*	**Middle HDI (n=3918)**†	**Low HDI (n=1282)**‡	**Total (n=12 539)**§	**p value**
Mean age (SD), years		48·7 (21·5)	37·3 (18·5)	32·4 (19·1)	43·5 (21·3)	<0·001
Sex						
	Male	3248 (44·3%)	1508 (38·5%)	678 (52·9%)	5434 (43·3%)	<0·001
	Female	3683 (50·2%)	2215 (56·5%)	562 (43·8%)	6460 (51·5%)	··
	Missing	408 (5·6%)	195 (5·0%)	42 (3·3%)	645 (5·1%)	··
ASA						
	I	2498 (34·0%)	2299 (58·7%)	687 (53·6%)	5484 (43·7%)	<0·001
	II	3191 (43·5%)	1106 (28·2%)	409 (31·9%)	4706 (37·5%)	··
	III+	1543 (21·0%)	293 (7·5%)	178 (13·9%)	2014 (16·1%)	··
	Unknown	107 (1·5%)	220 (5·6%)	7 (0·5%)	334 (2·7%)	··
	Missing	0	0	1 (0·1%)	1 (<0·1%)	··
HIV						
	No	6773 (92·3%)	3573 (91·2%)	1097 (85·6%)	11 443 (91·3%)	<0·001
	Yes	13 (0·2%)	39 (1·0%)	5 (0·4%)	57 (0·5%)	··
	Unknown	553 (7·5%)	306 (7·8%)	179 (14·0%)	1038 (8·3%)	··
	Missing	0	0	1 (0·1%)	1 (<0·1%)	··
Malaria						
	No	7243 (98·7%)	3845 (98·1%)	1157 (90·2%)	12 245 (97·7%)	<0·001
	Yes	8 (0·1%)	3 (0·1%)	10 (0·8%)	21 (0·2%)	··
	Unknown	87 (1·2%)	69 (1·8%)	115 (9·0%)	271 (2·2%)	··
	Missing	1 (<0·1%)	1 (<0·1%)	0	2 (<0·1%)	··
Diabetes						
	No	6484 (88·3%)	3564 (91·0%)	1184 (92·4%)	11 232 (89·6%)	<0·001
	Yes	745 (10·2%)	309 (7·9%)	73 (5·7%)	1127 (9·0%)	··
	Unknown	110 (1·5%)	44 (1·1%)	25 (2·0%)	179 (1·4%)	··
	Missing	0	1 (<0·1%)	0	1 (<0·1%)	··
Immunosuppressive medication						
	No	6893 (93·9%)	3789 (96·7%)	1243 (97·0%)	11 925 (95·1%)	<0·001
	Yes	446 (6·1%)	129 (3·3%)	39 (3·0%)	614 (4·9%)	··
Current smoker						
	No	6190 (84·3%)	3353 (85·6%)	1170 (91·3%)	10 713 (85·4%)	<0·001
	Yes	1149 (15·7%)	565 (14·4%)	112 (8·7%)	1826 (14·6%)	··
Pathology						
	Appendicitis	2061 (28·1%)	1516 (38·7%)	502 (39·2%)	4079 (32·5%)	<0·001
	Gallstone disease	2505 (34·1%)	1493 (38·1%)	290 (22·6%)	4288 (34·2%)	··
	Malignancy	1510 (20·6%)	287 (7·3%)	104 (8·1%)	1901 (15·2%)	··
	Benign foregut	446 (6·1%)	220 (5·6%)	49 (3·8%)	715 (5·7%)	··
	Benign midgut or hindgut	570 (7·8%)	150 (3·8%)	121 (9·4%)	841 (6·7%)	··
	Infection	46 (0·6%)	41 (1·0%)	63 (4·9%)	150 (1·2%)	··
	Congenital	47 (0·6%)	49 (1·3%)	85 (6·6%)	181 (1·4%)	··
	Trauma or injury	18 (0·2%)	47 (1·2%)	45 (3·5%)	110 (0·9%)	··
	Complication of previous procedure	67 (0·9%)	23 (0·6%)	14 (1·1%)	104 (0·8%)	··
	Other	33 (0·4%)	9 (0·2%)	6 (0·5%)	48 (0·4%)	··
	No disease	36 (0·5%)	80 (2·0%)	3 (0·2%)	119 (0·9%)	··
	Missing	0	3 (0·1%)	0	3 (<0·1%)	··
Procedure start-time						
	0800 h to 1759 h	5788 (78·9%)	2753 (70·3%)	865 (67·5%)	9406 (75·0%)	<0·001
	1800 h to 2159 h	724 (9·9%)	381 (9·7%)	180 (14·0%)	1285 (10·2%)	··
	2200 h to 0759 h	821 (11·2%)	782 (20·0%)	237 (18·5%)	1840 (14·7%)	··
	Missing	6 (0·1%)	2 (0·1%)	0 (0·0%)	8 (0·1%)	··
Admission to procedure time, h						
	<6	2291 (31·2%)	1052 (26·9%)	308 (24·0%)	3651 (29·1%)	<0·001
	6–11	722 (9·8%)	366 (9·3%)	128 (10·0%)	1216 (9·7%)	··
	12–23	1364 (18·6%)	675 (17·2%)	217 (16·9%)	2256 (18·0%)	··
	24–47	1330 (18·1%)	552 (14·1%)	230 (17·9%)	2112 (16·8%)	··
	≥48	1358 (18·5%)	1033 (26·4%)	338 (26·4%)	2729 (21·8%)	··
	Missing	274 (3·7%)	240 (6·1%)	61 (4·8%)	575 (4·6%)	··
Urgency						
	Elective	3941 (53·7%)	1997 (51·0%)	483 (37·7%)	6421 (51·2%)	<0·001
	Emergency	3397 (46·3%)	1921 (49·0%)	799 (62·3%)	6117 (48·8%)	··
	Missing	1 (<0·1%)	0	0	1 (<0·1%)	··
Operative approach						
	Open	2679 (36·5%)	2153 (55·0%)	1055 (82·3%)	5887 (46·9%)	<0·001
	Laparoscopic	4660 (63·5%)	1765 (45·0%)	227 (17·7%)	6652 (53·1%)	··
Epidural						
	No	6554 (89·3%)	3666 (93·6%)	1230 (95·9%)	11 450 (91·3%)	<0·001
	Yes	646 (8·8%)	210 (5·4%)	48 (3·7%)	904 (7·2%)	··
	Unknown	139 (1·9%)	42 (1·1%)	4 (0·3%)	185 (1·5%)	··
Antibiotic: pre-procedural or prophylactic						
	No	848 (11·6%)	472 (12·0%)	50 (3·9%)	1370 (10·9%)	<0·001
	Yes	6446 (87·8%)	3392 (86·6%)	1224 (95·5%)	11062 (88·2%)	··
	Missing	45 (0·6%)	54 (1·4%)	8 (0·6%)	107 (0·9%)	··
Intraoperative contamination						
	Clean–contaminated	5918 (80·6%)	3126 (79·8%)	878 (68·5%)	9922 (79·1%)	<0·001
	Contaminated	779 (10·6%)	542 (13·8%)	219 (17·1%)	1540 (12·3%)	··
	Dirty	574 (7·8%)	236 (6·0%)	181 (14·1%)	991 (7·9%)	··
	Missing	68 (0·9%)	14 (0·4%)	4 (0·3%)	86 (0·7%)	··
Safety checklist used						
	No, not available	837 (11·4%)	1114 (28·4%)	308 (24·0%)	2259 (18·0%)	<0·001
	No, but available	238 (3·2%)	690 (17·6%)	363 (28·3%)	1291 (10·3%)	··
	Yes	6194 (84·4%)	2049 (52·3%)	600 (46·8%)	8843 (70·5%)	··
	Unknown	69 (0·9%)	65 (1·7%)	11 (0·9%)	145 (1·2%)	··
	Missing	1 (<0·1%)	0	0	1 (<0·1%)	··

Numbers are n (%), unless otherwise indicated. All tests are Pearson's χ^2^ test, except for the comparison of mean age, where a Kruskall-Wallis test has been applied. ASA=American Society of Anesthesiologists classification grade.

* Included 30 countries and 193 hospitals.

† Included 18 countries and 82 hospitals.

‡ Included 18 countries and 68 hospitals.

§ Included 66 countries and 343 hospitals.

The most common operations were cholecystectomy (4412 [35·2%] of 12 539 patients) and appendicectomy (4179 [33·3%]; appendix p 1). 6117 (48·8%) patients had emergency surgery, 5887 (46·9%) patients had an open approach, and a surgical safety checklist was used before 8843 (70·5%) cases (table 1). Overall, 9922 (79·1%) operations were clean–contaminated, 1540 (12·3%) operations were contaminated, and 991 (7·9%) operations were dirty.

**Figure 2:**
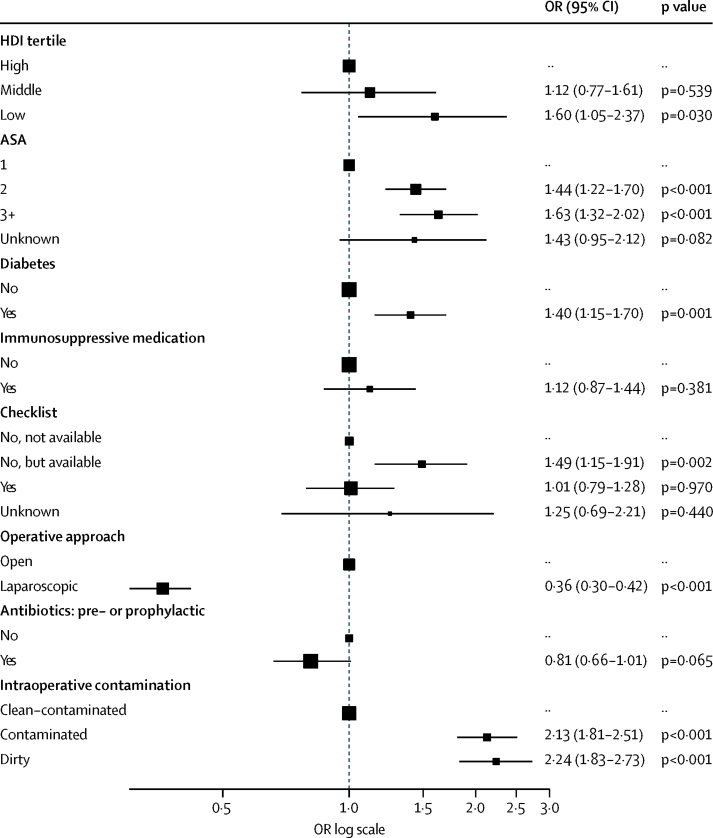
Multilevel model for factors associated with surgical site infection Full model includes HDI tertile, age, American Society of Anesthesiologists (ASA) classification grade, diabetes status, immunosuppressive medication treatment, current smoker, pathology, operative approach, antibiotic use before surgery, intraoperative contamination, and WHO checklist used. Error bars are 95% credible interval. Full data are in the appendix (p 4). OR=odds ratio. HDI=Human Development Index. CI=credible interval.

**Figure 3:**
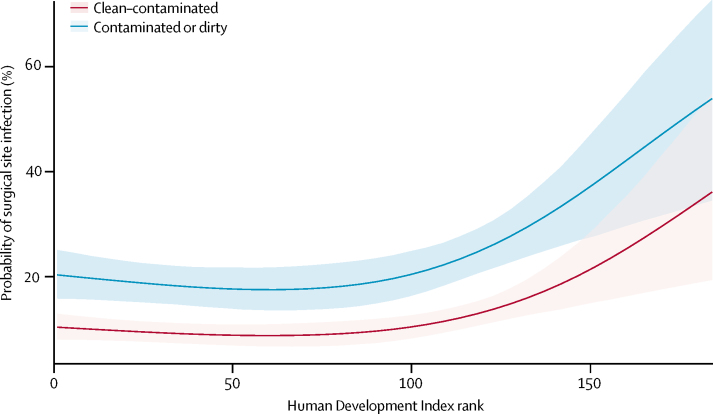
Probability of surgical site infection (SSI) by human development index (HDI) rank Adjusted predicted probability of SSI across HDI rank by intraoperative contamination. In the most developed countries (rank 1), patients had a low probability of SSI. At rank 100, the probability of SSI increases linearly through the least developed countries. This absolute difference between clean–contaminated and contaminated or dirty surgery is shown, with no interaction between HDI and intraoperative contamination found. Shaded area is the credible interval.

1538 (12·3%) patients had SSI within 30 days of surgery, and 842 (6·7%) had SSI before discharge from hospital (appendix p 5). The unadjusted SSI incidence varied between countries with high HDI (691 [9·4%] of 7339 patients), middle HDI (549 [14·0%] of 3918 patients) and low HDI (298 [23·2%] of 1282 patients). Intraoperative contamination was more likely to be classed as dirty in countries with low HDI (181 [14·1%] of 1282 patients) than in countries with middle HDI (236 [6·0%] of 3918 patients) or high HDI (574 [7·8%] of 7339; table 1). SSI rates increased significantly with dirty surgery compared with clean–contaminated surgery; however, there was no significant interaction for SSI between HDI and intraoperative contamination (appendix pp 3, 4). After multivariable adjustment for confounders (including contamination), a significantly higher SSI rate was seen in countries with low HDI (adjusted OR 1·60, 95% CI 1·05–2·37; p=0·030) but not in middle-HDI settings (1·12, 0·77–1·61; p=0·539) compared with high-HDI countries (figure 2; appendix p 4). When adjusted for patient and hospital factors, SSI increased markedly at the threshold between countries with middle and low HDI (rank 100; figure 3). The increase was observed for both clean–contaminated and dirty surgery because there was no significant interaction between contamination and HDI, suggesting that HDI is an independent risk factor for SSI, irrespective of intraoperative contamination.

235 (1·9%) patients died within 30 days of surgery, but mortality varied between countries with high HDI (110 [1·5%] of 7339 patients), middle HDI (64 [1·6%] of 3918 patients), and low HDI (61 [4·8%] of 1282 patients; appendix p 5). Patients with SSI were more likely than patients without SSI to die, to have a reintervention, to have an organ space infection, or to have another health-care-associated infection (table 2). The median length of hospital stay was three times longer for patients with an SSI than for patients without (median 7·0 days [IQR 11·0] *vs* 2·0 days [4·0]; p<0·001).

**Table 2:** Associations between surgical site infection (SSI) and other outcomes

		**No SSI (n=11 001)**	**SSI (n=1538)**	**p value**
30-day mortality				
	Alive	10 665 (96·9%)	1438 (93·5%)	<0·001
	Dead	162 (1·5%)	73 (4·7%)	··
	Missing	174 (1·6%)	27 (1·8%)	··
30-day reintervention				
	No	10 674 (97·0%)	1202 (78·2%)	<0·001
	Yes	235 (2·1%)	316 (20·5%)	··
	Missing	92 (0·8%)	20 (1·3%)	··
Organ space infection (abscess)				
	No	10 759 (97·8%)	1229 (79·9%)	<0·001
	Yes	146 (1·3%)	276 (17·9%)	··
	Missing	96 (0·9%)	33 (2·1%)	··
Other health-care-associated infection				
	No	10 546 (95·9%)	1292 (84·0%)	<0·001
	Yes	388 (3·5%)	214 (13·9%)	··
	Missing	67 (0·6%)	32 (2·1%)	··
Median length of stay (IQR)		2·0 (4·0)	7·0 (11·0)	<0·001*

Numbers are n (%), unless otherwise indicated.

* All tests are χ^2^ tests, except when indicated by, where a Kruskall-Wallis test has been applied.

Patients in LMICs were more likely to receive presurgery antibiotic courses than patients in high-HDI settings (appendix p 6). Prophylactic antibiotic administration was generally high (10 225 [81·5%] of 12 539 patients), with slight variation between HDI groups. Overall, administration of preoperative or prophylactic antibiotics, or both, was higher in groups with low HDI (1224 [95·5%] of 1282) than in countries with middle HDI (3392 [86·6%] of 3918 patients) and high HDI (6446 of 87·8%] of 7339 patients; p<0·001).

Patients in LMICs were more likely to receive postoperative antibiotics than those in high-HDI countries (3376 [46·0%] of 7339 patients in high-HDI countries *vs* 3135 [80·0%] of 3918 patients in middle-HDI countries *vs* 1098 [85·6%] of 1282 patients in low-HDI countries; p<0·001; appendix p 7). The increased tendency to use antibiotics after surgery in low-HDI countries persisted despite adjustment for confounding factors (adjusted OR 4·37, 95% CI 1·65–11·85, p=0·002), including contamination of surgery (appendix pp 8, 9). Courses of postoperative antibiotics were longer in patients in LMICs than in high-income countries, with the number of patients receiving antibiotics for 5 days or more increasing from countries with high HDI to low HDI (1830 [24·9%] of 7339 patients in high-HDI countries *vs* 1837 [46·9%] of 3918 patients in middle-HDI countries *vs* 650 [50·7%] of 1282 patients in low-HDI countries; p<0·001; appendix p 7).

A microbiological wound culture was available for 610 (39·7%) of 1538 patients with an SSI (table 3). A summary and full lists of causative organisms are available in the appendix (pp 10, 11). 301 [63·8%] of 472 patients had bowel-derived infections, 97 (20·6%) patients had skin-derived infections, and 53 (11·2%) patients had infections of mixed origin. Organisms with resistance to the actual prophylactic antibiotic used were isolated from 132 (21·6%) of the 610 patients with SSI who had a wound culture (table 3). The prevalence of resistance varied between countries with high, middle, and low HDI (table 3).

**Table 3:** Sensitivity of organism by Human Development Index (HDI) from patients with a surgical site infection who had a wound swab taken

	**High HDI (n=295)**	**Middle HDI (n=187)**	**Low HDI (n=128)**	**Total (n=610)**	**p value**
Antibiotic not used	27 (9·2%)	6 (3·2%)	0 (0·0%)	33 (5·4%)	<0·001
Sensitive to antibiotic	92 (31·2%)	56 (29·9%)	40 (31·2%)	188 (30·8%)	··
Resistant to antibiotic	49 (16·6%)	37 (19·8%)	46 (35·9%)	132 (21·6%)	··
Sensitivity not available	127 (43·1%)	88 (47·1%)	42 (32·8%)	257 (42·1%)	··

Numbers are n (%), unless otherwise indicated. All tests are χ^2^ tests.

Patients were identified for inclusion predominately using theatre logbooks or computer systems (5771 [78·6%] of 7339 patients in high-HDI countries; 2349 [60·0%] of 3918 patients in middle-HDI countries; 759 [59·2%] of 1282 patients in low-HDI countries) and operating lists (1318 [18·0%] of 7339 patients in high-HDI countries; 823 [21·0%] of 3918 patients in middle-HDI countries; 278 [21·7%] of 1282 patients in low-HDI countries; appendix p 16). Many patients across HDI strata were followed up by telephone (2708 [36·9%] of 7339 patients in high-HDI countries; 2582 [65·9%] of 3918 patients in middle-HDI countries; 483 [37·7%] of 1282 patients in low-HDI countries; appendix p 17). Validators identified 1476 cases that fulfilled inclusion criteria, and 1378 (93%) cases were ascertained (appendix p 18). Accuracy was high for the validated continuous predictor (Pearson's correlation coefficient 0·99; appendix p 20), categorical predictors (Cohen's κ coefficients >0·90; appendix p 21), and mortality (κ 0·91). The agreement for 30-day reintervention was lower (κ 0·65).

## Discussion

We identified both the burden and clinical effect of SSI on patients undergoing gastrointestinal surgery in many parts of the world. SSI affected 12·3% of patients worldwide, and the incidence increased across HDI groups, reaching 39·8% of patients undergoing dirty surgery in low-HDI settings. The incidence of SSI remained higher in low-HDI countries than in middle-HDI or high-HDI countries, despite adjustment for factors describing patients, diseases (including contamination), procedures, safety, and hospitals. Length of hospital stay was three times longer for patients affected by SSI than for patients with no SSI. Delayed return to work or school carries a societal burden, which is likely to be greater in LMICs.

These findings begin to characterise the relationship between SSI and global antimicrobial resistance. Where microbiological cultures were available, SSIs were more likely to be caused by bowel-derived organisms. Large amounts of antibiotics were consumed to prevent and treat SSI, yet in 21·6% of cases with a positive culture, the causative microorganism was resistant to the prophylactic antibiotics that had been administered. The prevalence of antimicrobial resistance increased to one of three isolates in low-HDI countries. Postoperative courses of antibiotics were longest for patients in low-HDI countries, and this was not explained by casemix. Although there is randomised evidence that short postoperative antibiotic courses are as safe as long antibiotic courses, this evidence was not derived in LMICs, and caution is needed before changing practice.^19^ The high prevalence of SSIs that were resistant to the initial prophylactic antibiotic illustrates a potentially important area for improvement worldwide. Complete microbiological analysis of all SSIs was not possible within this observational study, so the problem might be even larger that estimated here.

The focus in global surgery to date has been directed towards mortality. The 30-day mortality in this study was similar to that in the GlobalSurg 1 study (1·9% and 1·6% respectively).^13^ This generally low mortality highlights the importance of studying more common outcomes such as SSI across health systems, given the impact on patients. We found an association between SSI and death, with a three-fold increase from 1·5% in patients without SSI to 4·7% in patients with SSI within this study. This is an association, and no causal link can be made with these data; it is likely that patients died with an SSI rather than from an SSI. Since SSI was also associated with deep organ space infection and other health-care-associated infections, this supports its use as a severity marker of illness.

Interest in the use of surgical safety checklists has increased in the past 5 years, and they are now part of clinical routine in many surgical units. In this study, the failure to use an available surgical safety checklist was associated with a high SSI rate. This association was not explained by an omission of prophylactic antibiotics, nor was it particular to emergency surgery, when haste might improperly trump safety measures. The scientific literature describing checklists and SSI is contrasting and includes a recent systematic review of 14 studies.^14^ The data in this systematic review showed a decrease in SSI with checklist use (range within individual studies from 3·2% to 10·2% absolute risk reduction). The GlobalSurg studies provide novel checklist data from LMIC settings. The explanation for the observed effect is unclear but probably describes a broader attitude to safety in hospital systems that require further investigation.

A major strength of this study is its provision of prospective patient-level SSI data from a wide breadth of settings around the world. In particular, outcome assessment was standardised and training provided through our online tool. Several small and generally single-centre studies have been done in the past 20 years in attempts to characterise SSI in LMICs. These were systematically reviewed in a 2010 study^1^ that included 57 reports focusing on SSI. General methodological quality was low and heterogeneity was high, with reported SSI rates varying from 0·4% to 30·9%. Since then, SSI outcomes from several single-centre and national multicentre studies in LMICs have been published.^20^, ^21^, ^22^, ^23^, ^24^ The lower than expected rates emphasise the difficulty in robustly determining SSI, which, together with the between-study variability, make international comparisons difficult. The present study contributes to closing this knowledge gap and allows meaningful comparison from multiple income settings with accurate casemix adjustment and standardised training in outcome assessment. Reliability was increased through the vetting of incomplete records and was demonstrated in a parallel validation study.

A major limitation of this study was the inability to follow up every patient 30 days after surgery. SSI detection within randomised trials is higher when proactively followed up as a primary endpoint than when followed up as a secondary outcome.^25^ Within our study, collaborators were trained and encouraged to directly determine 30-day outcomes whenever possible. Overall, this was successful; however, complete, in-person, 30-day follow-up for thousands of patients would not have been possible, particularly in resource-limited settings. Nevertheless, we did assess SSI as a primary endpoint, used a mandatory training package, and did a sensitivity analysis using in-hospital SSI rates. The variation in incidence of SSI before discharge from hospital and within 30 days was similar between countries of high, middle, and low HDI. Since these incidence data are already comparable to those from high-quality randomised trials, this provides some measure of validity.^26^ Other limitations apply. First, with respect to microbiological analysis, we did not standardise specimen collection, laboratory assessment, techniques, or definitions. A pragmatic view was taken to use local protocols and techniques for collecting and processing specimens and for determining antimicrobial resistance. These measures were therefore recognised in advance as being an exploratory analysis to describe the prevalence of organisms with antimicrobial resistance against the particular prophylactic antibiotic administered. Second, although we did validation, there is still the potential for missed cases or inaccurate data.^13^, ^27^, ^28^ The large number of patients, a prospective protocol, and the use of local coordinators might have minimised the potential bias.

Reducing SSI will contribute to ensuring safe and essential surgery around the world.^29^ Costs to patients in LMICs in terms of expenditure and time off work have not been measured but are probably considerable. The costs of preventive measures might be offset by the realised cost-savings. WHO has published recommendations to help reduce the incidence of SSI that include global perspectives relevant to LMICs.^9^ Despite inclusion of strongly graded recommendations, none of these could be based on high-quality evidence, which is lacking in support of most interventions. Virtually none of the existing evidence is derived from LMICs, leading to uncertainty about future performance of these measures.^8^ SSI research is complex, and bundles of measures have been seen to paradoxically increase SSI incidence.^30^ Implementation therefore necessitates careful consideration and meticulous attention to longer-term evaluation. In resource-limited settings, the development of robust policy will remain difficult without high-quality evidence. Our findings provide the rationale to plan, fund, and perform high-quality surgical research that can effect change in health policy. There are no multicentre, multi-country randomised trials on SSI prevention in LMICs at a time when efforts to combat SSI should be informed by high-quality research derived in these settings.^8^

Correspondence to: Dr Ewen M Harrison, NIHR Unit on Global Surgery (Universities of Birmingham, Edinburgh and Warwick), University of Edinburgh, Clinical Surgery, Royal Infirmary of Edinburgh, Edinburgh EH16 4SA, UK **ewen.harrison@ed.ac.uk** or **enquiry@globalsurg.org**
