# Supplementary material for: Surgical site infection after gastrointestinal surgery in high-income, middle-income, and low-income countries: a prospective, international, multicentre cohort study
Source: Lancet Infect Dis. 2018 May;18(5):516–25. doi: 10.1016/S1473-3099(18)30101-4 (PMC5910057; doi:10.1016/S1473-3099(18)30101-4)
Supplement: Supplementary appendix [file mmc1.pdf]

THE LANCET Infectious Diseases

Supplementary webappendix

This webappendix formed part of the original submission and has been peer reviewed. We post it as supplied by the authors.

Supplement to: GlobalSurg Collaborative. Surgical site infection after gastrointestinal surgery in high-income, middle-income, and low-income countries: a prospective, international, multicentre cohort study. *Lancet Infect Dis* 2018; published online Feb 13. http://dx.doi.org/10.1016/S1473-3099(18)30101-4.

SUPPLEMENTARY MATERIAL

Table 1s – Procedures by human development index tertile

OPCS code and procedure	High (n = 7,339)	Middle (n = 3,918)	Low (n = 1,282)	Total (n = 12,539)
J18 Gallbladder: Excision of gall bladder	2577 (35.1)	1540 (39.3)	295 (23.0)	4412 (35.2)
H01 Appendix: Emergency excision of appendix	2106 (28.7)	1564 (39.9)	509 (39.7)	4179 (33.3)
G58 Small bowel: Excision of small bowel	296 (4.0)	100 (2.6)	104 (8.1)	500 (4.0)
G28 Stomach: Partial excision of stomach	300 (4.1)	174 (4.4)	25 (2.0)	499 (4.0)
H07 Colon: Excision of right hemicolon	410 (5.6)	39 (1.0)	34 (2.7)	483 (3.9)
H10 Colon: Excision of sigmoid colon	320 (4.4)	75 (1.9)	17 (1.3)	412 (3.3)
H33 Rectum: Excision of rectum	346 (4.7)	36 (0.9)	13 (1.0)	395 (3.2)
H15 Colon: Formation of any colonic stoma	126 (1.7)	46 (1.2)	62 (4.8)	234 (1.9)
G67 Small bowel: Other open operations on small bowel	94 (1.3)	46 (1.2)	35 (2.7)	175 (1.4)
H09 Colon: Excision of left hemicolon	113 (1.5)	36 (0.9)	8 (0.6)	157 (1.3)
H11 Colon: Other excision of colon	90 (1.2)	51 (1.3)	11 (0.9)	152 (1.2)
G74 Small bowel: Formation of ileostomy	60 (0.8)	30 (0.8)	43 (3.4)	133 (1.1)
G32 Stomach: Connection of stomach to jejunum	79 (1.1)	20 (0.5)	24 (1.9)	123 (1.0)
H19 Colon: Other open operations on colon	51 (0.7)	39 (1.0)	23 (1.8)	113 (0.9)
H05 Colon: Total excision of colon	78 (1.1)	22 (0.6)	3 (0.2)	103 (0.8)
G27 Stomach: Total excision of stomach	62 (0.8)	25 (0.6)	5 (0.4)	92 (0.7)
H06 Colon: Extended excision of right hemicolon	61 (0.8)	5 (0.1)	16 (1.2)	82 (0.7)
H46 Rectum: Other open operations on rectum	25 (0.3)	20 (0.5)	28 (2.2)	73 (0.6)
G03 Oesophagus: Excision of oesophagus	55 (0.7)	2 (0.1)	5 (0.4)	62 (0.5)
J23 Gallbladder: Other open operations on gall bladder	33 (0.4)	23 (0.6)	5 (0.4)	61 (0.5)
H04 Colon: Total excision of colon and rectum	24 (0.3)	6 (0.2)	0 (0.0)	30 (0.2)
H08 Colon: Excision of transverse colon	17 (0.2)	8 (0.2)	2 (0.2)	27 (0.2)
G70 Small bowel: Excision of Meckel diverticulum	12 (0.2)	5 (0.1)	7 (0.5)	24 (0.2)
T281 Abdomen: Closure of gastroschisis/exomphalos	3 (0.0)	6 (0.2)	3 (0.2)	12 (0.1)
G536 Duodenum: Correction of malrotation	1 (0.0)	0 (0.0)	5 (0.4)	6 (0.0)

Numbers are n (%), unless otherwise indicated. OPCS, OPCS Classification of Interventions and Procedures System.

Table 2s – Pathology by human development index tertile

ICD-10 code and pathology	High (n = 7,339)	Middle (n = 3,918)	Low (n = 1,282)	Total (n = 12,539)
K80 Cholelithiasis / cholecystitis (gallstones)	2505 (34.1)	1493 (38.1)	290 (22.6)	4288 (34.2)
K35 Appendicitis	2061 (28.1)	1516 (38.7)	502 (39.2)	4079 (32.5)
C26 Neoplasm: any malignant (cancer)	1510 (20.6)	287 (7.3)	104 (8.1)	1901 (15.2)
E66 Obesity	227 (3.1)	77 (2.0)	2 (0.2)	306 (2.4)
D13 Neoplasm: any benign	86 (1.2)	93 (2.4)	16 (1.2)	195 (1.6)
K565 Intestinal obstruction: Adhesions	75 (1.0)	47 (1.2)	34 (2.7)	156 (1.2)
K57 Diverticular disease	138 (1.9)	9 (0.2)	4 (0.3)	151 (1.2)
Q43 Congenital: Other	31 (0.4)	34 (0.9)	65 (5.1)	130 (1.0)
00 No disease identified	36 (0.5)	80 (2.0)	3 (0.2)	119 (0.9)
K50 Colitis/gastroenteritis: Crohn’s disease	99 (1.3)	7 (0.2)	1 (0.1)	107 (0.9)
K562 Intestinal obstruction: Volvulus	45 (0.6)	27 (0.7)	33 (2.6)	105 (0.8)
Y83 Complication of previous surgical operation / procedure	67 (0.9)	23 (0.6)	14 (1.1)	104 (0.8)
K46 Hernia: any abdominal hernia	45 (0.6)	30 (0.8)	22 (1.7)	97 (0.8)
A49 Infection: other	32 (0.4)	32 (0.8)	30 (2.3)	94 (0.7)
K52 Colitis/gastroenteritis: Other noninfective inc. ischemic bowel	69 (0.9)	16 (0.4)	6 (0.5)	91 (0.7)
K85 Acute pancreatitis	65 (0.9)	7 (0.2)	3 (0.2)	75 (0.6)
S31 Trauma: penetrating	13 (0.2)	37 (0.9)	22 (1.7)	72 (0.6)
K51 Colitis/gastroenteritis: Ulcerative colitis	48 (0.7)	6 (0.2)	1 (0.1)	55 (0.4)
K561 Intestinal obstruction: Intussusception	16 (0.2)	14 (0.4)	25 (2.0)	55 (0.4)
Other	171 (2.3)	80 (2.0)	105 (8.2)	356 (2.8)
Missing	0 (0.0)	3 (0.1)	0 (0.0)	0 (0.0)

Numbers are n (%), unless otherwise indicated. ICD-10, International Statistical Classification of Diseases and Related Health Problems classification system.

Table 3s – Surgical site infection by Human Development Index and contamination

	High (n = 7,339)	Middle (n = 3,918)	Low (n = 1,282)
Clean-contaminated	449/5918 (7.6)	348/3126 (11.1)	141/878 (16.1)
Contaminated	138/779 (17.7)	125/542 (23.1)	84/219 (38.4)
Dirty	102/574 (17.8)	74/236 (31.4)	72/181 (39.8)

Numbers are number had a surgical site infection (%) / total number of patients in group (%).

Table 4s – Factors associated with SSI

		No SSI	Had SSI	Univariable Odds Ratio (95% confidence interval)	Multilevel Odds Ratio (95% credible interval)
HDI tertile	High	6648 (90.6)	691 (9.4)	-	-
	Middle	3369 (86.0)	549 (14.0)	1.57 (1.39-1.77, p<0.001)	1.12 (0.77-1.61, p=0.539)
	Low	984 (76.8)	298 (23.2)	2.91 (2.50-3.39, p<0.001)	1.60 (1.05-2.37, p=0.030)
Age (years)	Mean (SD)	43.3 (21.2)	45.1 (22.2)	1.01 (1.00-1.01, p<0.001)	1.00 (1.00-1.01, p=0.118)
Gender	Male	4678 (86.1)	756 (13.9)	-	-
	Female	5755 (89.1)	705 (10.9)	0.76 (0.68-0.85, p<0.001)	-
ASA	I	4930 (89.9)	554 (10.1)	-	-
	II	4141 (88.0)	565 (12.0)	1.21 (1.07-1.37, p=0.002)	1.44 (1.22-1.70, p<0.001)
	III+	1643 (81.6)	371 (18.4)	2.01 (1.74-2.32, p<0.001)	1.63 (1.32-2.02, p<0.001)
	Unknown	287 (85.9)	47 (14.1)	1.46 (1.05-1.99, p=0.021)	1.43 (0.95-2.12, p=0.082)
HIV	No	10957 (87.8)	1525 (12.2)	-	-
	Yes	44 (77.2)	13 (22.8)	2.12 (1.10-3.84, p=0.018)	-
Malaria	No	10985 (87.8)	1533 (12.2)	-	-
	Yes	16 (76.2)	5 (23.8)	2.24 (0.73-5.73, p=0.116)	-
Diabetes	No	10069 (88.2)	1343 (11.8)	-	-
	Yes	932 (82.7)	195 (17.3)	1.57 (1.33-1.85, p<0.001)	1.40 (1.15-1.70, p=0.001)
Immunosuppressive medication	No	10499 (88.0)	1426 (12.0)	-	-
	Yes	502 (81.8)	112 (18.2)	1.64 (1.32-2.02, p<0.001)	1.12 (0.87-1.44, p=0.381)
Current smoker	No	9421 (87.9)	1292 (12.1)	-	-
	Yes	1580 (86.5)	246 (13.5)	1.14 (0.98-1.31, p=0.089)	1.13 (0.96-1.34, p=0.145)
Pathology	Appendicitis	3613 (88.6)	466 (11.4)	-	-
	Gallstone disease	4001 (93.3)	287 (6.7)	0.56 (0.48-0.65, p<0.001)	0.86 (0.70-1.05, p=0.137)
	Malignancy	1573 (82.7)	328 (17.3)	1.62 (1.39-1.88, p<0.001)	1.35 (1.08-1.68, p=0.007)
	Benign foregut	647 (90.5)	68 (9.5)	0.81 (0.62-1.06, p=0.134)	0.82 (0.60-1.11, p=0.196)
	Benign midgut/hindgut	642 (76.3)	199 (23.7)	2.40 (1.99-2.89, p<0.001)	1.54 (1.22-1.94, p<0.001)
	Infection	100 (66.7)	50 (33.3)	3.88 (2.71-5.49, p<0.001)	2.46 (1.63-3.74, p<0.001)
	Congenital	136 (75.1)	45 (24.9)	2.57 (1.79-3.61, p<0.001)	1.90 (1.09-3.27, p=0.027)
	Trauma/injury	74 (67.3)	36 (32.7)	3.77 (2.48-5.64, p<0.001)	1.66 (1.05-2.59, p=0.031)
	Complication of previous procedure	67 (64.4)	37 (35.6)	4.28 (2.81-6.43, p<0.001)	2.02 (1.26-3.18, p=0.004)
	Other	40 (83.3)	8 (16.7)	1.55 (0.67-3.16, p=0.261)	1.13 (0.51-2.33, p=0.744)
	No Disease	108 (90.8)	11 (9.2)	0.79 (0.40-1.41, p=0.461)	0.80 (0.43-1.43, p=0.476)
Urgency	Elective	5743 (89.4)	678 (10.6)	-	-
	Emergency	5257 (85.9)	860 (14.1)	1.39 (1.24-1.54, p<0.001)	-
Operative approach	Open	4711 (80.0)	1176 (20.0)	-	-
	Laparoscopic	6290 (94.6)	362 (5.4)	0.23 (0.20-0.26, p<0.001)	0.36 (0.30-0.42, p<0.001)
Epidural	No	10082 (88.1)	1368 (11.9)	-	-
	Yes	755 (83.5)	149 (16.5)	1.45 (1.21-1.74, p<0.001)	-
	Unknown	164 (88.6)	21 (11.4)	0.94 (0.58-1.46, p=0.804)	-
Antibiotics: pre- or prophylactic	No	1233 (90.0)	137 (10.0)	-	-
	Yes	9675 (87.5)	1387 (12.5)	1.29 (1.08-1.56, p=0.007)	0.81 (0.66-1.01, p=0.065)
Intraoperative contamination	Clean-contaminated	8984 (90.5)	938 (9.5)	-	-
	Contaminated	1193 (77.5)	347 (22.5)	2.79 (2.43-3.19, p<0.001)	2.13 (1.81-2.51, p<0.001)
	Dirty	743 (75.0)	248 (25.0)	3.20 (2.72-3.74, p<0.001)	2.24 (1.83-2.73, p<0.001)
Checklist	No, not available	1947 (86.2)	312 (13.8)	-	-
	No, but available	1041 (80.6)	250 (19.4)	1.50 (1.25-1.80, p<0.001)	1.49 (1.15-1.91, p=0.002)
	Yes	7883 (89.1)	960 (10.9)	0.76 (0.66-0.87, p<0.001)	1.01 (0.79-1.28, p=0.970)
	Unknown	129 (89.0)	16 (11.0)	0.77 (0.44-1.28, p=0.346)	1.25 (0.69-2.21, p=0.440)

HDI, Human Development Index. ASA, American Society of Anesthesiologists classification grade. HIV, Human Immunodeficiency Virus.

Table 5s – Associations between country human development index and outcomes

Outcome		High (n = 7,339)	Middle (n = 3,918)	Low (n = 1,282)	Total (n = 12,539)	P-value
SSI	No, did not have an SSI	6648 (90.6)	3369 (86.0)	984 (76.8)	11001 (87.7)	<0.001
	Yes, before discharged from hospital	385 (5.2)	257 (6.6)	200 (15.6)	842 (6.7)	
	Yes, after discharged from hospital	306 (4.2)	292 (7.5)	98 (7.6)	696 (5.6)	
	No	6648 (90.6)	3369 (86.0)	984 (76.8)	11001 (87.7)	
	Yes	691 (9.4)	549 (14.0)	298 (23.2)	1538 (12.3)	
30-day mortality	Alive	7130 (97.2)	3768 (96.2)	1205 (94.0)	12103 (96.5)	<0.001
	Dead	110 (1.5)	64 (1.6)	61 (4.8)	235 (1.9)	
	Unknown	97 (1.3)	86 (2.2)	15 (1.2)	198 (1.6)	
	Missing	2 (0.0)	0 (0.0)	1 (0.1)	3 (0.0)	
Re-intervention	No	6975 (95.0)	3707 (94.6)	1194 (93.1)	11876 (94.7)	<0.001
	Yes	325 (4.4)	143 (3.6)	83 (6.5)	551 (4.4)	
	Unknown	35 (0.5)	68 (1.7)	4 (0.3)	107 (0.9)	
	Missing	4 (0.1)	0 (0.0)	1 (0.1)	5 (0.0)	
Abscess	No	7035 (95.9)	3743 (95.5)	1210 (94.4)	11988 (95.6)	<0.001
	Yes	254 (3.5)	101 (2.6)	67 (5.2)	422 (3.4)	
	Unknown	48 (0.7)	74 (1.9)	5 (0.4)	127 (1.0)	
	Missing	2 (0.0)	0 (0.0)	0 (0.0)	2 (0.0)	
Other HAI	No	6940 (94.6)	3722 (95.0)	1176 (91.7)	11838 (94.4)	<0.001
	Yes	370 (5.0)	133 (3.4)	99 (7.7)	602 (4.8)	
	Missing	29 (0.4)	63 (1.6)	7 (0.5)	99 (0.8)	
Length of stay	Median (IQR)	3 (6)	2 (3)	4 (6)	-	<0.001*

Numbers are n (%), unless otherwise indicated. All tests are chi-square, except when indicated by *, where a Kruskall-Wallis test has been applied. HAI – Hospital-associated infection. IQR- interquartile range

Table 6s – Antibiotics before surgery (pre-surgery) and/or at time of surgery (prophylaxis) by human development index tertile

		High (n = 7,339)	Middle (n = 3,918)	Low (n = 1,282)	Total (n = 12,539)	P-value
Antibiotics: pre-surgery	No	5774 (78.7)	2758 (70.4)	741 (57.8)	9273 (74.0)	<0.001
	Yes	1497 (20.4)	1079 (27.5)	517 (40.3)	3093 (24.7)	
	Unknown	68 (0.9)	81 (2.1)	24 (1.9)	173 (1.4)	
Antibiotics pre-surgery (days)	0-1 days	840 (11.4)	587 (15.0)	276 (21.5)	1703 (13.6)	<0.001
	2 days	216 (2.9)	136 (3.5)	124 (9.7)	476 (3.8)	
	3 days	117 (1.6)	93 (2.4)	43 (3.4)	253 (2.0)	
	4 days	65 (0.9)	37 (0.9)	15 (1.2)	117 (0.9)	
	5+ days	255 (3.5)	180 (4.6)	59 (4.6)	494 (3.9)	
	Unknown	5 (0.1)	46 (1.2)	0 (0.0)	51 (0.4)	
Antibiotics: prophylaxis	No	1221 (16.6)	835 (21.3)	124 (9.7)	2180 (17.4)	<0.001
	Yes	6058 (82.5)	3024 (77.2)	1143 (89.2)	10225 (81.5)	
	Unknown	59 (0.8)	59 (1.5)	15 (1.2)	133 (1.1)	
	Missing	1 (0.0)	0 (0.0)	0 (0.0)	1 (0.0)	
Antibiotics: pre- or prophylactic	No	848 (11.6)	472 (12.0)	50 (3.9)	1370 (10.9)	<0.001
	Yes	6446 (87.8)	3392 (86.6)	1224 (95.5)	11062 (88.2)	
	Missing	45 (0.6)	54 (1.4)	8 (0.6)	107 (0.9)	

Numbers are n (%), unless otherwise indicated. All tests are Pearson chi-squared.

Table 7s – Antibiotics following surgery (post-surgery) by human development index tertile

		High (n = 7,339)	Middle (n = 3,918)	Low (n = 1,282)	Total (n = 12,539)	P-value
Antibiotics: post-surgery	No	3872 (52.8)	707 (18.0)	177 (13.8)	4756 (37.9)	<0.001
	Yes	3376 (46.0)	3135 (80.0)	1098 (85.6)	7609 (60.7)	
	Unknown	89 (1.2)	76 (1.9)	6 (0.5)	171 (1.4)	
	Missing	2 (0.0)	0 (0.0)	1 (0.1)	3 (0.0)	
Antibiotics post-surgery (days)	0-1 days	637 (8.7)	433 (11.1)	123 (9.6)	1193 (9.5)	<0.001
	2 days	395 (5.4)	332 (8.5)	161 (12.6)	888 (7.1)	
	3 days	344 (4.7)	280 (7.1)	114 (8.9)	738 (5.9)	
	4 days	158 (2.2)	161 (4.1)	50 (3.9)	369 (2.9)	
	5+ days	1830 (24.9)	1837 (46.9)	650 (50.7)	4317 (34.4)	
	Unknown	12 (0.2)	92 (2.3)	0 (0.0)	104 (0.8)	
	None	3963 (54.0)	783 (20.0)	184 (14.4)	4930 (39.3)	

Numbers are n (%), unless otherwise indicated. All tests are Pearson chi-squared.

Table 8s - Factors associated with continuation of antibiotics following surgery

		No post-operative antibiotics	Post-operative antibiotics	P-value	Univariable Odds Ratio (95% confidence interval)	Multilevel Odds Ratio (95% credible)
HDI tertile	High	3872 (81.4)	3376 (44.4)	<0.001	-	-
	Middle	707 (14.9)	3135 (41.2)		5.09 (4.63-5.59, p<0.001)	2.02 (0.81-5.09, p=0.136)
	Low	177 (3.7)	1098 (14.4)		7.11 (6.05-8.42, p<0.001)	4.37 (1.65-11.85, p=0.002)
Age (in completed years)	Mean (SD)	46.7 (20.2)	41.3 (21.7)	<0.001	-	-
Gender	Male	1834 (38.6)	3516 (46.2)	<0.001	-	-
	Female	2682 (56.4)	3693 (48.5)		0.72 (0.67-0.77, p<0.001)	0.90 (0.81-1.01, p=0.081)
	Missing	240 (5.0)	400 (5.3)		-	-
ASA	I	1754 (36.9)	3678 (48.3)	<0.001	-	-
	II	2156 (45.3)	2485 (32.7)		0.55 (0.51-0.60, p<0.001)	1.09 (0.94-1.26, p=0.263)
	III+	781 (16.4)	1192 (15.7)		0.73 (0.65-0.81, p<0.001)	1.29 (1.04-1.60, p=0.018)
	Unknown	65 (1.4)	253 (3.3)		1.86 (1.41-2.47, p<0.001)	1.36 (0.89-2.07, p=0.154)
	Missing	0 (0.0)	1 (0.0)		-	-
HIV	No	4736 (99.6)	7573 (99.5)	0.672	-	-
	Yes	20 (0.4)	36 (0.5)		1.13 (0.66-1.98, p=0.672)	0.74 (0.35-1.57, p=0.438)
Malaria	No	4751 (99.9)	7593 (99.8)	0.167	-	-
	Yes	5 (0.1)	16 (0.2)		2.00 (0.78-6.13, p=0.176)	0.81 (0.28-2.38, p=0.683)
Diabetes	No	4332 (91.1)	6925 (91.0)	0.888	-	-
	Yes	424 (8.9)	684 (9.0)		1.01 (0.89-1.15, p=0.888)	1.12 (0.93-1.36, p=0.234)
Immunosuppressive medication	No	4513 (94.9)	7243 (95.2)	0.454	-	-
	Yes	243 (5.1)	366 (4.8)		0.94 (0.80-1.11, p=0.454)	1.18 (0.92-1.53, p=0.196)
Current smoker	No	4013 (84.4)	6544 (86.0)	0.013	-	-
	Yes	743 (15.6)	1065 (14.0)		0.88 (0.79-0.97, p=0.013)	0.86 (0.74-1.01, p=0.066)
Pathology	Appendicitis	1119 (23.5)	2916 (38.3)	<0.001	-	-
	Gallstone disease	2129 (44.8)	2109 (27.7)		0.38 (0.35-0.42, p<0.001)	0.92 (0.76-1.11, p=0.354)
	Malignancy	789 (16.6)	1072 (14.1)		0.52 (0.46-0.58, p<0.001)	1.24 (0.97-1.60, p=0.092)
	Benign foregut	356 (7.5)	351 (4.6)		0.38 (0.32-0.45, p<0.001)	0.93 (0.70-1.24, p=0.626)
	Benign midgut/hindgut	214 (4.5)	610 (8.0)		1.09 (0.92-1.30, p=0.302)	1.86 (1.39-2.50, p<0.001)
	Infection	31 (0.7)	117 (1.5)		1.45 (0.98-2.20, p=0.071)	0.64 (0.37-1.11, p=0.111)
	Congenital	19 (0.4)	158 (2.1)		3.19 (2.03-5.33, p<0.001)	2.05 (0.98-4.47, p=0.058)
	Trauma/injury	13 (0.3)	97 (1.3)		2.86 (1.66-5.38, p<0.001)	1.34 (0.64-2.93, p=0.443)
	Complication of previous procedure	22 (0.5)	81 (1.1)		1.41 (0.89-2.33, p=0.155)	1.82 (0.96-3.51, p=0.066)
	Other	21 (0.4)	25 (0.3)		0.46 (0.25-0.83, p=0.009)	0.96 (0.40-2.27, p=0.919)
	No Disease	43 (0.9)	70 (0.9)		0.62 (0.43-0.93, p=0.017)	0.54 (0.32-0.92, p=0.024)
	Missing	0 (0.0)	3 (0.0)		-	-
Urgency	Elective	3096 (65.1)	3225 (42.4)	<0.001	-	-
	Emergency	1660 (34.9)	4383 (57.6)		2.53 (2.35-2.73, p<0.001)	3.83 (3.27-4.51, p<0.001)
	Missing	0 (0.0)	1 (0.0)		-	-
Operative approach	Open	1326 (27.9)	4458 (58.6)	<0.001	-	-
	Laparoscopic	3430 (72.1)	3151 (41.4)		0.27 (0.25-0.30, p<0.001)	0.60 (0.51-0.69, p<0.001)
Epidural	No	4292 (90.2)	7022 (92.3)	<0.001	-	-
	Yes	375 (7.9)	511 (6.7)		0.83 (0.73-0.96, p=0.010)	1.03 (0.82-1.29, p=0.788)
	Unknown	89 (1.9)	76 (1.0)		0.52 (0.38-0.71, p<0.001)	0.95 (0.56-1.61, p=0.862)
Checklist	No, not available	513 (10.8)	1736 (22.8)	<0.001	-	-
	No, but available	227 (4.8)	1051 (13.8)		1.37 (1.15-1.63, p<0.001)	1.04 (0.74-1.46, p=0.840)
	Yes	3968 (83.4)	4729 (62.2)		0.35 (0.32-0.39, p<0.001)	0.97 (0.68-1.37, p=0.854)
	Unknown	48 (1.0)	92 (1.2)		0.57 (0.40-0.82, p=0.002)	0.70 (0.39-1.26, p=0.244)
	Missing	0 (0.0)	1 (0.0)		-	-
Intraoperative contamination	Clean-contaminated	4289 (90.2)	5493 (72.2)	<0.001	-	-
	Contaminated	334 (7.0)	1185 (15.6)		2.77 (2.44-3.15, p<0.001)	4.52 (3.69-5.54, p<0.001)
	Dirty	87 (1.8)	896 (11.8)		8.04 (6.47-10.13, p<0.001)	16.05 (11.81-22.09, p<0.001)
	Missing	46 (1.0)	35 (0.5)		-	-

HDI – Human Development Index. ASA - American Society of Anesthesiologists classification grade. HIV – Human Immunodeficiency Virus.

Figure 1s – Multilevel model of factors associated with post-surgery antibiotic use.

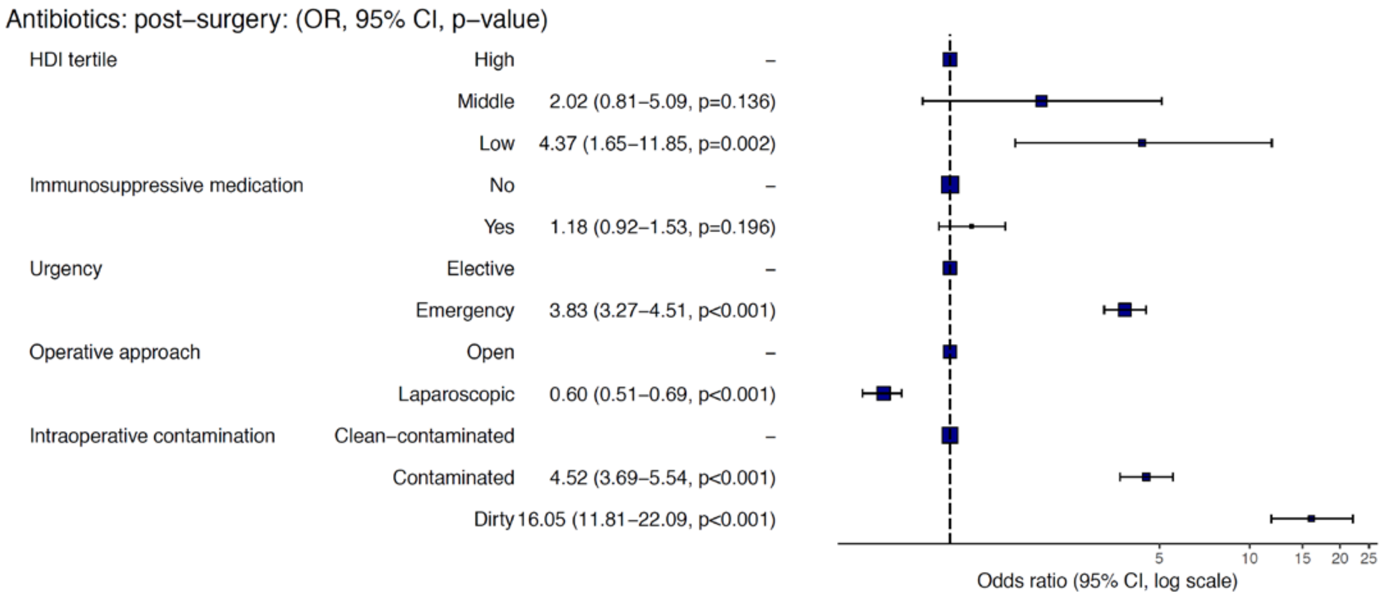

Full data contained in table 6s. HDI – human development index. OR – odds ratio. CI – credible interval.

Table 9s – Likely origin of organism(s) by human development index tertile

Origin	High (n = 299)	Middle (n = 89)	Low (n = 84)	Total (n = 472)	P-value
Bowel	205 (68.6)	50 (56.2)	46 (54.8)	301 (63.8)	0.015
Skin	55 (18.4)	22 (24.7)	20 (23.8)	97 (20.6)	
Mixed	28 (9.4)	9 (10.1)	16 (19.0)	53 (11.2)	
Other organism(s)	11 (3.7)	8 (9.0)	2 (2.4)	21 (4.4)	

Numbers are n (%), unless otherwise indicated. Test is Pearson chi-squared. The likely origin of organisms was broadly categorised simply to aid interpretation. The likely origin was considered ‘bowel’ if cultures grew only gram-negative bacilli, *Enterococcus* species, or anaerobic organisms; ‘skin’ if cultures only grew skin organisms such as *Staphylococcus* species; and ‘mixed’ if they grew both. ‘Other organism(s)’ could not be further classified.

Table 10s - All organisms cultured from wound swabs by human development index tertile

Isolated Organism Type	High (n = 299)	Middle (n = 89)	Low (n = 84)	Total (n = 472)
Gram-negative bacilli	96 (32.1)	21 (23.6)	32 (38.1)	149 (31.6)
Staphylococcus aureus	28 (9.4)	18 (20.2)	16 (19.0)	62 (13.1)
Anaerobe	35 (11.7)	16 (18.0)	9 (10.7)	60 (12.7)
Anaerobe + Gram-negative bacilli	32 (10.7)	4 (4.5)	4 (4.8)	40 (8.5)
Gram-negative bacilli + *Staphylococcus aureus*	12 (4.0)	5 (5.6)	6 (7.1)	23 (4.9)
Other organism	11 (3.7)	8 (9.0)	2 (2.4)	21 (4.4)
Pseudomonas species	10 (3.3)	1 (1.1)	3 (3.6)	14 (3.0)
Anaerobe + Gram-negative bacilli + *Staphylococcus aureus*	2 (0.7)	0 (0.0)	8 (9.5)	10 (2.1)
Coagulase-negative staphylococcus	6 (2.0)	3 (3.4)	1 (1.2)	10 (2.1)
Enterococcus species	9 (3.0)	0 (0.0)	0 (0.0)	9 (1.9)
Gram-negative bacilli + *Enterococcus* species	4 (1.3)	4 (4.5)	0 (0.0)	8 (1.7)
Streptococcus species	8 (2.7)	0 (0.0)	0 (0.0)	8 (1.7)
Anaerobe + *Staphylococcus aureus*	2 (0.7)	3 (3.4)	2 (2.4)	7 (1.5)
Candida species	6 (2.0)	1 (1.1)	0 (0.0)	7 (1.5)
Anaerobe + Gram-negative bacilli + *Streptococcus* species	3 (1.0)	0 (0.0)	0 (0.0)	3 (0.6)
Anaerobe + *Pseudomonas* species	3 (1.0)	0 (0.0)	0 (0.0)	3 (0.6)
Candida species + Gram-negative bacilli	3 (1.0)	0 (0.0)	0 (0.0)	3 (0.6)
Gram-negative bacilli + *Streptococcus* species	3 (1.0)	0 (0.0)	0 (0.0)	3 (0.6)
Anaerobe + *Candida* species + Gram-negative bacilli	1 (0.3)	0 (0.0)	1 (1.2)	2 (0.4)
Anaerobe + Gram-negative bacilli + *Enterococcus* species	1 (0.3)	1 (1.1)	0 (0.0)	2 (0.4)
Anaerobe + Gram-negative bacilli + *Pseudomonas* species	1 (0.3)	1 (1.1)	0 (0.0)	2 (0.4)
Anaerobe + *Staphylococcus aureus* + *Streptococcus* species	1 (0.3)	1 (1.1)	0 (0.0)	2 (0.4)
Anaerobe + *Streptococcus* species	2 (0.7)	0 (0.0)	0 (0.0)	2 (0.4)
Gram-negative bacilli + *Pseudomonas* species	1 (0.3)	1 (1.1)	0 (0.0)	2 (0.4)
Enterococcus species + *Pseudomonas* species	2 (0.7)	0 (0.0)	0 (0.0)	2 (0.4)
Staphylococcus aureus + *Streptococcus* species	2 (0.7)	0 (0.0)	0 (0.0)	2 (0.4)
Anaerobe + *Candida* species	1 (0.3)	0 (0.0)	0 (0.0)	1 (0.2)
Anaerobe + *Candida* species + *Staphylococcus aureus*	1 (0.3)	0 (0.0)	0 (0.0)	1 (0.2)
Anaerobe + Gram-negative bacilli + *Enterococcus* species + Coagulase-negative staphylococcus	1 (0.3)	0 (0.0)	0 (0.0)	1 (0.2)
Anaerobe + *Enterococcus* species	1 (0.3)	0 (0.0)	0 (0.0)	1 (0.2)
Anaerobe + Coagulase-negative staphylococcus	1 (0.3)	0 (0.0)	0 (0.0)	1 (0.2)
Anaerobe + *Pseudomonas* species + *Staphylococcus aureus*	1 (0.3)	0 (0.0)	0 (0.0)	1 (0.2)
Candida species + Gram-negative bacilli + *Enterococcus* species	1 (0.3)	0 (0.0)	0 (0.0)	1 (0.2)
Candida species + Gram-negative bacilli + *Enterococcus* species + *Pseudomonas* species	1 (0.3)	0 (0.0)	0 (0.0)	1 (0.2)
Candida species + *Enterococcus* species	1 (0.3)	0 (0.0)	0 (0.0)	1 (0.2)
Candida species + Coagulase-negative staphylococcus	1 (0.3)	0 (0.0)	0 (0.0)	1 (0.2)
Gram-negative bacilli + *Enterococcus* species + *Pseudomonas* species	0 (0.0)	1 (1.1)	0 (0.0)	1 (0.2)
Gram-negative bacilli + Coagulase-negative staphylococcus	1 (0.3)	0 (0.0)	0 (0.0)	1 (0.2)
Gram-negative bacilli + *Staphylococcus aureus* + Coagulase-negative staphylococcus	1 (0.3)	0 (0.0)	0 (0.0)	1 (0.2)
Streptococcus species + Coagulase-negative staphylococcus	1 (0.3)	0 (0.0)	0 (0.0)	1 (0.2)

Numbers are n (%).

Table 11s – Missing data in final dataset

	High (n = 7,339)	Middle (n = 3,918)	Low (n = 1,282)	Total (n = 12,539)
Age	0 (0.0)	2 (0.1)	0 (0.0)	2 (0.0)
ASA	0 (0.0)	0 (0.0)	1 (0.1)	1 (0.0)
HIV	0 (0.0)	0 (0.0)	1 (0.1)	1 (0.0)
Malaria	1 (0.0)	1 (0.0)	0 (0.0)	2 (0.0)
Diabetes	0 (0.0)	1 (0.0)	0 (0.0)	1 (0.0)
Immunosuppressive medication	0 (0.0)	0 (0.0)	0 (0.0)	0 (0.0)
Current smoker	0 (0.0)	0 (0.0)	0 (0.0)	0 (0.0)
Pathology	0 (0.0)	3 (0.1)	0 (0.0)	3 (0.0)
Procedure start-time	6 (0.1)	2 (0.1)	0 (0.0)	8 (0.1)
Urgency	1 (0.0)	0 (0.0)	0 (0.0)	1 (0.0)
Checklist	1 (0.0)	0 (0.0)	0 (0.0)	1 (0.0)
Operative approach	0 (0.0)	0 (0.0)	0 (0.0)	0 (0.0)
Antibiotics: pre- or prophylactic	45 (0.6)	54 (1.4)	8 (0.6)	107 (0.9)
Intraoperative contamination	68 (0.9)	14 (0.4)	4 (0.3)	86 (0.7)
SSI	0 (0.0)	0 (0.0)	0 (0.0)	0 (0.0)
30-day mortality	99 (1.3)	86 (2.2)	16 (1.2)	201 (1.6)
30-day reintervention	39 (0.5)	68 (1.7)	5 (0.4)	112 (0.9)
Other HAI	29 (0.4)	63 (1.6)	7 (0.5)	99 (0.8)
Length of stay	3 (0.1)	3 (0.1)	1 (0.1)	(0.1)

Patterns of missing data in final dataset for variables included in multivariable models. Data are n (%).

Table 12s – Missing data: characteristics of patients excluded due to unknown/missing primary outcome measure (SSI).

		High (n = 280)	Middle (n = 379)	Low (n = 53)	Total (n = 712)	P-value
Age (years)	Mean (SD)	43.6 (20.9)	34 (17)	37.8 (22.2)	38.1 (19.5)	<0.001
Gender	Male	131 (46.8)	149 (39.3)	26 (49.1)	306 (43.0)	0.109
	Female	138 (49.3)	220 (58.0)	27 (50.9)	385 (54.1)	
	Missing	11 (3.9)	10 (2.6)	0 (0.0)	21 (2.9)	
ASA	1	125 (44.6)	240 (63.3)	20 (37.7)	385 (54.1)	<0.001
	2	95 (33.9)	75 (19.8)	26 (49.1)	196 (27.5)	
	3+	53 (18.9)	16 (4.2)	6 (11.3)	75 (10.5)	
	Unknown	7 (2.5)	48 (12.7)	1 (1.9)	56 (7.9)	
HIV	No	255 (91.1)	328 (86.5)	48 (90.6)	631 (88.6)	0.329
	Yes	2 (0.7)	7 (1.8)	0 (0.0)	9 (1.3)	
	Unknown	23 (8.2)	44 (11.6)	5 (9.4)	72 (10.1)	
Malaria	No	267 (95.4)	368 (97.1)	51 (96.2)	686 (96.3)	0.595
	Yes	0 (0.0)	1 (0.3)	0 (0.0)	1 (0.1)	
	Unknown	13 (4.6)	10 (2.6)	2 (3.8)	25 (3.5)	
Diabetes	No	268 (95.7)	345 (91.0)	50 (94.3)	663 (93.1)	0.044
	Yes	10 (3.6)	19 (5.0)	3 (5.7)	32 (4.5)	
	Unknown	2 (0.7)	15 (4.0)	0 (0.0)	17 (2.4)	
Immunosuppressive medication	No	268 (95.7)	372 (98.2)	48 (90.6)	688 (96.6)	0.009
	Yes	12 (4.3)	7 (1.8)	5 (9.4)	24 (3.4)	
Current smoker	No	242 (86.4)	342 (90.2)	47 (88.7)	631 (88.6)	0.314
	Yes	38 (13.6)	37 (9.8)	6 (11.3)	81 (11.4)	
Pathology	Appendicitis	119 (42.5)	153 (40.4)	16 (30.2)	288 (40.4)	<0.001
	Gallstone disease	112 (40.0)	130 (34.3)	16 (30.2)	258 (36.2)	
	Malignancy	20 (7.1)	14 (3.7)	4 (7.5)	38 (5.3)	
	Benign foregut	7 (2.5)	27 (7.1)	3 (5.7)	37 (5.2)	
	Benign midgut/hindgut	14 (5.0)	16 (4.2)	5 (9.4)	35 (4.9)	
	Infection	0 (0.0)	16 (4.2)	7 (13.2)	23 (3.2)	
	Congenital	2 (0.7)	6 (1.6)	1 (1.9)	9 (1.3)	
	Trauma/injury	2 (0.7)	7 (1.8)	1 (1.9)	10 (1.4)	
	Complication of previous procedure	0 (0.0)	3 (0.8)	0 (0.0)	3 (0.4)	
	Other	0 (0.0)	2 (0.5)	0 (0.0)	2 (0.3)	
	No Disease	4 (1.4)	5 (1.3)	0 (0.0)	9 (1.3)	
Procedure start-time	08:00-18:00	198 (70.7)	246 (64.9)	34 (64.2)	478 (67.1)	0.027
	18:00-22:00	41 (14.6)	39 (10.3)	4 (7.5)	84 (11.8)	
	22:00 to 08:00	41 (14.6)	93 (24.5)	15 (28.3)	149 (20.9)	
	Missing	0 (0.0)	1 (0.3)	0 (0.0)	1 (0.1)	
Admission to procedure time (h)	<6	99 (35.4)	150 (39.6)	10 (18.9)	259 (36.4)	<0.001
	6-11	35 (12.5)	31 (8.2)	8 (15.1)	74 (10.4)	
	12-23	49 (17.5)	51 (13.5)	6 (11.3)	106 (14.9)	
	24-47	48 (17.1)	43 (11.3)	4 (7.5)	95 (13.3)	
	48+	36 (12.9)	84 (22.2)	14 (26.4)	134 (18.8)	
	Missing	13 (4.6)	20 (5.3)	11 (20.8)	44 (6.2)	
Urgency	Elective	96 (34.3)	156 (41.2)	19 (35.8)	271 (38.1)	0.188
	Emergency	184 (65.7)	223 (58.8)	34 (64.2)	441 (61.9)	
Checklist	No, not available	72 (25.7)	105 (27.7)	5 (9.4)	182 (25.6)	<0.001
	No, but available	8 (2.9)	65 (17.2)	24 (45.3)	97 (13.6)	
	Yes	196 (70.0)	200 (52.8)	24 (45.3)	420 (59.0)	
	Unknown	4 (1.4)	9 (2.4)	0 (0.0)	13 (1.8)	
Operative approach	Open	68 (24.3)	247 (65.2)	41 (77.4)	356 (50.0)	<0.001
	Laparoscopic	212 (75.7)	132 (34.8)	12 (22.6)	356 (50.0)	
Epidural	No	214 (76.4)	346 (91.3)	53 (100.0)	613 (86.1)	<0.001
	Yes	9 (3.2)	26 (6.9)	0 (0.0)	35 (4.9)	
	Unknown	57 (20.4)	7 (1.8)	0 (0.0)	64 (9.0)	
Antibiotics: pre- or prophylactic	No	29 (10.4)	31 (8.2)	4 (7.5)	64 (9.0)	0.185
	Yes	245 (87.5)	326 (86.0)	47 (88.7)	618 (86.8)	
	Missing	6 (2.1)	22 (5.8)	2 (3.8)	30 (4.2)	
Intraoperative contamination	Clean-contaminated	232 (82.9)	311 (82.1)	42 (79.2)	585 (82.2)	0.262
	Contaminated	33 (11.8)	39 (10.3)	8 (15.1)	80 (11.2)	
	Dirty	9 (3.2)	26 (6.9)	2 (3.8)	37 (5.2)	
	Missing	6 (2.1)	3 (0.8)	1 (1.9)	10 (1.4)	

Number are n (%) unless otherwise indicated.

Table 13s – Missing data: secondary outcomes for patients excluded due to unknown/missing primary outcome measure (SSI).

		High (n = 280)	Middle (n = 379)	Low (n = 53)	Total (n = 712)	P-value
30-day mortality	Alive	132 (47.1)	106 (28.0)	7 (13.2)	245 (34.4)	<0.001
	Dead	9 (3.2)	14 (3.7)	1 (1.9)	24 (3.4)	
	Missing	139 (49.6)	259 (68.3)	45 (84.9)	443 (62.2)	
30-day reintervention	No	149 (53.2)	35 (9.2)	13 (24.5)	197 (27.7)	<0.001
	Yes	5 (1.8)	3 (0.8)	1 (1.9)	9 (1.3)	
	Missing	126 (45.0)	341 (90.0)	39 (73.6)	506 (71.1)	
Organ space infection (abscess)	No	95 (33.9)	44 (11.6)	8 (15.1)	147 (20.6)	<0.001
	Yes	0 (0.0)	0 (0.0)	1 (1.9)	1 (0.1)	
	Missing	185 (66.1)	335 (88.4)	44 (83.0)	564 (79.2)	
Other HAI	No	145 (51.8)	72 (19.0)	18 (34.0)	235 (33.0)	<0.001
	Yes	6 (2.1)	6 (1.6)	2 (3.8)	14 (2.0)	
	Missing	129 (46.1)	301 (79.4)	33 (62.3)	463 (65.0)	
Length of stay	Mean (SD)	2.0 (3.0)	2.0 (2.0)	3.0 (3.0)	2.0 (3.0)	<0.001

Number are n (%) unless otherwise indicated.

Validation study outline

Data validation was performed in three parts across a sample of representative centres, as described in the GlobalSurg 2 study protocol (1) and the pre-specified validation protocol (2).

1. Centre questionnaires: self-reporting of key processes used to identify and follow-up patients.
2. Independent data validation: quantitative case ascertainment and sampled data accuracy.
3. Team interviews: qualitative collaborator process and system assessment.

Data relating to parts 1 and 2 are included here. The full report of the validation study will be published separately.

Centre questionnaire

All hospitals in all countries were included in a study of follow-up methods used by collaborators performing primary data collection.

Patient identification methods

Collaborators recorded for each included patient the primary and secondary methods by which the patient had been identified for inclusion (Table 2v). The majority of patients were identified using theatre logbook/computer systems (high 78.6%, middle 60%, low HDI (59.2%) and operating lists (high 18%, middle 21%, low 21.7%). There were more patients in low (18.5%) and middle HDI countries (14.0%) identified primarily using ward lists, compared with high HDI countries (2.5%). Very few patients were identified by staff memory alone.

Table 14s. Primary method of patient identification by human development index.

	Human development index		
	**High**	**Middle**	**Low**
Theatre logbook/system review	5771 (78.6)	2349 (60.0)	759 (59.2)
Planned operating lists	1318 (18.0)	823 (21.0)	278 (21.7)
Ward lists	181 (2.5)	549 (14.0)	237 (18.5)
Handover lists	50 (0.7)	154 (3.9)	6 (0.5)
Staff memory	19 (0.3)	42 (1.1)	2 (0.2)
Missing	0 (0.0)	1 (0.0)	0 (0.0)

Data are n (%).

Follow-up methods

Methods or follow-up are described in Table 1v. In low and middle HDI countries, ascertainment of true 30-day outcomes were high. Patients in whom collaborators had no contact after discharge were more frequent in high (16.3%), compared with middle (5.8%) and low (3.2%) HDI countries. Missing data rates were low.

Table 15s. Follow-up methods by human development index.

	Human development index		
	**High**	**Middle**	**Low**
Clinic review	3114 (42.4)	1016 (25.9)	642 (50.1)
Community/home review	106 (1.4)	12 (0.3)	1 (0.1)
No contact after discharge	1194 (16.3)	226 (5.8)	41 (3.2)
Still inpatient	208 (2.8)	81 (2.1)	112 (8.7)
Telephone review	2708 (36.9)	2582 (65.9)	483 (37.7)
Missing	9 (0.1)	1 (0.0)	3 (0.2)

Data are n (%).

Independent validation

Case ascertainment

Case ascertainment was performed by an independent assessor to determine the proportion of patient within inclusion criteria who were enrolled. Validators were asked to provide this information for a given time period prior to receiving the necessary information for the “Data accuracy” stage. Case ascertainment was performed in a sample of 66 hospitals (high 48, middle 10, low 8 HDI) in 25 countries (high 14, middle 6, low 5 HDI), representing 132 weeks of data collection.

There were 1378 included cases across hospitals and teams. Validators identified 1476 cases that fulfilled inclusion criteria for the validation period, equating to a case ascertainment rate of 93.3% (1378/1476).

There was no difference in rates of case ascertainment between hospitals in different HDI tertiles (chi-squared statistic 1.04, df 2, p=0.595)

There was variability in case ascertainment across all hospitals (min 48%, lower quartile 97%, median 100%, upper quartile 100%, max 130%) (Figure 2s). Note where case ascertainment is greater than 100%, the collaborators enrolled more cases than the validators could identify.

Figure 2s. Case ascertainment. Correlation of number of patients fulfilling inclusion criteria identified by validators vs. those included in primary data collection. Dotted lines represent 95% and 150% concordance.

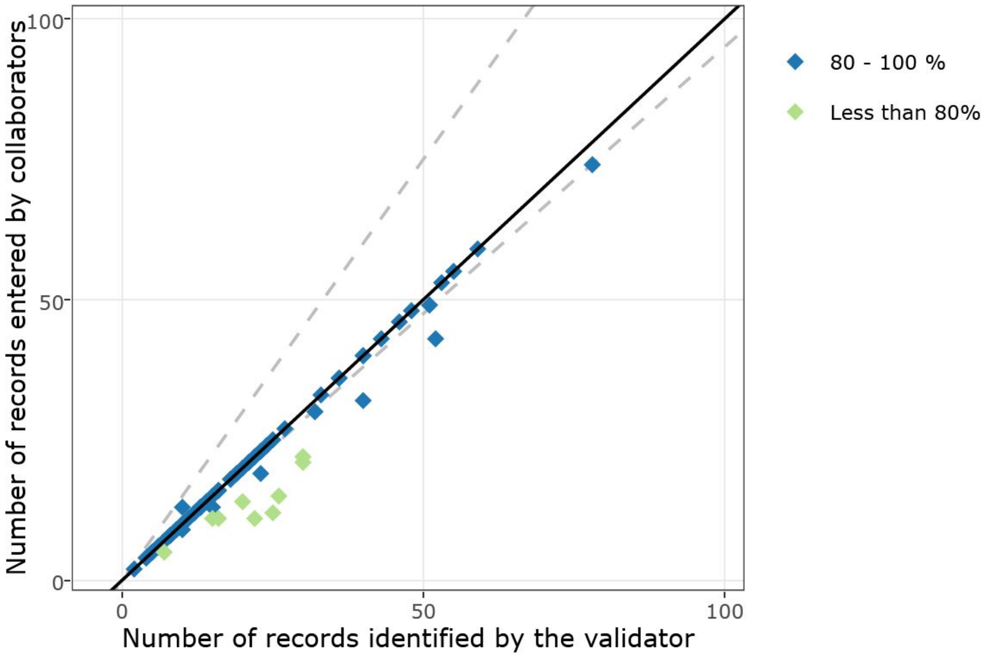

Data accuracy

A subset of collected variables were validated by individuals who were independent of the primary data collection process. Following the “case ascertainment” stage, validators were provided with the following patient variables: hospital ID, date of admission, date of operation, pathology/indication, and primary operation name. Using this information, validators were asked to determine the following variables from the patient’s paper or electronic notes: two patient variables (age and gender), two operation variables (urgency and operative approach), and two outcome measures (30-day reintervention and 30-day mortality). The primary outcome measure (surgical site infection) was intentionally not validated given data from the GlobalSurg 1 study showing the low rate with which SSI is reliably recorded in medical records.

Validation was attempted on 1776 included patients. Availability of sampled variables is detailed in table 15s. There was variation in the ability of validators to identify the primary operation name across HDI (high 98.8%, middle 98.4%, low (71.7%) HDI). There was particular difficulty in low HDI countries with some validators commenting about on the lack of availability of reliable written notes. This pattern was reflected across other patient and operation variables. The ability for validators to identify outcome measures was lower across all countries (reintervention occurrence available, high 90.5%, middle 79.9%, low 57.8% HDI; Table 15s).

Table 16s. Data accuracy study. Availability of sampled variables to validators by country HDI. * Chi-squared or Fisher’s exact test where expected cell count <5. Data are n (%).

		Human development index			
		High	Middle	Low	p-value*
Primary operation name available	No	16 (1.2)	3 (1.6)	58 (28.3)	<0.001
	Yes	1297 (98.8)	181 (98.4)	147 (71.7)	
Age available	No	10 (0.7)	1 (0.5)	52 (25.2)	<0.001
	Yes	1364 (99.3)	183 (99.5)	154 (74.8)	
Gender available	No	11 (0.8)	0 (0.0)	52 (25.2)	<0.001
	Yes	1362 (99.2)	184 (100.0)	154 (74.8)	
Urgency available	Yes	1355 (98.7)	158 (85.9)	152 (73.8)	<0.001
	No	18 (1.3)	26 (14.1)	54 (26.2)	
Operative approach available	No	30 (2.2)	2 (1.1)	52 (25.2)	<0.001
	Yes	1343 (97.8)	182 (98.9)	154 (74.8)	
30-day reintervention available	No	131 (9.5)	37 (20.1)	87 (42.2)	<0.001
	Yes	1241 (90.5)	147 (79.9)	119 (57.8)	
30-day mortality available	No	134 (9.8)	37 (20.1)	77 (37.4)	<0.001
	Yes	1238 (90.2)	147 (79.9)	129 (62.6)	

Age was the only continuous variable which was validated. Validator vs. primary data were highlight correlated (Pearson correlation coefficient 0.989, 95%C 0.987 to 0.990) (Table 16s; Figure 3s).

For categorical variables, agreement between validation and primary data is described using the Cohen's kappa coefficient. Somewhat arbitrary guidelines on the interpretation of agreement with the kappa coefficient are >0.75 excellent, 0.40 to 0.75 fair to good, and below 0.40 poor (3).

There was good agreement between validation and primary data for patient (gender, kappa coefficient 0.934) and operative (urgency 0.896; approach 0.928) categorical variables (Table 17s). Agreement for 30-day mortality (0.912) was also excellent, however, for 30-day reintervention (0.651) the agreement was significantly lower.

Table 17s. Validation study continuous variable.

	Primary data	Validation data	Pearson correlation coefficient (95% CI)
Age	45.3 (21.8)	44.8 (22.4)	0.989 (0.987 to 0.990)

Data are mean (standard deviation).

Figure 3s. Correlation of patient age in validation vs. primary datasets (n=1701).

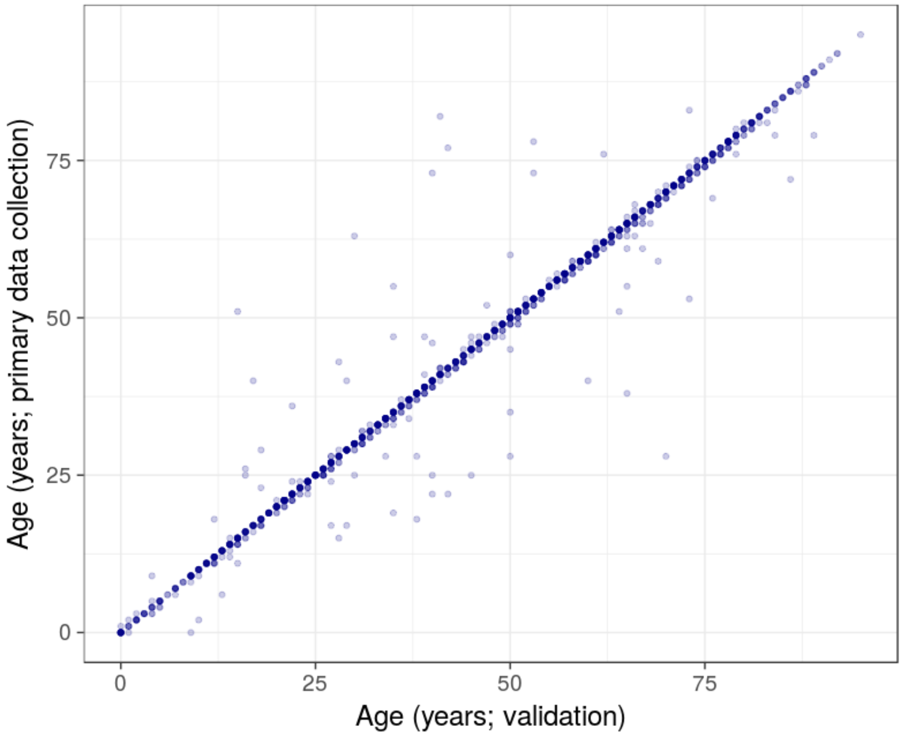

Table 18s. Validation study for categorical variables.

	Primary data	Validation data		Kappa
Gender		Male	Female	0.934
	Male	781	27	
	Female	28	838	
Urgency		Elective	Emergency	0.896
	Elective	781	46	
	Emergency	41	798	
Approach		Open	Laparoscopic	0.928
	Open	771	32	
	Laparoscopic	28	847	
30-day reintervention		No	Yes	0.651
	No	1369	45	
	Yes	19	66	
30-day mortality		Alive	Died	0.912
	Alive	1468	3	
	Died	3	32	

Data are counts

References

1. Determining the worldwide epidemiology of surgical site infections after gastrointestinal resection surgery: protocol for a multicentre, international, prospective cohort study (GlobalSurg 2) | BMJ Open [Internet]. [cited 2017 Jul 25]. Available from: http://bmjopen.bmj.com/content/7/7/e012150

2. GlobalSurg 2 Validation Protocol: A multicentre evaluation of prospective observational data and processes to validate a global surgical outcomes study [Internet]. [cited 2017 Jul 25]. Available from: http://gsmain.globalsurg.netdna-cdn.com/wp-content/uploads/2015/10/GlobalSurg-2-Validation-Protocol-v2.1.pdf.pdf

3. Fleiss JL. Statistical methods for rates and proportions. New York: Wiley; 1973. 223 p. (Wiley series in probability and mathematical statistics).

Collaborators

Writing group: Aneel Bhangu, Adesoji O Ademuyiwa, Maria Lorena Aguilera, Philip Alexander, Sara W Al-Saqqa, Giuliano Borda-Luque, Ainhoa Costas-Chavarri, Thomas M Drake, Faustin Ntirenganya, J Edward Fitzgerald, Stuart J Fergusson, James Glasbey, J.C Allen Ingabire, Lawani Ismaïl, Hosni Khairy Salem, Anyomih Theophilus Teddy Kojo, Marie Carmela Lapitan, Richard Lilford, Andre L Mihaljevic, Dion Morton, Alphonse Zeta Mutabazi, Dmitri Nepogodiev, Adewale O Adisa, Riinu Ots, Francesco Pata, Thomas Pinkney, Tomas Poškus, Ahmad Uzair Qureshi, Antonio Ramos-De la Medina, Sarah Rayne, Catherine A Shaw, Sebastian Shu, Richard Spence, Neil Smart, Stephen Tabiri, Ewen M Harrison

Study guarantors: Aneel Bhangu (aneelbhangu@gmail.com), Ewen M Harrison (ewen.harrison@gmail.com)

Patient representatives: Azmina Verjee, Emmy Runigamugabo

Protocol development: Chetan Khatri, Midhun Mohan, Thomas M Drake, James Glasbey, Dmitri Nepogodiev, Catherine A Shaw, Zahra Jaffry, Stuart J Fergusson, Francesco Pata, Adesoji O Ademuyiwa, Afnan Altamini, Hosni Khairy Salem, Andrew Kirby, Kjetil Søreide, Gustavo Recinos, Richard Spence, Sarah Rayne, Stephen Tabiri, Jen Cornick, Thomas Pinkney, Richard Lilford, J Edward Fitzgerald, Ewen M Harrison, Aneel Bhangu

National leads:

Argentina, Maria Marta Modolo; **Australia**, Dushyant Iyer, Sebastian King, Tom Arthur; **Bangladesh,** Sayeda Nazmum Nahar; **Barbados,** Ade Waterman; **Benin,** Lawani Ismaïl; **Botswana,** Michael Walsh; **Canada,** Arnav Agarwal, Augusto Zani, Mohammed Firdouse, Tyler Rouse; **China,** Qinyang Liu; **Colombia,** Juan Camilo Correa; **Egypt,** Hosni Khairy Salem; **Estonia,** Peep Talving; **Ethiopia,** Mengistu Worku; **France**, Alexis Arnaud; **Ghana,** Stephen Tabiri; **Greece,** Vassilis Kalles; **Guatemala**, Maria Lorena Aguilera, Gustavo Recinos; **India**, Basant Kumar, Sunil Kumar; **Indonesia**, Radhian Amandito; **Ireland**, Roy Quek; **Italy**, Francesco Pata, Luca Ansaloni; **Jordan**, Ahmed Altibi; **Lithuania**, Donatas Venskutonis, Justas Zilinskas, Tomas Poskus; **Madagascar**, John Whitaker; **Malawi**, Vanessa Msosa; **Malaysia**, Yong Yong Tew; **Malta**, Alexia Farrugia, Elaine Borg; **Mexico**, Antonio Ramos-De la Medina; **Morocco**, Zineb Bentounsi; **Nigeria**, Adesoji O Ademuyiwa; **Norway**, Kjetil Søreide; **Pakistan,** Tanzeela Gala; **Palestinian Territory,** Ibrahim Al-Slaibi, Haya Tahboub, Osaid H. Alser; **Peru,** Diego Romani, Sebestian Shu; **Poland,** Piotr Major; **Romania**, Aurel Mironescu, Matei Bratu, Amar Kourdouli; **Saint Kitts and Nevis,** Aliyu Ndajiwo; **Saudi Arabia,** Abdulaziz Altwijri, Mohammed Ubaid Alsaggaf, Ahmad Gudal, Al Faifi Jubran; **Sierra Leone,** Sam Seisay; **Singapore,** Bettina Lieske; **South Africa,** Sarah Rayne, Richard Spence; **Spain,** Irene Ortega; **Sri Lanka,** Jenifa Jeyakumar, Kithsiri J. Senanayake; **Sudan,** Omar Abdulbagi; **Sweden,** Yucel Cengiz; **Switzerland,** Dmitri Raptis; **Turkey,** Yuksel Altinel; **United Kingdom,** Chia Kong, Ella Teasdale, Gareth Irwin, Michael Stoddart, Rakan Kabariti, Sukrit Suresh; **United States,** Katherine Gash, Ragavan Narayanan; **Zambia,** Mayaba Maimbo

Local collaborators:

Albania: Besmir Grizhja, Shpetim Ymeri, Gezim Galiqi (Spitali Rajonal Shkoder);

Argentina: Roberto Klappenbach, Diego Antezana, Alvaro Enrique Mendoza Beleño, Cecilia Costa, Belen Sanchez, Susan Aviles (Hospital Zonal General De Agudos Simplemente Evita); Maria Marta Modolo, Claudio Gabriel Fermani, Rubén Balmaceda, Santiago Villalobos, Juan Manuel Carmona (Hospital Luis C. Lagomaggiore, Mendoza);

Australia: Daniel Hamill, Peter Deutschmann, Simone Sandler, Daniel Cox (Alice Springs Hospital); Ram Nataraja, Claire

Sharpin, Damir Ljuhar (Monash Medical Center); Demi Gray, Morgan Haines (Port Macquarie Base Hospital); Dush Iyer, Nithya Niranjan, Scott D'Amours (Liverpool Hospital); Morvarid Ashtari, Helena Franco, (Gold Coast University Hospital).

Bangladesh: Ashrarur Rahman Mitul, Sabbir Karim, (Dhaka Shishu (Children) Hospital); Nowrin F. Aman, Mahnuma Mahfuz Estee (Holy Family Red Crescent Medical College & Hospital); Umme Salma, Joyeta Razzaque, Tasnia Hamid Kanta (Dhaka Medical College And Hospital); Sayeeda Aktar Tori, Shadid Alamin, Swapnil Roy, Shadid Al Amin, Rezaul Karim (Armed Forces Medical College); Muhtarima Haque, Amreen Faruq, Farhana Iftekhar (Birdem Bangladesh Institute Of Research And Rehabilitation In Diabetes Endocrine And Metabolic Disorder General Hospital);

Barbados: Margaret O'Shea, Greg Padmore, Ramesh Jonnalagadda (Queen Elizabeth Hospital).

Belarus: Andrey Litvin, Aliaksandr Filatau, Dzmitry Paulouski, Maryna Shubianok, Tatsiana Shachykava (Gomel Regional Clinical Hospital); Dzianis Khokha, Vladimir Khokha (City Hospital)

Benin: Fernande Djivoh, Lawani Ismaïl, Francis Dossou (Centre National Hospitalier Et Universitaire Hubert Koutoukou Maga); Djifid Morel Seto, Dansou Gaspard Gbessi, Bruno Noukpozounkou, Yacoubou Imorou Souaibou, (Centre National Hospitalier Et Universitaire Hubert Koutoukou Maga); Kpèmahouton René Keke, Fred Hodonou (Clinique Vignon); Ernest Yemalin Stephane Ahounou, Thierry Alihonou (Hopital El Fateh); Max Dénakpo, Germain Ahlonsou (Hospital Saint Luc).

Botswana: Alemayehu Ginbo Bedada (Princess Marina Hospital).

Burundi: Carlos Nsengiyumva, Sandrine Kwizera, Venerand Barendegere (Hopital Militaire De Kamenge).

Cambodia: Philip Choi, Simon Stock (World Mate Emergency Hospital)

Canada: Luai Jamal, Mohammed Firdouse, Augusto Zani, Georges Azzie, Sameer Kushwaha, Arnav Agarwal (The Hospital For Sick Children).

China: Tzu-Ling Chen, Chingwan Yip (Fudan University Affiliated Huashan Hospital).

Colombia: Irene Montes, Felipe Zapata, Sebastian Sierra (Clinica CES); Maria Isabel Villegas Lanau, Maria Clara Mendoza Arango, Ivan Mendoza Restrepo (Clinica Las Vegas), Sebastian Sierra, Ruben Santiago Restrepo Giraldo, Maria Clara Mendoza Arango (Hospital Universitario San Vicente Fundación).

Croatia: Edgar Domini, Robert Karlo, Jakov Mihanovic (Zadar General Hospital).

Egypt: Mohamed Youssef, Hossam Elfeki, Waleed Thabet, Aly Sanad, Gehad Tawfik, Ahmed Zaki, Noran Abdel-Hameed, Mohamed Mostafa, Muhammad Fathi Waleed Omar, Ahmed Ghanem, Emad Abdallah, Adel Denewar, Eman Emara, Eman Rashad, Ahmad Sakr, Rehab Elashry, Sameh Emile (Mansoura University Hospital); Toqa Khafagy, Sara Elhamouly, Arwa Elfarargy, Amna Mamdouh Mohamed, Ghada Saied Nagy, Abeer Esam, Eman Elwy, Aya Hammad, Salwa Khallaf, Eman Ibrahim, Ahmed Saidbadr, Ahmed Moustafa, Amany Eldosouky Mohammed, Mohammed Elgheriany, Eman Abdelmageed, Eman Abd Al Raouf, Esraa Samir Elbanby, Maha Elmasry, Mahitab Morsy Farahat, Eman Yahya Mansor, Eman Magdy Hegazy, Esraa Gamal, Heba Gamal, Hend Kandil, Doaa Maher Abdelrouf, Mohamed Moaty,(Menofiya University Hospital); Dina Gamal, Nada El-Sagheer, Mohamed Salah, Salma Magdy, Asmaa Salah, Ahmed Essam, Ahmed Ali, Mahmoud Badawy, Sara Ahmed, (Beni Suef University Hospital); Mazed Mohamed, Abdelrahman Assal, Mohamed Sleem, Mai Ebidy, Aly Abd Elrazek, Diaaaldin Zahran, Nourhan Adam, Mohamed Nazir, Adel B Hassanein, Ahmed Ismail, Amira Elsawy, Rana Mamdouh, Mohamed Mabrouk, Lopna Ahmed Mohamed Ahmed, Mohamed Hassab Alnaby, Eman Magdy, Manar Abd-Elmawla, Marwan Fahim, Bassant Mowafy, Moustafa Ibrahim Mahmoud, Meran Allam, Muhammad Alkelani, Noran Halim El Gendy, Mariam Saad Aboul-Naga, Reham Alaa El-Din, Alyaa Halim Elgendy, Mohamed Ismail, Mahmoud Shalaby, Aya Adel Elsharkawy, Mahmoud Elsayed Moghazy, Khaled Hesham Elbisomy, Hend Adel Gawad Shakshouk, Mohamed Fouad Hamed, Mai Mohamed Ebidy, Mostafa Abdelkader, Mohamed Karkeet (Alexandria Main University Hospital); Hayam Ahmed, Israa Adel, Mohammad Elsayed Omar, Mohamed Ibrahim, Omar Ghoneim, Omar Hesham, Shimaa Gamal, Karim Hilal, Omar Arafa, Sawsan Adel Awad,

Menatalla Salem, Fawzia Abdellatif Elsherif, Nourhan Elsabbagh, Moustafa R. Aboelsoud, Ahmed Hossam Eldin Fouad Rida, Amr Hossameldin, Ethar Hany, Yomna Hosny Asar, Nourhan Anwar, Mohamed Gadelkarim, Samar Abdelhady, Eman Mohamed Morshedy, Reham Saad, Nourhan Soliman, Mahmoud Salama (Alexandria Medical Research Institute); Eslam Ezzat, Arwa Mohamed, Arwa Ibrahim, Alaa Fergany, Sara Mohammed, Aya Reda, Yomna Allam, Hanan Adel Saad, Afnan Abdelfatah, Aya Mohamed Fathy, Ahmed El-Sehily, Esraa Abdalmageed Kasem, Ahmed Tarek Abdelbaset Hassan, Ahmed Rabeih Mohammed, Abdalla Gamal Saad, Yasmin Elfouly, Nesma Elfouly, Arij Ibrahim, Amr Hassaan, Mohammed Mustafa Mohammed, Ghada Elhoseny, Mohamed Magdy, Esraa Abd Elkhalek, Yehia Zakaria, Tarek Ezzat, Ali Abo El Dahab , Mohamed Kelany, Sara Arafa, Osama Mokhtar Mohamed Hassan, Nermin Mohamed Badwi, Ahmad Saber Sleem, Hussien Ahmed, Kholoud Abdelbadeai, Mohamed Abozed Abdullah (Faculty Of Medicine, Zagazig University); Muhammad Amsyar Auni Lokman, Suraya Bahar, Anan Rady Abdelazeam, Abdelrahman Adelshone, Muhammad Bin Hasnan, Athirah Zulkifli, Siti Nur Alia Kamarulzamil, Abdelaziz Elhendawy, Aliang Latif, Ahmad Bin Adnan, Shahadatul Shaharuddin, Aminah Hanum Haji Abdul Majid, Mahmoud Amreia, Dina Al-Marakby, Mahmoud Salma, Mohamad Jeffrey Bin Ismail, Elissa Rifhan Mohd Basir, Citra Dewi Mohd Ali, Aya Yehia Ata (Faculty Of Medicine, Tanta University); Maha Nasr, Asmaa Rezq, Ahmed Sheta, Sherif Tariq, Abd Elkhalek Sallam, Abdelrhman KZ Darwish, Sohaila Elmihy, Shady Elhadry, Ahmed Farag, Haidar Hajeh, Abdelaziz Abdelaal, Amro Aglan, Ahmed Zohair, Mahitab Essam, Omar Moussa, Esraa El-Gizawy, Mostafa Samy, Safia Ali, Esraa Elhalawany, Ahmed Ata, Mohamed El Halawany, Mohamed Nashat, Samar Soliman, Alaa Elazab, Mostada Samy, (El-Menshawy General Hospital); Mohamed A Abdelaziz, Khaled Ibrahim, Ahmed mohamed Ibrahim, Ammar Gado, Usama Hantour (Al-Hussein Hospital); Esraa Alm Eldeen, Mohamed Reda loaloa, Arwa Abouzaid, Mostafa Ahmed Bahaa Eldin, Eman Hashad, Fathy Sroor, Doaa Gamil, Eman Mahmoud Abdulhakeem, Mahmoud Zakaria, Fawzy Mohamed, Marwan Abubakr, Elsayed Ali, Hesham Magdy, Menna Tallah Ramadan, Mohamed Abdelaty Mohamed, Salma Mansour, Hager Abdul Aziz Amin, Ahmed Rabie Mohamed, Mahmoud Saami, Nada Ahmed Reda Elsayed, Adham Tarek, Sabry Mohy Eldeen Mahmoud, Islam Magdy El Sayed, Amira Reda, Martina Yusuf Shawky, Mohammed Mousa Salem, Shahinaz Alaa El-Din, Noha Abdullah Soliman, Muhammed Talaat, Shahinaz Alaael-Dein, Ahmed Abd Elmoen Elhusseiny, Noha Abdullah, Mohammed Elshaar, Aya AbdelFatah Ibraheem, Hager Abdulaziz, Mohammed Kamal Ismail, Mona Hamdy Madkor, Mohamed Abdelaty, Sara Mahmoud Abdel-Kader, Osama Mohamed Salah, (Benha Faculty Of Medicine); Mahmoud Eldafrawy, Ahmed Zaki Eldeeb, Mostafa Mahmoud Eid (October 6 University Hospital); Attia Attia, Khalid Salah El-Dien, Ayman Shwky (Bab El-Shareia University Hospital); Mohamed Adel Badenjki, Abdelrahman Soliman, Samaa Mahmoud Al Attar, (The Memorial Soaad Kafafi University Hospital); Farrag Sayed, Fahd Abdel Sabour, Mohammed G. Azizeldine, Muhammad Shawqi, Abdullah Hashim, Ahmed Aamer, Ahmed Mahmoud Abdelraouf, Mahmoud Abdelshakour, Amal Ibrahim, Basma Mahmoud, Mohamed Ali Mahmoud, Mostafa Qenawy, Ahmed M. Rashed, Ahmed Dahy, Marwa Sayed, Ahmed W. Shamsedine, Bakeer Mohamed, Ahmad Hasan, Mahmoud M. Saad, Khalil Abdul Bassit (Assiut University Hospital); Nadia Khalid Abd El-Latif, Nada Elzahed, Ahmed El Kashash, Nada Mohamed Bekhet, Sarah Hafez, Ahmed Gad, Mahmoud Elkhadragy Maher, Ahmed Abd Elsameea, Mohamed Hafez, Ahmad Sabe, Ataa Ahmed, Ahmed Shahine, Khaled Dawood, Shireen Gaafar, Reem Husseiny, Omnia Aboelmagd, Ahmed Soliman, Nourhan Mesbah, Hossam Emadeldin, Amgad Al Meligy, Amira Hassan Bekhet, Doaa Hasan, Khaled Alhady, Ahmad Khaled Sabe, Mahmoud A. Elnajjar, Majed Aboelella, Ward Hamsho, Ihab Hassan, Hala Saad, Galaleldin Abdelazim, Hend Mahmoud, Noha Wael, Ahmedali M Kandil, Ahmed Magdy, Shimaa Said Elkholy, Badr Eldin Adel, Kareem Dabbour, Saged Elsherbiney, Omar Mattar, Abdulshafi Khaled Abdrabou, Mohammed Yahia Mohamed Aly, Abdelrahman Geuoshy, Ahmedglal Elnagar, Saraibrahim Ahmed, Ibrahem Abdelmotaleb, Amr Ahmed Saleh, Manar Saeed, Shady Mahmoud, Badreldin Adel Tawfik, Samar Adel Ismail, Esraay Zakaria, Mariam O. Gad, Mohamed Salah Elhelbawy, Monica Bassem, Noha Maraie, Nourhan Medhat Elhadary, Nourhan Semeda, Shaza Rabie Mohamed, Hesham Mohammed Bakry, AA Essam (Kasr Al-Ainy Faculty Of Medicine, Cairo University); Dina Tarek,

Khlood Ashour, Alaa Elhadad, Abdulrahman Abdel-Aty, Ibrahim Rakha, Sara Mamdouh Matter, Rasha Abdelhamed, Omar Abdelkader, Ayat Hassaan, Yasmin Soliman, Amna Mohamed, Sara Ghanem, Sara Amr Mohamed Farouk, Eman Mohamed Ibrahim, Esraa El-Taher (Faculty Of Medicine Seuz Canal University); Merna Mostafa, Mohamed Fawzy Mahrous Badr, Rofida Elsemelawy, Aya El-Sawy, Ahmad Bakr, Ahmad Abdel Razaq Al Rafati (Smouha University Hospital);

Estonia: Sten Saar, Arvo Reinsoo, Peep Talving (The North Estonia Medical Centre);

Ethiopia: Nebyou Seyoum, Tewodros Worku, Agazi Fitsum, (Addis Ababa University, College Of Health Sciences, School Of Medicine);

Finland: Matti Tolonen, Ari Leppäniemi, Ville Sallinen (Helsinki University Hospital);

France: Benoît Parmentier, Matthieu Peycelon, Sabine Irtan (Trousseau Hospital, APHP); Sabrina Dardenne, Elsa Robert (GHICL); Betty Maillot, Etienne Courboin, Alexis Pierre Arnaud, Juliette Hascoet (CHU Rennes); Olivier Abbo, Amir Ait Kaci, Thomas Prudhomme (CHU Toulouse); Quentin Ballouhey, Céline Grosos, Laurent Fourcade (CHU Limoges); Tolg Cecilia, Colombani Jean-Francois, Francois-Coridon Helene (CHU Martinique); Xavier Delforge, Elodie Haraux (CHU Amiens Picardie); Bertrand Dousset, Roberto Schiavone, Sebastien Gaujoux, (Cochin - APHP); Jean-Baptiste Marret, Aurore Haffreingue, Julien Rod (CHU Caen); Mariette Renaux-Petel (CHU Rouen); Jean-François Lecompte, Jean Bréaud, Pauline Gastaldi (CHU Lenval Nice); Chouikh Taieb, Raquillet Claire, Echaieb Anis (Hopital Robert Ballanger, Paris); Nasir Bustangi, Manuel Lopez, Aurelien Scalabre (CHU De Saint Etienne); Maria Giovanna Grella (CHU Poitiers); Aurora Mariani, Guillaume Podevin, Françoise Schmitt (CHU Angers); Erik Hervieux, Aline Broch, Cecile Muller (Hopital Necker Enfants Malades, APHP).

Ghana: Stephen Tabiri, Anyomih Theophilus Teddy Kojo, Dickson Bandoh, Francis Abantanga, Martin Kyereh, Hamza Asumah, Eric Kofi Appiah, Paul Wondoh (Tamale Teaching Hospital); Adam Gyedu, Charles Dally, Kwabena Agbedinu, Michael Amoah, Abiboye Yifieyeh, (Kwame Nkrumah University of Science and Technology/Komfo Anokye Teaching Hospital); Frank Owusu (St. Particks Hospital); Mabel Amoako-Boateng, Makafui Dayie, Richmond Hagan, Sam Debrah, (Cape Coast Teaching Hosital); Micheal Ohene-Yeboah, Joe-Nat Clegg-Lampety, Victor Etwire, Jonathan Dakubo, Samuel Essoun, William Bonney, Hope Glover-Addy, Samuel Osei-Nketiah, Joachim Amoako, Niiarmah Adu-Aryee, William Appeadu-Mensah, Antoinette Bediako-Bowan, Florence Dedey (Korle Bu Teaching Hospital); Mattew Ekow, Emmanuel Akatibo, Musah Yakubu (Baptist Medical Center, Nalerigu); Hope Edem Kofi Kordorwu, Kwasi Asare-Bediako, Enoch Tackie (Keta Hospital District Hospital, Keta); Kenneth Aaniana, Emmanuel Acquah, Richard Opoku-Agyeman, Anthony Avoka, Kwasi Kusi, Kwame Maison, (Techiman Holy Family Hospital); Frank Enoch Gyamfi (Berekum Holy Family Hospital); Gandau Naa Barnabas, Saiba Abdul-Latif (Upper West Regional Hospital); Philip Taah Amoako (Samapa Government Hospital); Anthony Davor, Victor Dassah (Upper East Regional Hospital); Enoch Dagoe (St. Mary's Hospital); Prince Kwakyeafriyie (Essumejaman Sda Hospital, Dominase); Elliot Akoto, Eric Ackom, Ekow Mensah (Dormaa Presbyterian Hospital); Ebenezer Takyi Atkins, Christian Lari Coompson (Brongho-Ahafo Regional Hospital, Sunyani)

Greece: Nikolaos Ivros, Christoforos Ferousis, Vasileios Kalles, Christos Agalianos, Ioannis Kyriazanos, Christos Barkolias, Angelos Tselos, Georgios Tzikos, Evangelos Voulgaris (Naval And Veterans Hospital); Dimitrios Lytras, Athanasia Bamicha, Kyriakos Psarianos (Achillopoyleio General Hospital Of Volos); Anastasios Stefanopoulos (General Hospital Of Nafplio, Department Of Surgery); Ioannis Patoulias, Dimitrios Sfougaris, Ioannis Valioulis (G. Gennimatas Hospital); Dimitrios Balalis, Dimitrios Korkolis, Dimitrios K Manatakis (Saint Savvas Cancer Hospital); Georgios Kyrou, Georgios Karabelias, Iason-Antonios Papaskarlatos (General And Oncological Hospital Of Kifissia-Athens); Kolonia Konstantina, Nikolaos Zampitis, Stylianos Germanos (General Hospital Of Larissa); Aspasia Papailia, Theodosios Theodosopoulos, Georgios Gkiokas (2nd Dept Of Surgery, Aretaieion Hospital, National & Kapodistrian University Of Athens School Of Medicine); Magdalini Mitroudi, Christina Panteli, Thomas Feidantsis, Konstantinos Farmakis, (G. Gennimatas General Hospital); Dimitrios Kyziridis, Orestis

Ioannidis, Styliani Parpoudi (4th Surgical Department, Aristotle University Of Thessaloniki, General Hospital, Papanikolaou); Georgios Gemenetzis, Stavros Parasyris (Attikon University Hospital); Christos Anthoulakis, Nikolaos Nikoloudis, Michail Margaritis (Serres General Hospital);

Guatemala: Maria-Lorena Aguilera-Arevalo, Otto Coyoy-Gaitan, Javier Rosales (Hospital General San Juan De Dios); Luis Tale, Rafael Soley, Emmanuel Barrios (Juan Josè Arevalo Bermejo); Servio Tulio Torres Rodriguez, Carlos Paz Galvez, Danilo Herrera Cruz (Hospital San Vicente); Guillermo Sanchez Rosenberg, Alejandro Matheu, David Monterroso Cohen (Hospital Herrera Llerandi);

Haiti: Marie Paul, Angeline Charles (Hopital Universitaire De Mirebalais);

Hong Kong SAR, China: Justin Chak Yiu Lam, Man Hon Andrew Yeung, Chi Ying Jacquelyn Fok, Ka Hin Gabriel Li, Anthony Chuk-Him Lai, Yuk Hong Eric Cheung, Hong Yee Wong, Ka Wai Leung, Tien Seng Bryan Lee, Wai Him Lam, Weihei Dao, Stephanie Hiu-wai Kwok, Tsz-Yan Katie Chan, Yung Kok Ng, TWC Mak (Prince Of Wales Hospital); Qinyang Liu, Chi Chung Foo, James Yang, (University Of Hong Kong);

India: Basant Kumar, Ankur Bhatnagar, Vijaid Upadhyaya, (Sanjay Gandhi Post Graduate Institute Of Medical Sciences); Sunil Kumar (Excelcare Hospital); Uday Muddebihal, Wasim Dar, KC Janardha (Manipal Hospital); Philip Alexander, Neerav Aruldas, (Lady Willingdon Hospital);

Indonesia: Fidelis Jacklyn Adella, Anthonius Santoso Rulie, Ferdy Iskandar, Jonny Setiawan (Atma Jaya Hospital); Cicilia Viany Evajelista, Hani Natalie, Arlindawati Suyadi (Dr. Oen Surakarta Hospital); Rudy Gunawan, Herlin Karismaningtyas, Lusi Padma Sulistianingsih Mata, Ferry Fitriya Ayu Andika, Afifatun Hasanah, T Ariani Widiastini, Nurlaila Ayu Purwaningsih, Annisa Dewi Fitriana Mukin, Dina Faizatur Rahmah, Hazmi Dwinanda Nurqistan, Hasbi Maulana Arsyad, Novia Adhitama (Rsd Dr Soebandi); Wifanto Saditya Jeo, Nathania Sutandi, Audrey Clarissa, Phebe Anggita Gultom, Matthew Billy, Andreass Haloho, Radhian Amandito, Nadya Johanna, Felix Lee (Rsupn Cipto Mangunkusumo);

Ireland: Radin Mohd Nurrahman Radin Dorani, Martha Glynn, Mohammad Alherz, Wennweoi Goh, Haaris A. Shiwani, Lorraine Sproule, Kevin C. Conlon (Tallaght Hospital, Trinity College Dublin)

Israel: Miklosh Bala, Asaf Kedar (Hadassah Hebrew University Medical Center);

Italy: Luca Turati, Federica Bianco, Francesca Steccanella, (Treviglio Hospital); Gaetano Gallo, Mario Trompetto, Giuseppe Clerico (Department of Colorectal Surgery, S. Rita Clinic, Vercelli); Matteo Papandrea, Giuseppe Sammarco, Rosario Sacco (Department of Medical and Surgical Sciences, Policlinico Universitario Mater Domini Campus Salvatore Venuta, Catanzaro); Angelo Benevento, Francesco Pata, Luisa Giavarini (Sant'Antonio Abate Hospital, Gallarate); Mariano Cesare Giglio, Luigi Bucci, Gianluca Pagano, Viviana Sollazzo, Roberto Peltrini, Gaetano Luglio (Federico II University Of Naples); Arianna Birindelli, Salomone Di Saverio, Gregorio Tugnoli (Maggiore Hospital); Miguel Angel Paludi, Pietro Mingrone, Domenica Pata (Nicola Giannettasio Hospital, Rossano); Francesco Selvaggi, Lucio Selvaggi, Gianluca Pellino, Natale Di Martino (Universitá della Campania "Luigi Vanvitelli", Naples); Gianluca Curletti, Paolo Aonzo, Raffaele Galleano (Ospedale Santa Corona, Pietra Ligure (SV)); Stefano Berti, Elisa Francone, Silvia Boni, (S. Andrea Hospital, Poll-Asl 5, La Spezia), Laura Lorenzon, Annalisa lo Conte, Genoveffa Balducci (Sant'Andrea Hospital, Sapienza University of Rome); Gianmaria Confalonieri, Giovanni Pesenti (Azienda Ospedaliera Alessandro Manzoni); Laura Gavagna, Giorgio Vasquez, Simone Targa, Savino Occhionorelli, Dario Andreotti (Azienda Ospedaliero-Universitaria Di Ferrara); Giacomo Pata, Andrea Armellini, Deborah Chiesa (A.O. Spedali Civili Di Brescia); Fabrizio Aquilino, Nicola Chetta, Arcangelo Picciariello (Azienda Ospedaliero Universitaria Consorziale Policlinico Di Bari); Mohamed Abdelkhalek, Andrea Belli, Silvia De Franciscis (Istituto Nazionale Tumori Fondazione, Pascale-I.R.C.C.S.); Annamaria Bigaran, Alessandro Favero, Stefano M.M Basso (Azienda Per L'assistenza Sanitaria N. 5 Friuli Occidentale); Paola Salusso, Martina Perino, Sylvie Mochet, Diego Sasia, Francesco Riente, Marco Migliore (Azienda Sanitaria Ospedaliera San

Luigi Gonzaga); David Merlini, Silvia Basilicò, Carlo Corbellini (Ospedale Di Rho - ASST Rhodense); Veronica Lazzari, Yuri Macchitella, Luigi Bonavina (IRCCS Policlinico, University of Milano, San Donato); Daniele Angelieri, Diego Coletta, Federica Falaschi, Marco Catani, Claudia Reali, Mariastella Malavenda, Celeste Del Basso, Sergio Ribaldi, Massimo Coletti, Andrea Natili, Norma Depalma, Immacolata Iannone, Angelo Antoniozzi, Davide Rossi (Policlinico Umberto I Emergency Surgery Department); Daniele Gui, Gerardo Perrotta, Matteo Ripa, Francesco Ruben Giardino, Maurizio Foco, (Fondazione Policlinico Universitario Agostino Gemelli); Erika Vicario, Federico Coccolini, Luca Ansaloni, Gabriela Elisa Nita (AO Papa Giovanni XXIII); Nicoletta Leone, Andrea Bondurri, Anna Maffioli (Ospedale Sacco); Andrea Simioni, Davide De Boni, Sandro Pasquali (IOV - Istituto Oncologico Veneto); Elena Goldin, Elena Vendramin, Eleonora Ciccioli (Azienda Ospedaliera di Padova); Umberto Tedeschi, Luca Bortolasi, Paola Violi, Tommaso Campagnaro, Simone Conci, Giovanni Lazzari, Calogero Iacono, Alfredo Gulielmi, Serena Manfreda (Azienda Ospedaliera Universitaria Integrata di Verona); Anna Rinaldi, Maria Novella Ringressi, Beatrice Brunoni (Azienda Ospedaliera Universitaria Careggi); Giuseppe Salamone, Mirko Mangiapane, Paolino De Marco, Antonella La Brocca, Roberta Tutino, Vania Silvestri, Leo Licari, Tommaso Fontana, Nicolò Falco, Gianfranco Cocorullo, (Policlinico Paolo Giaccone di Palermo); Mostafa Shalaby, Pierpaolo Sileri, Claudio Arcudi (Policlinico Tor Vergata Hospital, Rome)

Jordan: Isam Bsisu, Khaled Aljboor, Lana Abusalem, Aseel Alnusairat, Ahmad Qaissieh, Emad Al-Dakka, Ali Ababneh, Oday Halhouli (Jordan University Hospital);

Kenya: Taha Yusufali, Hussein Mohammed (Kenyatta National Hospital); Justus Lando, Robert Parker, Wairimu Ndegwa (Tenwek Hospital);

Lithuania: Mantas Jokubauskas, Jolanta Gribauskaite, Donatas Venskutonis (Lithuanian University Of Health Sciences); Justas Kuliavas, Audrius Dulskas, Narimantas E. Samalavicius (Klaipeda University Hospital, National Cancer Institute); Kristijonas Jasaitis, Audrius Parseliunas, Viktorija Nevieraite, Margarita Montrimaite, Evelina Slapelyte, Edvinas Dainius, Romualdas Riauka, Zilvinas Dambrauskas, Andrejus Subocius, Linas Venclauskas, Antanas Gulbinas, Saulius Bradulskis, Simona Kasputyte, Deimante Mikuckyte, Mindaugas Kiudelis, Justas Zilinskas, Tomas Jankus, Steponas Petrikenas (Lithuanian University of Health Sciences); Matas Pažuskis, Zigmantas Urniežius, Mantas Vilčinskas (Republican Hospital of Kaunas); Vincas Jonas Banaitis, Vytautas Gaižauskas, Edvard Grisin, Povilas Mazrimas, Rokas Rackauskas, Mantas Drungilas, Karolis Lagunavicius, Vytautas Lipnickas, Dovilѐ Majauskyté, Valdemaras Jotautas, Tomas Abaliksta, Laimonas Uščinas, Gintaras Simutis, Adomas Ladukas, Donatas Danys, Erikas Laugzemys, Saulius Mikalauskas, Tomas Poškus, Elena Zdanyte Sruogiene, (Vilnius University Hospital); Petras Višinskas, Reda Žilinskienė, Deividas Dragatas (Hospital Of Jonava); Andrius Burmistrovas, Zygimantas Tverskis (Taurage Hospital); Arturas Vaicius, Ruta Mazelyte, Antanas Zadoroznas (Viesoji Istaiga Rokiskio Rajono Ligonine); Nerijus Kaselis, Greta Žiubrytė, (Republican Hospital Of Klaipeda);

Madagascar: Finaritra Casimir Fleur Prudence Rahantasoa, Luc Hervé Samison, Fanjandrainy Rasoaherinomenjanahary, Todisoa Emmanuella Christina Tolotra (Joseph Ravoahangy Andrianavalona Hospital);

Malawi: Cornelius Mukuzunga, Vanessa Msosa, Chimwemwe Kwatiwani, Nelson Msiska (Kamuzu Central Hospital);

Malaysia: Feng Yih Chai, Siti Mohd Desa Asilah, Khuzaimah Zahid Syibrah (Hospital Keningau); Pui Xin Chin, Afizah Salleh, Nur Zulaika Riswan (Kajang Hospital); April Camilla Roslani, Hoong-Yin Chong, Nora Abdul Aziz, Keat-Seong Poh, Chu-Ann Chai, Sandip Kumar (University Malaya Medical Centre); Mustafa Mohammed Taher, Nik Ritza Kosai, Dayang Nita Abdul Aziz, Reynu Rajan (Universiti Kebangsaan Malaysia Medical Centre UKMMC); Rokayah Julaihi, Durvesh Lacthman Jethwani, Muhammad Taqiyuddin Yahaya, Nik Azim Nik Abdullah, Susan Wndy Mathew, Kuet Jun Chung, Milaksh Kumar Nirumal, R. Goh Ern Tze, Syed Abdul Wahhab Eusoffee Wan Ali (Sarawak General Hospital); Yiing Yee Gan, Jesse Ron Swire Ting (Hospital Sibu); Samuel S. Y. Sii, Kean Leong Koay, Yi Koon Tan, Alvin Ee Zhiun Cheah, Chui Yee Wong, Tuan Nur'Azmah

Tuan Mat, Crystal Yern Nee Chow, Prisca A.L. Har, Yishan Der (Hospital Sultanah Aminah); Yong Yong Tew, Fitjerald Henry, Xinwei Low (Selayang Hospital); Ya Theng Neo, Hian Ee Heng, Shu Ning Kong, Cheewei Gan, Yi Ting Mok, Yee Wen Tan, Kandasami Palayan, Mahadevan Deva Tata, Yih Jeng Cheong (Hospital Tuanku Ja'afar); Kuhaendran Gunaseelan, Wan Nurul 'Ain Wan Mohd Nasir, Pigeneswaren Yoganathan, (Hospital Keningau); Eu Xian Lee, Jian Er Saw, Li Jing Yeang, Pei Ying Koh, Shyang Yee Lim, Shuang Yi Teo (Hospital Pulau Pinang / Penang Medical College);

Malta: Nicole Grech, Daniela Magri, Kristina Cassar, Christine Mizzi, Malcolm Falzon, Nihaal Shaikh, Ruth Scicluna, Stefan Zammit, Elaine Borg, Sean Mizzi, Svetlana Doris Brincat, Thelma Tembo, Vu Thanh Hien Le, Tara Grima, Keith Sammut, Kurt Carabott, Alexia Farrugia, Ciskje Zarb, Andre Navarro, Thea Dimech, Georgette Marie Camilleri, Isaac Bertuello, Jeffrey Dalli, Karl Bonavia (Mater Dei Hospital);

Mexico: Samantha Corro-Diaz, Marisol Manriquez-Reyes, Antonio Ramos-De la Medina (Hospital Español de Veracruz);

Morocco: Amina Abdelhamid, Abdelmalek Hrora, Sarah Benammi, Houda Bachri, Meryem Abbouch, Khaoula Boukhal, Redouane Mammar Bennai, Abdelkader Belkouchi, Mohamed Sobhi Jabal, Chaymae Benyaiche (IBN Sina Hospital)

Netherlands: Maarten Vermaas, Lucia Duinhouwer, (Ijsselland Hospital)

Nicaragua: Javier Pastora, Greta Wood, Maria Soledad Merlo (Hospital Escuela Oscar Danilo Rosales Arguello);

Nigeria: Akinlabi Ajao, Omobolaji Ayandipo, Taiwo Lawal, Abdussemiu Abdurrazzaaq, (University College Hospital, Ibadan); Muslimat Alada, Abdulrasheed Nasir, James Adeniran, Olufemi Habeeb, Ademola Popoola, Ademola Adeyeye (University Of Ilorin Teaching Hospital,Ilorin); Ademola Adebanjo, Opeoluwa Adesanya, Adewale Adeniyi (Federal Medical Centre, Abeokuta,); Henry Mendel, Bashir Bello, Umar Muktar (Usmanu Danfodiyo University Teaching Hospital);); Adedapo Osinowo, Thomas Olagboyega Olajide, Oyindamola Oshati, George Ihediwa, Babajide Adenekan, Victor Nwinee, Felix Alakaloko, Adesoji Ademuyiwa, Olumide Elebute, Abdulrazzaq Lawal, Chris Bode, Mojolaoluwa Olugbemi (Lagos University Teaching Hospital); Alaba Adesina, Olubukola Faturoti, Oluwatomi Odutola, Oluwaseyi Adebola, Clement Onuoha, Ogechukwu Taiwo (Babcock University Teaching Hospital); Omolara Williams, Fatai Balogun, Olalekan Ajai, Mobolaji Oludara, Iloba Njokanma, Roland Osuoji (Lagos State University Teaching Hospital); Stephen Kache, Jonathan Ajah, Jerry Makama (Barau Dikko Teaching Hospital, Kaduna State University, Kaduna); Ahmed Adamu, Suleiman Baba, Mohammad Aliyu, Shamsudeen Aliyu, Yahaya Ukwenya, Halima Aliyu, Tunde Sholadoye, Muhammad Daniyan, Oluseyi Ogunsua (Ahmadu Bello University Teaching Hospital Zaria); Lofty-John Anyanwu, Abdurrahaman Sheshe, Aminu Mohammad (Aminu Kano Teaching Hospital); Samson Olori, Philip Mshelbwala, Babatunde Odeyemi, Garba Samson, Oyediran Kehinde Timothy, Sani Ali Samuel (University Of Abuja Teaching Hospital,); Anthony Ajiboye, Ademola Adeyeye, Isaac Amole, Olajide Abiola, Akin Olaolorun (Bowen University Teaching Hospital);

Norway: Kjetil Søreide, Torhild Veen, Arezo Kanani, Kristian Styles, Ragnar Herikstad, Johannes Wiik Larsen, Jon Arne Søreide (Stavanger University Hospital); Elisabeth Jensen, Mads Gran, Eirik Kjus Aahlin (University Hospital Of Northern Norway); Tina Gaarder, Peter Wiel Monrad-Hansen, Pål Aksel Næss (Oslo University Hospital); Giedrius Lauzikas, Joachim Wiborg, Silje Holte (Sykehuset Telemark HF); Knut Magne Augestad, Gurpreet Singh Banipal, Michela Monteleone, Thomas Tetens Moe, Johannes Kurt Schultz (Akershus University Hospital);

Palestine: Taher Al-taher, Ayah Hamdan, Ayman Salman, Rana Saadeh, Aseel Musleh, Dana Jaradat, Soha Abushamleh, Sakhaa Hanoun, Amjad Abu Qumbos, Aseel Hamarshi, Ayman And Taher (Al Makassed Islamic Charitable Society Hospital, Jerusalem); Israa Qawasmi, Khalid Qurie, Marwa Altarayra, Mohammad Ghannam, Alaa Shaheen, Azher Herebat (Alia Governmental Hospital); Aram Abdelhaq, Ahmad Shalabi, Maram Abu-toyour, Fatema Asi, Ala Shamasneh, Anwar Atiyeh, Mousa Mustafa, Rula Zaa'treh, Majd Dabboor (Palestine Medical Complex); Enas Alaloul, Heba Baraka, Jehad Meqbil, Alaa Al-Buhaisi, Mohamedraed Elshami, Samah Afana, Sahar Jaber, Said Alyacoubi, Yousef Abuowda (Islamic University of Gaza

Medical School, European Gaza Hospital & Shifa Hospital); Tasneem Idress, Eman Abuqwaider (Mizan hospital); Sara Al-saqqa, Alaa Bowabsak, Alaa El Jamassi, Doaa Hasanain, Hadeel Al-farram, Maram Salah, Aya Firwana, Marwa Hamdan, Israa Awad (Al-Shifa Hospital); Ahmad Ashour, Fayez Elian Al Barrawi (Bit Hanoun Hospital); Ahmed Al-khatib, Maha Al-faqawi, Mohamed Fares (Nasser Hospital); Amjad Elmashala, Mohammad Adawi, Ihdaa Adawi (Beit Jala Governmental Hospital); Reem Khreishi, Rose Khreishi (Martyr Thabet Govermental hospital, Tulkarem); Ahmad ashour, Ahed Ghaben (Indonesian Hospital, Gaza)

Pakistan: Najwa Nadeem, Muhammad Saqlain (Allied Hospital, Faisalabad); Jibran Abbasy, Abdul Rehman Alvi, Tanzeela Gala, Noman Shahzad (Aga Khan University); Kamran Faisal Bhopal, Zainab Iftikhar, Muhammad Talha Butt, Syed Asaat ul Razi, Asdaq Ahmed, Ali Khan Niazi (Bahawal Victoria Hospital); Ibrahim Raza, Fatima Baluch, Ahmed Raza, Ahmad Bani-Sadar, Ahmad Uzair Qureshi, Muhammad Adil, Awais Raza, (King Edward Medical University, Mayo Hospital, Lahore); Mahnoor Javaid, Muhammad Waqar, Maryam Ali Khan (CMH Lahore Medical And Dental College); Mohammad Mohsin Arshad, Mohammadasim Amjad (Nishtar Medical College And Hospital);

Paraguay: Gustavo Miguel Machain Vega, Jorge Torres Cardozo, Marcelo O´Higgins Roche, Gustavo Rodolfo Pertersen Servin, Helmut Alfredo Segovia Lohse, Larissa Ines Páez Lopez, Ramón Augusto Melo Cardozo (Hospital de Clínicas, II Cátedra de Clínica Quirúrgica, Universidad Nacional de Asunción)

Peru: Fernando Espinoza, Angel David Pérez Rojas, Diana Sanchez, Camila Sanchez Samaniego, Shalon Guevara Torres, Alexander Canta Calua, Cesar Razuri, Nadia Ortiz, Xianelle Rodriguez, Nahilia Carrasco, Fridiz Saravia, Hector Shibao Miyasato, María Valcarcel-Saldaña, Ysabel Esthefany Alejos Bermúdez, Juan Carpio, Walter Ruiz Panez, Pedro Angel Toribio Orbegozo, (Hospital Nacional Arzopispo Loayza); Carolina Guzmán Dueñas, Kevin Turpo Espinoza, Ana Maria Sandoval Barrantes, Jorge Armando Chungui Bravo, Sebastian Shu, Lorena Fuentes-Rivera, Carmen Fernández, Diego Romani, Bárbara Málaga, Joselyn Ye (Hospital Cayetano Heredia); Ricardo Velasquez, Jannin Salcedo (Clínica De Especialidades Médicas); Ana Lucia Contreras-Vergara, Angelica Genoveva Vergara Mejia, Maria Soledad Gonzales Montejo (Hospital Nacional Guillermo Almenara Irigoyen); Marilia Del Carmen Escalante Salas, Willy Alcca Ticona, Marvin Vargas, George Christian Manrique Sila, Robinson Mas, Arazzelly del Pilar Paucar (Hospital Regional De Ayacucho); Armando José Román Velásquez, Alina Robledo-Rabanal, Ludwing Alexander Zeta Solis, (Hospital III José Cayetano Heredia); Kenny Turpo Espinoza (Hospital Nacional Maria Auxiliadora); José Luis Hamasaki Hamaguchi, Erick Samuel Florez Farfan, Linda Alvi Madrid Barrientos, Juan Jaime Herrera Matta (Hospital De Policia);

Philippines: John Jemuel V. Mora, Menold Archee P. Redota, Manuel Francisco Roxas, Maria Jesusa B. Maño, (The Medical City); Marie Dione Parreno-Sacdalan, Marie Carmela Lapitan, Christel Leanne Almanon (Department Of Surgery, Philippine General Hospital, University Of The Philippines Manila);

Poland: Maciej Walędziak, Rafał Roszkowski, Michał Janik (Department Of General, Oncological, Metabolic And Thoracic Surgery, Military Institute Of Medicine, Warsaw); Anna Lasek, Piotr Major, Dorota Radkowiak, Mateusz Rubinkiewicz (2nd Department Of Surgery, Jagiellonian University Medical College);

Portugal: Cristina Fernandes, Jose Costa-Maia, Renato Melo (Centro Hospitalar De São João);

Romania: Liviu Muntean, Aurel Sandu Mironescu, Lucian Corneliu Vida (Spitalul Clinic De Copii Brasov); Amar Kourdouli, Mariuca Popa (Spital Judetean De Urgenta Din Craiova); Hogea Mircea (Spitalul Clinic Judetean Brasov); Mihaela Vartic, Bogdan Diaconescu, Matei Razvan Bratu, Ionut Negoi, Mircea Beuran, Cezar Ciubotaru (Emergency Hospital of Bucharest);

Rwanda: J.C Allen Ingabire, Alphonse Zeta Mutabazi, Norbert Uzabumwana (University Teaching Hospital Of Kigali); Dieudonne Duhoranenayo (Kibungo Hospital);

San Marino: Elio Jovine, Nicola Zanini, Giovanni Landolfo (San Marino State Hospital);

Saudi Arabia: Murad Aljiffry, Faisal Idris, Mohammed Saleh A. Alghamdi, Ashraf Maghrabi, Abdulmalik Altaf, Aroub Alkaaki, Ahmad Khoja, Abrar Nawawi, Sondos Turkustani, (Department of Surgery, Faculty of Medicine, King Abdulaziz University Hospital, Jeddah) Eyad Khalifah, Ahmad Gudal, Adel Albiety, Sarah Sahel, Reham Alshareef, Mohammed Najjar (Department of Surgery, King Abdulaziz University Hospital and Oncology Center, Jeddah) Ahmed Alzahrani, Ahmed Alghamdi, Wedyan Alhazmi, (King Fahad Hospital, Jeddah) Ghiath Al Saied, Mohammed Alamoudi, Muhammed Masood Riaz (King Fahad Medical City, Riyadh); Mazen Hassanain, Basmah Alhassan, Abdullah Altamimi, Reem Alyahya, Norah Al Subaie, Fatema Al Bastawis, Afnan Altamimi, Thamer Nouh, Roaa Khan, (King Khaled University Hospital);

Serbia: Milan Radojkovic, Ljiljana Jeremic, Milica Nestorovic (Clinic For General Surgery, Clinical Center Nis);

Singapore: Jia Hao Law, Keith Say Kwang Tan, Ryan Choon Kiat Tan, Joel Kin Tan, Lau Wen Liang Joel, Bettina Lieske, Xue Wei Chan, Faith Qi Hui Leong, Choon Seng Chong, Sharon Koh, Kai Yin Lee, Kuok Chung Lee (National University Hospital)

South Africa: Kent Pluke, Britta Dedekind, Puyearashid Nashidengo, Mark Ian Hampton (Victoria Hospital Wynberg); Johanna Joosten, Sanju Sobnach, Liana Roodt, Anthony Sander, James Pape, Richard Spence (Groote Schuur); Niveshni Maistry (Charlotte Maxeke Johannesburg Academic Hospital); Phumudzo Ndwambi, Kamau Kinandu, Myint Tun (Leratong Hospital); Frederick Du Toit, Quinn Ellison, Sule Burger, DC Grobler, Lawrence Bongani Khulu (Tembisa Tertiary Provincial Hospital); Rachel Moore, Vicky Jennings, Astrid Leusink (Chris Hani Baragwanath Academic Hospital); Nazmie Kariem, Juan Gouws, Kathryn Chu, Heather Bougard, Fazlin Noor, Angela Dell (New Somerset Hospital); Sarah Rayne, Stephanie Van Straten, (Helen Joseph Hospital, University Of Witwatersrand); Arvin Khamajeet, Serge Kapenda Tshisola, Kalangu Kabongo (Stanger Hospital); Victor Kong (Edendale Hospital); Yoshan Moodley, Frank Anderson, Thandinkosi Madiba (Inkosi Albert Luthuli Central Hospital); Flip du Plooy (Mediclinic Potchefstroom); Leila Hartford, Gareth Chilton, Parveen Karjiker (Mitchell's Plain District Hospital); Matlou Ernest Mabitsela, Sibongile Ruth Ndlovu (Dr George Mukhari Academic Hospital); Maria Badicel, Robert Jaich (Milpark Hospital)

Spain: Jaime Ruiz-Tovar (University Hospital Rey Juan Carlos); Luis Garcia-Florez, Jorge L. Otero-Díez, Virginia Ramos Pérez, Nuria Aguado Suárez (Hospital Universitario San Agustín); Javier Minguez García, Sara Corral Moreno, Maria Vicenta Collado , Virginia Jiménez Carneros, Javier García Septiem (Hospital Universitario de Getafe); Mariana Gonzalez, Antonio Picardo, Enrique Esteban, Esther Ferrero, Irene Ortega, (Infanta Sofía University Hospital); Eloy Espin-Basany, Ruth Blanco-Colino, Valeria Andriola (Hospital Valle De Hebron); Lorena Solar García, Elisa Contreras, Carmen García Bernardo, Janet Pagnozzi, Sandra Sanz, Alberto Miyar de León, Asnel Dorismé, Joseluis Rodicio, Aida Suarez, Jessica Stuva, Tamara Diaz Vico (Central University Hospital Of Asturias); Laura Fernandez-Vega, Carla Soldevila-Verdeguer, Fatima Sena-Ruiz, Natalia Pujol-Cano, Paula Diaz-Jover, José Maria Garcia-Perez, Juan Jose Segura-Sampedro, Cristina Pineño-Flores, David Ambrona-Zafra, Andrea Craus-Miguel, Patricia Jimenez-Morillas, Angela Mazzella (Hospital Universitario Son Espases);

Sri Lanka: A.B Jayathilake, S.P.B Thalgaspitiya, L.S. Wijayarathna, P.M.S.N. Wimalge (University Surgical Unit, Teaching Hospital Anuradhapura);

St. Kitts And Nevis: Hakeem Ayomi Sanni, Aliyu Ndajiwo, Ogheneochuko Okenabirhie (Joseph N France Hospital)

Sudan: Anmar Homeida, Abobaker Younis, Omer Abdelbagi Omer, Mustafa Abdulaziz, Ali Mussad, Ali Adam (University of Gezira);

Sweden: Yucel Cengiz, Ida Björklund, Sandra Ahlqvist, Sandra Ahlqvist, (Sundsvall Hospital); Anders Thorell, Fredrik Wogensen (Ersta Hospital); Arestis Sokratous, Michaela Breistrand (Mora Hospital); Hildur Thorarinsdottir (Helsingborgs Lasarett); Johanna Sigurdadottir, Maziar Nikberg, Abbas Chabok (Västmanlands Hospital Västerås); Maria Hjertberg (Department Of Surgery And Department Of Clinical And Experimental Medicine, Linköping University, Norrköping, Sweden); Peter Elbe, Deborah Saraste, Wiktor Rutkowski, Louise Forlin (Karolinska Universitetssjukhuset, Solna); Karoliina Niska, Malin

Sund (Umea University Hospital)

Switzerland: Dennis Oswald, Georgios Peros, Rafael Bluelle, Katharina Reinisch, Daniel Frey, Adrian Palma (Gzo Spital Wetzikon); Dimitri Aristotle Raptis, Lucius Zumbühl, Markus Zuber (Kantonsspital Olten); Roger Schmid, Gabriela Werder (Buergerspital Solothurn); Antonio Nocito, Alexandra Gerosa, Silke Mahanty (Kantonsspital Baden); Lukas Werner Widmer, Julia Müller, Alissa Gübeli (Hospital Davos); Grzegorz Zuk (Gzo Spital Wetzikon);

Turkey: Osman Bilgin Gulcicek, Yuksel Altinel, Talar Vartanoglu, (Bagcilar Research And Training Hospital); Emin Kose, Servet Rustu Karahan, Mehmet Can Aydin (Okmeydanı Training And Research Hospital); Nuri Alper Sahbaz, Ilkay Halicioglu, Halil Alis (Bakirkoy Dr. Sadi Konuk Training And Research Hospital); Ipek Sapci, Can Adıyaman, Ahmet Murat Pektaş, Turgut Bora Cengiz, Ilkan Tansoker, Vedatcan Işler, Muazzez Cevik, Deniz Mutlu, Volkan Ozben, Berk Baris Ozmen, Sefa Bayram, Sinem Yolcu, Berna Buse Kobal, Ömer Faruk Toto, Haluk Cem Çakaloğlu (Acibadem University School of Medicine, Atakent Hospital); Kagan Karabulut, Vahit Mutlu, Bahar Busra Ozkan (Ondokuz Mayis University Medical Faculty); Saban Celik, Anil Semiz, Selim Bodur, Enisburak Gül, Busra Murutoglu, Reyyan Yildirim, Bahadir Emre Baki, Ekin Arslan, Ali Guner, Kadir Tomas (Karadeniz Technical University Faculty Of Medicine);

United Kingdom: Nathan Walker, Nikhita Shrimanker, Michael Stoddart, Simon Cole (Royal United Hospital Bath); Ryan Breslin, Ravi Srinivasan (Blackpool Victoria Hospital); Mohamed Elshaer, Kristina Hunter, Ahmed Al-Bahrani (Watford General Hospital); Ignatius Liew, Nora Grace Mairs, Alistair Rocke, Lachlan Dick, Mobeen Qureshi (Inverclyde Royal Hospital); Debkumar Chowdhury (University Hospital Ayr); Naomi Wright, Clare Skerritt, Dorothy Kufeji (Guy's And St. Thomas' Hospitals); Adrienne Ho, Tharindra Dissanayake, Athula Tennakoon, Wadah Ali, (Pilgrim Hospital, United Lincolnshire Hospitals NHS Trust); Shujing Jane Lim, Charlene Tan, Stephen O'Neill, Catrin Jones (Victoria Hospital Kirkcaldy); Stephen Knight, Dima Nassif, Abhishek Sharma (Perth Royal Infirmary); Oliver Warren, Rebecca White, Aia Mehdi, Nathan Post, Eliana Kalakouti, Enkhbat Dashnyam, Frederick Stourton (Chelsea And Westminster Hospital); Ioannis Mykoniatis, Chelise Currow, (Northampton General Hospital); Francisca Wong, Ashish Gupta, Veeranna Shatkar (Queen's Hospital, BHR University Hospitals NHS Trust); Joshua Luck, Suraj Kadiwar, Alexander Smedley (North Middlesex University Hospital); Rebecca Wakefield, Philip Herrod, James Blackwell, Jonathan Lund, (Royal Derby Hospital); Fraser Cohen, Ashwath Bandi, Stefano Giuliani (St George's Hospital); Giles Bond-Smith, Theodore Pezas, Neda Farhangmehr, Tomas Urbonas, Miklos Perenyei (John Radcliffe Hospital, Oxford); Philip Ireland, Natalie Blencowe, Kirk Bowling, David Bunting (Gloucestershire Royal Hospital); Lydia Longstaff, Neil Smart, Kenneth Keogh (Royal Devon & Exeter Hospital); Hyunjin Jeon, Muhammad Rafaih Iqbal, Shivun Khosla, Anna Jeffery, James Perera (Maidstone & Tunbridge Wells NHS Trust); Ella Teasdale (Western Isles Hospital); Ahmad Aboelkassem Ibrahem, Tariq Alhammali, Yahya Salama (Kettering General Hospital NHS Trust); Rakan Kabariti, Shaun Oram (Nevill Hall Hospital); Thomas Kidd, Fraser Cullen, Christopher Owen, Michael Wilson, Seehui Chiu, Hannah Sarafilovic, (Ninewells Hospital); Jennifer Ploski, Elizabeth Evans, Athar Abbas, Sylvia Kamya, Norzawani Ishak, Carly Bisset, Cedar Andress, Ye Ru Chin (Royal Alexandra Hospital, Paisley); Priya Patel, David Evans (University Hospital, Wales); Anna Jeffery, James Perera (Maistone and Tunbridge Wells NHS Trust); Aidan Haslegrave, Adam Boggon, Kirsten Laurie, Katie Connor, Thomas Mann (Borders General Hospital); Dmitri Nepogodiev, Anahita Mansuri, Rachel Davies, Ewen Griffiths (University Hospitals Birmingham NHS Trust); Aized Raza Shahbaz, Calvin Eng, Farhat Din, Ariadne L'Heveder, Esther H.G. Park, Ramanish Ravishankar, Kirsten McIntosh, Jih Dar Yau, Luke Chan, Susan McGarvie (Western General Hospital, Edinburgh); Lingshan Tang, Hui Lim, Suhhuey Yap, Jay Park, Zhan Herr Ng, Shahrukh Mirza, Yun Lin Ang, Luke Walls, Ella Teasdale, Chloe Roy, Simon Paterson-Brown, Julian Camilleri-Brennan, Kenneth Mclean, Michelle S D'Souza, Savva Pronin, David Ewart Henshall, Eunice Zuling Ter (Royal Infirmary Of Edinburgh); Dina Fouad, Ashish Minocha (Norfolk And Norwich University Hospital); William English, Catrin Morgan, Dominic Townsend, Laura Maciejec, Shareef Mahdi, Onyinye Akpenyi, Elisabeth Hall, Hanaan Caydiid, Zakaria Rob,

Tom Abbott, Hew D Torrance (The Royal London Hospital); Gareth Irwin, Robin Johnston (Ulster Hospital Dundonald); Mohammed Akil Gani, Gianpiero Gravante (Leicester Royal Infirmary); Shivanchan Rajmohan, Kiran Majid, Shiva Dindyal, Christopher Smith (West Middlesex University Hospital); Madanmohan Palliyil, Sanjay Patel, Luke Nicholson, Neil Harvey, Katie Baillie, Sam Shillito, Suzanne Kershaw, Rebecca Bamford, Peter Orton (Stockport NHS Foundation Trust); Elke Reunis, Robert Tyler, Wai Cheong Soon (Good Hope Hospital); Guled M. Jama, Dharminder Dhillon, Khyati Patel (Walsall Manor Hospital, Walsall); Shayanthan Nanthakumaran, Rachel Heard, Kar Yan Chen (Aberdeen Royal Infirmary); Behrad Barmayehvar, Uttaran Datta, Sivesh K Kamarajah, Sharad Karandikar (Heartlands Hospital); Sobhana Iftekhar Tani (Nottingham University Hospital NHS Trust); Eimear Monaghan, Philippa Donnelly, Michael Walker (Raigmore Hospital); Jehangirshaw Parakh, Sarah Blacker, Anil Kaul (Whiston Hospital); Arjun Paramasivan (Darlington Memorial Hospital); Sameh Farag, Ashrafun Nessa, Salwa Awadallah (Worthing Hospital, Western Sussex Hospital NHS Foundation Trust); Jieqi Lim, James Chean Khun Ng (Queen Elizabeth University Hospital, Glasgow);

United States: Katherine Gash, Ravi P. Kiran, Alice Murray (New York Presbyterian Hospital / Columbia University Medical Center); Eric Etchill, Mohini Dasari, Juan Puyana (University Of Pittsburgh Medical Center - Presbyterian); Nadeem Haddad, Martin Zielinski, Asad Choudhry (Mayo Clinic); Celeste Caliman, Mieshia Beamon, Therese Duane (John Peter Smith Hospital); Ragavan Narayanan, Mamta Swaroop (Northwestern Memorial Hospital / Northwestern University); Jonathan Myers, Rebecca Deal, Erik Schadde (Rush University Medical Centre); Mark Hemmila, Lena Napolitano, Kathleen To (University Of Michigan Medical Center)

Zambia: Alex Makupe, Joseph Musowoya, Mayaba Maimbo (Ndola Central Hospital); Niels Van Der Naald, Dayson Kumwenda, Alex Reece-Smith, Kars Otten, Anna Verbeek, Marloes Prins (St Francis Mission Hospital)

Data Validators:

Argentina: Alibeth Andres Baquero Suarez (Simplemente Evita), Ruben Balmaceda (Hospital Lagomaggiore);

Barbados: Chelsea Deane (Queen Elizabeth Hospital);

Croatia: Emilio Dijan (Zadar General Hospital);

Egypt: Mahmoud Elfiky (Kasr Al Ainy Faculty of Medicine, Cairo University);

Finland: Laura Koskenvuo (Helsinki University Hospital);

France: Aurore Thollot (CHU Poitiers), Bernard Limoges (CHU Limoges), Carmen Capito (Hopital Necker Enfants Malades, APHP), Challine Alexandre (Hopital Cochin, APHP), Henri Kotobi (Trousseau Hospital, APHP), Julien Leroux (CHU Rouen), Julien Rod (CHU Caen), Kalitha Pinnagoda (CHU Toulouse), Nicolas Henric (CHU Angers), Olivier Azzis (CHU Rennes), Olivier Rosello (CHU Nice), Poddevin Francois (GHICL), Sara Etienne (CHU Saint Etienne); Philippe Buisson (CHU Amiens Picardie), Sophian Hmila (Hopital Robert Ballanger, Paris);

Ghana: Joe-Nat Clegg-Lamptey (Korle Bu Teaching Hospital), Osman Imoro (Baptist Medical Centre), Owusu Emmanuel Abem (Komfo Anokye Teaching Hospital), Paul Wondoh (Upper West Regional Hospital);

Greece: Dimitrios Papageorgiou (Naval And Veterans Hospital Of Athens), Vasiliki Soulou (Anticancer Hospital Of Athens Agios Savvas);

Guatemala: Sabrina Asturias (Hospital Herrera Llerandi Amedesgua), Lenin Peña (Hospital General San Juan De Dios);

India: Basant Kumar (Sanjay Gandhi Post Graduate Institute Of Medical College Lucknow);

Ireland: Donal B O'Connor (Tallaght Hospital, Trinity College Dublin);

Italy: Alberto Realis Luc (Santa Rita Clinic, Vercelli), Alfio Alessandro Russo (Treviglio Hospital), Andrea Ruzzenente (Azienda Ospedaliera Universitaria Integrata di Verona), Antonio Taddei (Azienda Ospedaliera Universitaria Careggi), Camilla Cona (IOV

- Istituto Oncologico Veneto), Corrado Bottini (Sant'Antonio Abate Hospital, Gallarate), Giovanni Pascale (Azienda Ospedaliero-Universitaria di Ferrara), Giuseppe Rotunno (Nicola Giannettasio Hospital, Rossano), Leonardo Solaini (University Of Brescia, Spedali Civili Di Brescia), Marco Maria Pascale (Fondazione Policlinico Universitario 'Agostino Gemelli'), Margherita Notarnicola (University Of Bari 'Aldo Moro'), Mario Corbellino (Ospedale Luigi Sacco Milano), Michele Sacco (Federico II University of Naples), Paolo Ubiali (Azienda per L'Assistenza Sanitaria N. 5 'Friuli Occidentale', Pordenone), Roberto Cautiero (Second University Of Naples), Tommaso Bocchetti, (Sant'Andrea Hospital, Sapienza University of Rome), Elena Muzio, (S. Andrea Hospital, Poll-Asl 5, La Spezia); Vania Guglielmo (Policlinico Umberto I, Emergency Surgery Department); Eugenio Morandi (Ospedale di Rho – ASST Rhodense), Patrizio Mao (San Luigi Gonzaga Hospital, Orbassano); Emilia De Luca (Department of Medical and Surgical Sciences, Policlinico Universitario Mater Domini Campus Salvatore Venuta, Catanzaro), Margherita Notarnicola (Azienda Ospedaliero Universitaria Consorziale Policlinico Di Bari).

Jordan: Farah Mahmoud Ali (Jordan University Hospital);

Lithuania: Justas Žilinskas (Klaipeda Republic), Kestutis Strupas (Vilnius University Hospital), Paulius Kondrotas (Taurage County Hospital), Robertas Baltrunas (Rokiskis District Municipality Hospital); Juozas Kutkevicius (Department Of General Surgery, Lithuanian University Of Health Sciences), Povilas Ignatavicius (Hospital Of Lithuanian University Of Health Sciences Kaunas Clinics);

Malaysia: Choy Ling Tan (Hospital Sultanah Aminah), Jia Yng Siaw (Hospital Sibu), Sir Young Yam (Penang Medical College); Ling Wilson (Sarawak General Hospital), Mohamed Rezal Abdul Aziz (University Malaya Medical Centre);

Malta: John Bondin (Mater Dei Hospital);

Mexico: Carmina Diaz Zorrilla (Hospital Espanol De Veracruz);

Morocco: Anass Majbar (Centre Hospitalier Ibn Sina Rabat);

Nigeria: Danjuma Sale (Barau Dikko Teaching Hospital), Lawal Abdullahi (Kano Aminu), Olabisi Osagie (University Of Abuja Teaching Hospital), Omolara Faboya (Lagos Lasuth); Adedeji Fatuga (Lagos Luth), Agboola Taiwo (Babcock University Teaching Hospital), Emeka Nwabuoku (Ahmadu Bello University Teaching Hospital);

Norway: Marte Bliksøen (Oslo University Hospital);

Pakistan: Zain Ali Khan (Bahawal Victoria Hospital, Bahawalpur);

Paraguay: Jazmin Coronel (Hospital de Clínicas, II Cátedra de Clínica Quirúrgica, Universidad Nacional de Asunción)

Peru: Cesar Miranda (Hospital Nacional Cayetano Heredia), Idelso Vasquez (Lima Almenara), Luis M. Helguero-Santin (Hospital Regional III Jose Cayetano Heredia – Piura);

Rwanda: Jennifer Rickard (Centre Hospitalier Universitaire De Kigali);

Romania: Aurel Mironescu (Spitalul Clinic De Copii Brasov);

Saint Kitts and Nevis: Adesina Adedeji (Joseph N France Hospital);

Saudi Arabia: Saleh Alqahtani (King Fahad General Hospital);

South Africa: Max Rath (Groote Schuur Hospital), Michael Van Niekerk (New Somerset Hospital), Modise Zacharia Koto (Dr George Mukhari Academic Hospital); Roel Matos-Puig (Stanger Hospital);

Sweden: Leif Israelsson (Sundsvall);

Switzerland: Tobias Schuetz (Kantonsspital Olten);

Turkey: Mahmut Arif Yuksek (Ondokuz Mayis University), Meric Mericliler (Acibadem University School of Medicine, Atakent Hospital), Mehmet Uluşahin (Karadeniz Technical University Farabi Hospital);

United Kingdom: Bernhard Wolf (Raigmore Hospital Inverness), Cameron Fairfield (Royal Infirmary Of Edinburgh), Guo Liang Yong (Perth Royal Infirmary), Katharine Whitehurst (Royal Devon And Exeter), Michael Wilson (Ninewells Hospital And

Medical School), Natalie Redgrave (John Radcliffe Hospital, Oxford); Caroluce K Musyoka (Royal Alexandra Hospital), James Olivier (Royal United Hospital Bath), Kathryn Lee (Queen Elizabeth Birmingham), Michael Cox (Royal Derby Hospital), Muhamed M H Farhan-Alanie (Inverclyde Royal Hospital), Rory Callan (North Middlesex University Hospital)
Zambia: Chali Chibuye (Ndola Central Hospital)

Protocol Translators:
Arabic, Tebian Hassanein Ahmed Ali, Syrine Rekhis, Muna Rommaneh, Oday Halhouli**;**
Chinese, Zi Hao Sam**;**
French, Lawani Ismaïl**;**
Greek, Vasileios Kalles**;**
Italian, Francesco Pata, Gabriela Elisa Nita, Federico Coccolini, Luca Ansaloni**;**
Portuguese, Thays Brunelli Pugliesi, Gabriel Pardo**;**
Spanish, Ruth Blanco
